# Research trends in pharmacological modulation of tumor‐associated macrophages

**DOI:** 10.1002/ctm2.288

**Published:** 2021-01-13

**Authors:** Neng Wang, Shengqi Wang, Xuan Wang, Yifeng Zheng, Bowen Yang, Juping Zhang, Bo Pan, Jianli Gao, Zhiyu Wang

**Affiliations:** ^1^ The Research Center for Integrative Medicine School of Basic Medical Sciences Guangzhou University of Chinese Medicine Guangzhou Guangdong China; ^2^ The Research Center of Integrative Cancer Medicine Discipline of Integrated Chinese and Western Medicine The Second Clinical College of Guangzhou University of Chinese Medicine Guangzhou Guangdong China; ^3^ Guangdong‐Hong Kong‐Macau Joint Lab on Chinese Medicine and Immune Disease Research Guangzhou University of Chinese Medicine Guangzhou Guangdong China; ^4^ Guangdong Provincial Key Laboratory of Clinical Research on Traditional Chinese Medicine Syndrome Guangdong Provincial Hospital of Chinese Medicine Guangdong Provincial Academy of Chinese Medical Sciences Guangzhou Guangdong China; ^5^ Academy of Traditional Chinese Medicine Zhejiang Chinese Medical University Hangzhou Zhejiang China

**Keywords:** chemokine–chemokine receptor, exosomeimmune suppression, metabolism, tumor‐associated macrophages, tyrosine kinase receptor

## Abstract

As one of the most abundant immune cell populations in the tumor microenvironment (TME), tumor‐associated macrophages (TAMs) play important roles in multiple solid malignancies, including breast cancer, prostate cancer, liver cancer, lung cancer, ovarian cancer, gastric cancer, pancreatic cancer, and colorectal cancer. TAMs could contribute to carcinogenesis, neoangiogenesis, immune‐suppressive TME remodeling, cancer chemoresistance, recurrence, and metastasis. Therefore, reprogramming of the immune‐suppressive TAMs by pharmacological approaches has attracted considerable research attention in recent years. In this review, the promising pharmaceutical targets, as well as the existing modulatory strategies of TAMs were summarized. The chemokine–chemokine receptor signaling, tyrosine kinase receptor signaling, metabolic signaling, and exosomal signaling have been highlighted in determining the biological functions of TAMs. Besides, both preclinical research and clinical trials have suggested the chemokine–chemokine receptor blockers, tyrosine kinase inhibitors, bisphosphonates, as well as the exosomal or nanoparticle‐based targeting delivery systems as the promising pharmacological approaches for TAMs deletion or reprogramming. Lastly, the combined therapies of TAMs‐targeting strategies with traditional treatments or immunotherapies as well as the exosome‐like nanovesicles for cancer therapy are prospected.

## Graphical abstract

The biofunctions and clinical implications of TAMs in cancer progression as well as the current pharmaceutical targets in TAMs regulation were summarized. The latest advancements of agents that were effective in TAMs depletion or reprogramming were summarized and discussed. The promising future of exosomal signaling for TAMs‐targeted therapy was discussed and expected.

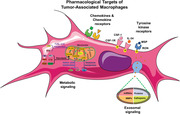

**Abbreviations**
TAMs: tumor‐associated macrophages
TME: tumor microenvironment
PAMP: pathogen‐associated patterns
LPS: lipopolysaccharides
TNF‐α: tumor necrosis factor α
GM‐CSF: granulocyte‐macrophage colony‐stimulating factor
IL: interleukin
DAMP: damage‐associated patterns
ARG1: arginase
DMBA: 12‐dimethylbenz‐(a)anthracene
sCD163: soluble CD163
irAEs: immune‐related adverse events
VEGFA: vascular endothelial growth factor A
ADM: adrenomedullin
bFGF: basic fibroblast growth factor
PDGF: platelet‐derived growth factor
MIF: macrophage‐inhibitory factor
PAF: platelet‐activating factor
monocyte chemoattractant protein‐1 MCP‐1: monocyte chemoattractant protein‐1
MMPs: matrix metalloproteinases
PD‐L1: program cell death ligand 1
CTLA‐4: cytotoxic T‐lymphocyte‐associated protein 4
OS: overall survival
DFS: disease free survival
PSGL‐1: P‐selectin glycoprotein ligand‐1
ICAM‐1: intracellular cell adhesion molecule‐1
ERK5: extracellular‐regulated protein kinase 5
EMT: epithelial–mesenchymal transition
ECM: extracellular matrix
PMN: premetastatic niche
GPCRs: G‐protein‐coupled receptors
MDSCs: myeloid‐derived suppressor cells
CTCL: cutaneous T‐cell lymphoma
IFNs: interferons
RTKs: Tyrosine kinase receptors
CSF‐1R: colony stimulation factor‐1 receptor
MSP: macrophage‐stimulating protein
MerTK: Mer tyrosine kinase
HIF: Hypoxia‐inducible factors
BMDMs: bone marrow–derived macrophages
PPAR: peroxisome proliferator‐activated receptor
PDK: pyruvate dehydrogenase kinase
IDO: indoleamine‐2, 3‐dioxygenase
RAGE: receptor for advanced glycation end‐product
DAG: diacylglycerol
MG: methylglyoxal
AGEs: advanced glycation end products
ELISA: enzyme‐linked immunosorbent assays
dt‐GCTs: diffuse‐type tenosynovial giant‐cell tumors
exoASO: exosome carrying antisense oligonucleotide
TRL7/8: toll‐like receptor 7 and 8
SIRPα: signal regulatory protein‐α
TREM2: triggering receptor expressed on myeloid cells 2
ERα: estrogen receptor α
ERK: extracellular signal‐regulated kinase
PKC: protein kinase C
PI3K: phosphoinositide‐3 kinase
AKT: the serine/threonine kinase
MAPK: mitogen‐activated protein kinase
mTOR: mammalian/mechanistic target of rapamycin
NF‐κB: nuclear factor κB
PDGF‐BB: platelet‐derived growth factor subunit B homodimer
Ezh2: enhancer of zeste homolog 2
CSCs: cancer stem cells
ECs: endothelial cells
STAT3: signal transducer and activator of transcription 3
CSN5: COP9 signalosome subunit 5
FOXP3: forkhead box P3
KLF5: kruppel‐like factor 5
TGF‐β: transforming growth factor β
GSK‐3β: Glycogen synthase kinase‐3β
ANXA2: annexin A2
PITPNM3: phosphatidylinositol transfer protein 3
FOXO1: Forkhead box O1
CEBPB: CCAAT/enhancer binding protein beta
TRAIL: tumor necrosis factor‐related apoptosis‐inducing ligand
ABCB1: ATP binding cassette subfamily B member 1
HuR: human antigen R
JAK2: Janus protein tyrosine kinases
Pyk2: proline‐rich tyrosine kinase 2
ELMO1: engulfment and cell motility 1
SAPK: stress‐activated protein kinase
JNK: Jun N‐terminal kinase
HDAC1: histone deacetylase 1
TACE: tumor necrosis factor alpha‐converting enzyme
SOX4: SRY‐box transcription factor 4
S100A8/9: S100 calcium binding protein A8/9
TAK1: transforming growth factor‐beta‐activated kinase 1
EGFR: epidermal growth factor receptor
FAK: focal adhesion kinase
RhoA: ras homolog family member A
PGE2: Prostaglandin E2
ARHGAP10: Rho GTPase Activating Protein 10
MALAT1: metastasis‐associated lung adenocarcinoma transcript 1
TOPK: T‐LAK Cell‐originated Protein Kinase
JNK: c‐Jun N‐terminal kinase
MST1R: macrophage‐stimulating 1 receptor
HK2: hexokinase 2
GPR132: G protein‐coupled receptor 132
REDD1: DNA damage response‐1
HMGB1: high‐mobility group box 1
RIPK3: receptor‐interacting protein kinase‐3
FABP: fatty acid binding protein
MCAD: medium‐chain acyl‐coA dehydrogenase
GS: glutamine synthetase
ARG: arginase
FPN: ferroportin
LCN: lipocalin
BRG1: brahma‐related gene 1
LIPA: lipase A
PTEN: phosphatase and tensin homolog
TGFBR3: transforming growth factor beta receptor 3

## 1 BACKGROUND

Cancer is a heterogeneous disease that is composed of numerous cell types, including both cancer and noncancer cells. Although macrophage content is highly variable in different tumor entities, macrophages are among the most prominent tumor‐associated noncancer cell type in the tumor microenvironment (TME),^1^, ^2^ known as tumor‐associated macrophages (TAMs). Current evidence suggests that TAMs engage in complex network interactions with cancer stem cells, cancer cells, endothelial cells, fibroblasts, T cells, B cells, and natural killer cells.^3^ Their cross talks accelerate the formation of the immune‐suppressive TME that not only stimulates cancer cell proliferation, neoangiogenesis, lymphangiogenesis, drug resistance, and distant metastasis, but also further recruits macrophages to establish a vicious feedback loop that continually reinforces the immune‐suppressive TME. Recently, multiple signaling pathways have been identified as critical nodes mediating TAMs polarization and interactions with malignant cells. Small molecule inhibitors or monoclonal antibodies targeting TAMs signaling have been demonstrated to effectively inhibit cancer development and metastasis. Therefore, TAMs have emerged as a central drug target for cancer therapy. Here, our review focuses on discussing the biological functions of TAMs, critical signaling and targets regulating TAMs activities, and current therapeutic strategies for treating malignancies that affect TAMs.

**HIGHLIGHT**

- The biofunctions and clinical implications of TAMs in cancer progression were summarized.
- The current pharmaceutical targets in TAMs regulation were introduced and discussed.
- The latest advancements of agents that were effective in TAMs depletion or reprogramming were summarized and discussed.
- The promising future of exosomal signaling for TAMs‐targeted therapy was discussed and expected.

## 2 MACROPHAGE POLARIZATION IN CANCER

Since the first description by llya llyich Mechnikov in 1882, phagocytes have been reported to be present throughout the body and to perform specific biological functions. For example, liver macrophages (Kupffer cells) eliminate pathogenic and waste products from circulation.^4^ Brain‐resident macrophages (microglia) contribute to the maintenance of engrams (i.e., the neural bases of memories)^5^ by facilitating synaptic pruning. Langerhans cells in the skin are responsible for activating local inflammatory reactions and clearing pathogenic substances.^6^ Phagocytes can also be infiltrated into the TME to perform pro‐ and/or antitumor functions. Usually, macrophages are considered as a plastic cell type capable of activating or polarizing into different statuses. Macrophages change their activation or polarization statuses in response to any potential entity, which is capable of being recognized by macrophages. The common stimuli including growth factors, cytokines/chemokines, hypoxia, exosomes, microbes, microbial products, nucleotide derivatives, antibody‐Fc receptor stimulation, glucocorticoids, infection, and phagocytosis.^7^ Therefore, it is hard to establish the common nomenclatures or standards for describing the activation or polarization properties of macrophages induced by various stimuli.^7^ There are mainly four definitions of macrophage activation or polarization statuses, including terms such as M1‐like and M2‐like, alternative and classical activation, “regulatory” macrophages, and subdivisions originating from the parent terms.^7^ In 2012, Mills proposed the M1‐like/M2‐like dichotomy to describe the two major and opposing activities of macrophages.^8^ At present, this definition method of macrophage activation or polarization statuses is widely used by the majority of researchers. M1‐like polarization is reported to be induced by pathogen‐associated patterns, including lipopolysaccharides (LPS), interferon‐γ, granulocyte‐macrophage colony‐stimulating factor (GM‐CSF), and tumor necrosis factor α (TNF‐α). M1‐like activation causes the release of various cytokines, such as interleukin 6 (IL‐6), IL‐1β, and TNF‐α, that facilitate a proinflammatory response to defense against pathogenic insults.^9^ In contrast, the M2‐like phenotype is induced by damage‐associated patterns (DAMPs) from cytokines such as IL‐4 and IL‐13, which subsequently activate the JAK‐STAT pathway and turn on the expression of anti‐inflammatory cytokines, such as resistin‐like molecule α, IL‐10, and arginase 1 (ARG1).^10^ These signals further accelerate the remodeling of the TME to promote cancer angiogenesis, growth, and immune suppression. Usually, M1‐like is considered to be an inhibitory phenotype due to its function in promoting Th1 responses that exert tumoricidal and microbicidal functions, whereas M2‐like represents a restorative phenotype due to its effects in activating Th2 responses that contribute to tissue remodeling/repair, angiogenesis, immunosuppression, and tumor progression.^11^ Generally, both M1‐like and M2‐like phenotypes can exist within the same microenvironment; therefore, the molecular targets controlling the polarization balance are considered as an important approach for cancer therapy. The polarization biomarkers of M1‐like macrophages include CD86 and CD80, while the polarization biomarkers of M2‐like macrophages include CD163, CD204, CD206, CD115, and CD301.^12^ Particularly, CD163 can simultaneously be present both in a membrane‐bound form on M2‐like phenotype TAMs and a soluble form in plasma. Because of its macrophage‐specific expression characteristic and highly elevated concentrations during various pathological conditions, soluble CD163 (sCD163) level is also regarded as a reliable biomarker for monitoring macrophage activity.^13^ For example, CD163^+^ TAMs were reported to express high levels of immune‐checkpoint molecules PD1 and PD‐L1. Blocking PD1/PD‐L1 signaling in CD163^+^ TAMs with antibodies could induce M1‐like polarization and increase macrophage phagocytosis, reduce tumor burden and prolong survival,^14^, ^15^ and result in an increased release of sCD163 in the lesional skin of melanoma.^13^ Meanwhile, Fujimura *et al*. identified serum sCD163 as a reliable predictive biomarker for immune‐related adverse events (irAEs) and efficacy of nivolumab in patients with advanced melanoma.^16^, ^17^ These findings suggest that macrophage polarization modulation may also be the potential pharmacological mechanism of immune‐checkpoint inhibitors. The combination treatment of macrophage‐targeted strategy and PD‐1/PD‐L1 blockade may provide a synergetic antitumor efficacy, which deserves further investigations.

## 3 CLINICAL IMPLICATIONS OF TAMs IN CANCER DEVELOPMENT

**FIGURE 1:**
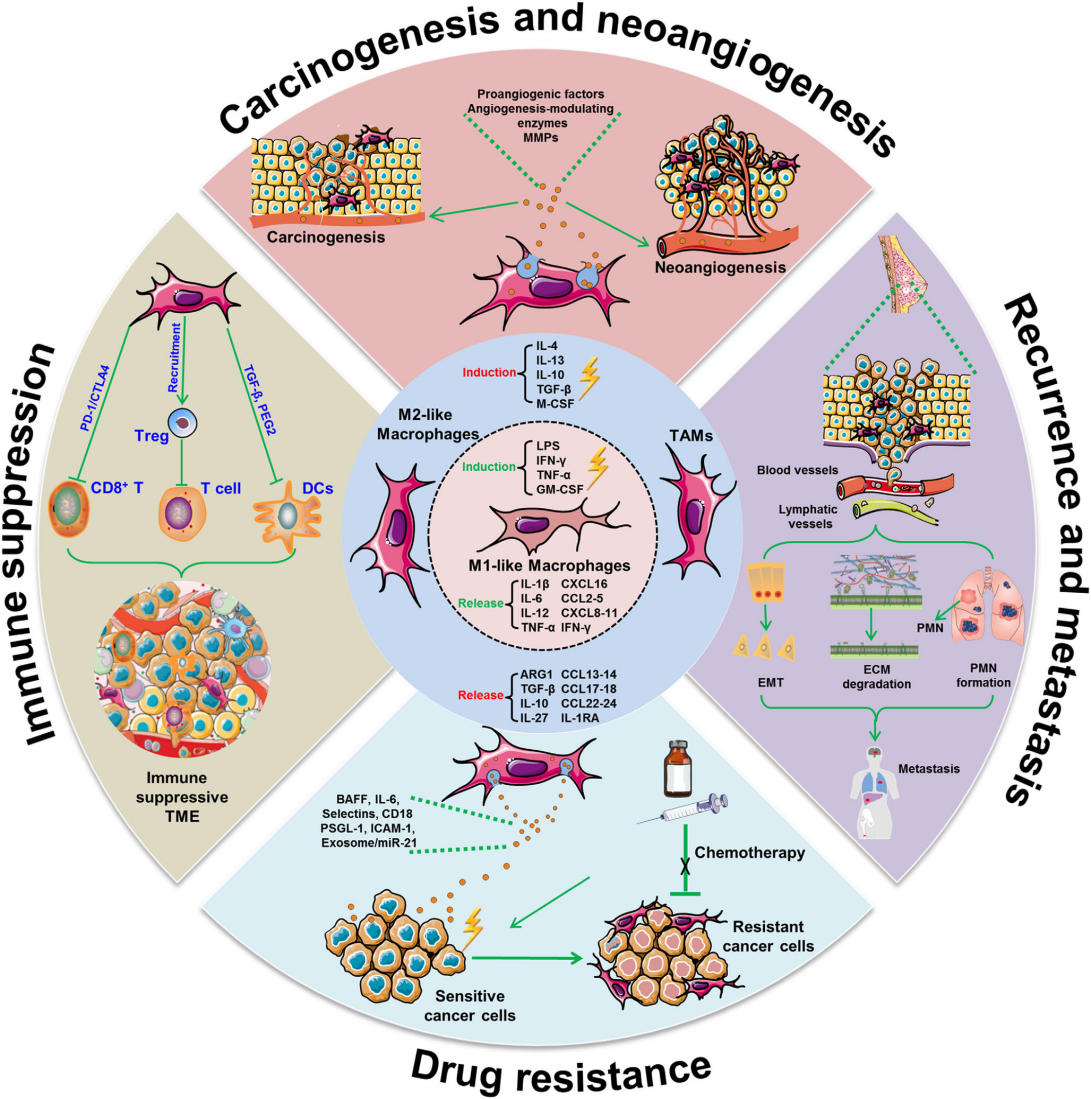
Pro‐oncogenic role of TAMs in cancer development. Macrophages could be differentiated into M1‐like and M2‐like phenotypes under different stimuli. M1‐like macrophages were usually considered as a “killer” phenotype while M2‐like macrophages were known as the “healing” phenotype. M2‐like macrophages have been reported to participate in the process of carcinogenesis and neoangiogenesis *via* releasing proangiogenic factors, enzymes, and MMPs. Meanwhile, M2‐like macrophages promoted cancer recurrence and metastasis *via* modulating epithelial–mesenchymal transition, extracellular matrix degradation, and facilitating premetastatic niche formation. On the other hand, M2‐like macrophages could induce chemoresistance by exosomal signaling or cell–cell contact. Most importantly, M2‐like macrophages contributed to establishing the immune suppression microenvironment by elevating the PD‐1/CTLA4 signaling or inhibiting the bio‐functions of cytotoxic T cells or dendritic cells

Over the past decade, TAMs have attracted rising research attention for their modulatory potential on angiogenesis, cytokines, and immune regulation that either inhibit or facilitate tumor progression. Clinically, elevated infiltration levels of M1‐like macrophages within tumor tissues predict a favorable prognosis, while elevations in the M2‐like macrophages usually predict poor outcomes.^18^ A tissue microarray analysis of 553 primary non‐small cell lung cancer patients revealed that M1‐like macrophages in metastatic lymph nodes were a predictor of improved survival.^19^ In esophageal cancer patients, cases with high CD163 and CD204 expression levels showed a significantly shorter overall survival than those with comparatively lower CD163 and CD204 levels.^20^ In addition, in triple‐negative breast cancer patients with either high CD163 expression or low CD163 expression, the 5‐year overall survival ratio was 72.3% or 82.5%, respectively.^21^ These clinical observations have been well supported by experimental studies using macrophage depletion or overexpression strategies. For example, genetic ablation of GM‐CSF in different murine tumor models—such as the MMTV‐PyVT^+/‐^ breast cancer model, the spontaneous colon cancer model, and the osteosarcoma xenotransplant model—significantly reduces M2‐like macrophage density in solid tumors, which in turn inhibits cancer growth and progression.^22^, ^23^, ^24^ In addition, macrophage depletion with clodronate‐encapsulated liposomes also yields a significant reduction of tumor volume in several malignancies.^25^, ^26^ In contrast, overexpression of CSF‐1 remarkably increases TAMs recruitment and accelerates cancer development and metastasis.^27^ It should be noted that the effect of TAMs on tumor tumorigenesis and progression may fluctuate due to their differentiation heterogeneity.^28^ Based on these clinical observations and experimental findings, numerous studies have investigated the underlying mechanisms of TAMs in controlling cancer development. At present, M2‐like macrophages have been found to participate in all steps of malignant development including carcinogenesis, neoangiogenesis, overall immune suppression, drug resistance, and later recurrence and/or metastasis (Figure 1).

### 3.1 TAMs contribute to carcinogenesis and neoangiogenesis

TAMs have been found to be involved in the first step of carcinogenic lesion formation during neoplasia. Macrophage infiltration has been found to be upregulated in a murine chemically induced skin carcinogenesis model.^29^ Similarly, a massive accumulation of CD206^+^ or ARG1^+^ macrophages has also been found in an inflammation‐mediated skin tumorigenesis mice model, while macrophage ablation has been shown to significantly reduce tumor incidence.^30^ In an EGFR‐driven lung carcinogenesis model, sustained macrophage recruitment has been observed and macrophage depletion causes a significant reduction in tumor burden.^31^ Neoangiogenesis is also a critical step during carcinogenesis, in which macrophage infiltration is also involved. Various studies have suggested that TAMs are predominantly located near the blood vessels of malignant solid tumors, and TAMs numbers are usually positively correlated with blood vessel density.^32^, ^33^, ^34^, ^35^ Functional studies have also demonstrated that TAMs elimination causes the reduction of neoangiogenesis,^36^ while TAMs enhancement aggravates this process.^36^ Mechanistic studies imply that TAMs can release multiple proangiogenic factors, such as vascular endothelial growth factor A (VEGF‐A), macrophage‐inhibitory factor (MIF), adrenomedullin (ADM), platelet‐activating factor (PAF), platelet‐derived growth factor (PDGF), basic fibroblast growth factor (bFGF), and TGF‐β, as well as numerous cytokines such as TNF‐α, IL‐1, IL‐8, and monocyte chemoattractant protein‐1 (MCP‐1).^37^, ^38^, ^39^, ^40^ Additionally, TAMs also release numerous angiogenesis‐modulating enzymes including iNOS,^41^ COX‐2, and matrix metalloproteinases (MMPs),^42^, ^43^, ^44^ all of which have been associated in matrix degradation and endothelial cell invasion.

### 3.2 TAMs facilitate the formation of the immune‐suppressive microenvironment

TAMs recruitment not only supports cancer growth *via* neoangiogenesis induction but also facilitates the establishment of the immune‐suppressive microenvironment. Recent studies have suggested that TAMs express PD‐L1, PD‐L2, CD86, and CD80, all of which induce CD8^+^ T cell dysfunction upon binding to immune‐checkpoint receptors such as PD1 or cytotoxic T‐lymphocyte‐associated protein 4 (CTLA4).^44^, ^45^ In addition, TAMs release multiple cytokines, enzymes, and chemokines that inhibit T‐cell activity through natural regulatory T cell recruitment or L‐arginine depletion in the TME. For example, IL‐10 produced by TAMs could suppress IL‐12 secretion from myeloid cells and promote Th2‐type immune response.^46^ The secretion of TGF‐β and PGE2 can impair the maturation process of dendritic cells, which subsequently compromise the balance between innate and adaptive immunity.^47^, ^48^ Immune‐checkpoint inhibitors have revealed successful therapeutic responses in multiple malignant tumors such as melanoma and lung cancers.^49^ Unfortunately, only approximately 20% of cancer patients respond to immunotherapy, and mixed responses can limit therapeutic efficacies and lead to local recurrences and/or distant metastases.^50^ Given the abundance and immune‐suppressive properties of TAMs, targeting TAMs has been suggested as a promising approach to promote the efficacy of checkpoint antagonists. For example, anti‐PD1/anti‐CTLA4 treatment can decrease pancreatic tumor growth by approximately 50%, while their combination with PLX3397 (CSF1R inhibitor) can dramatically attenuate tumor expansion and even results in tumor regression by 15%.^51^ FcγR is a receptor typically expressed by TAMs. Similarly, a PD1 antibody also results in tumor growth inhibition in colon cancer xenografts, although this efficacy typically varies among mice. Strikingly, when a PD1 antibody and FcγR antagonist were simultaneously administrated, tumor growth was completely inhibited in all mice.^52^ Additionally, suppressing IL‐10 signaling in TAMs can promote CD8^+^ T cell‐mediated antitumor immune responses in breast cancer receiving chemotherapy.^46^ Given these encouraging preclinical results, utilization of TAMs‐targeting strategies is promising for future clinical application as a combination therapy with checkpoint inhibitors.

### 3.3 TAMs aggravate cancer drug resistance

TAMs density is also closely correlated with therapeutic responses and is highlighted in cancer drug resistance. Multiple studies have found that TAMs populations are enriched following toxic cancer‐killing treatments. In aromatase‐inhibitor–resistant breast cancer, TAMs density is much higher in primary tumor tissues and significantly correlates with decreased disease‐free survival (DFS) and overall survival (OS).^53^ TAMs infiltration was also found to be elevated following chemotherapy in a pancreatic ductal adenocarcinoma model, and TAMs pretreatment could render pancreatic cancer cells resistant to gemcitabine whereas this efficacy was strikingly enhanced by TAMs depletion *via* clodronate liposomes.^54^ In colon cancer, TAMs infiltration has frequently detected been in chemoresistant patients and is significantly associated with poor survival.^55^ The macrophage marker, CD68, is positively correlated with the expression of MDR1. Notably, blocking IL‐6 secreted from TAMs is capable of resensitizing colon cancer cells to chemotherapeutic drugs. Meanwhile, B‐cell activating factor released by TAMs is also effective in mediating bortezomib resistance in myeloma. In addition to cytokine secretion, ligand–receptor interactions between cells are also involved in mediating chemoresistance.^56^ In a macrophage–myeloma contact model, plasma‐membrane protein/gene profiling assays have demonstrated that CD18 and selectins in macrophages remarkably contribute to chemoresistance *via* combining with intracellular cell adhesion molecule‐1 (ICAM‐1) and P‐selectin glycoprotein ligand‐1 (PSGL‐1), respectively, in myeloma cells. Pharmacological inhibition or genetic knockdown of PSGL‐1 or ICAM‐1 signaling in these myeloma cells can diminish the protective effects of TAMs from cytotoxic cancer‐killing agents including melphalan, doxorubicin, and bortezomib. Recent findings also suggest that exosomes are also implicated in TAMs‐induced chemoresistance. It is found that macrophages‐derived exosomal miR‐21 signal could be directly delivered into gastric cancer cells, and thus results in apoptosis inhibition and cisplatin resistance of gastric cancer cells.^57^ Therefore, it may be worthwhile to apply TAMs‐targeting therapies with traditional cancer‐killing strategies in future clinical applications.

### 3.4 TAMs accelerate cancer recurrence and metastasis

Local recurrence and distant metastasis are not solely determined by the malignant behavior of cancer cells because recent studies have suggested that stromal cells, particularly TAMs, act as an important driving force in these processes. For example, Giurisato *et al*. proved that extracellular‐regulated protein kinase 5 (ERK5)‐mediated macrophage proliferation could support melanoma invasiveness and metastasis *in vivo*, suggesting TAMs renewal as an integral component of tumor metastasis.^58^ Mechanistically, numerous researches have demonstrated the crucial role of TAMs in regulating the epithelial–mesenchymal transition (EMT) process.^59^, ^60^, ^61^ Cocultured hepatocellular cancer cells with TAMs could enhance the expression levels of mesenchymal markers including N‐cadherin and vimentin, whereas attenuating the epithelial marker E‐cadherin.^60^ A similar phenomenon was also observed in breast, gastric, colon, and pancreatic malignancies.^62^ Biologically, multiple TAMs‐secreted cytokines including IL‐1β, IL‐8, EGF, TNF‐α, and TGF‐β have been validated to promote the EMT process.^60^, ^63^, ^64^ Meanwhile, TAMs can secrete various kinds of proteolytic enzymes including MMPs, cathepsins, and serine proteases to degrade the extracellular matrix (ECM).^65^, ^66^ For instance, cathepsin B and S secreted by TAMs were found to be critical in promoting pancreatic cancer invasion.^67^ An earlier study also demonstrated that TAMs synthesize SPARC/osteonectin to deposit collagen IV, resulting in enhanced tumor invasion and adhesion to other ECM components.^68^ TAMs have also been shown to be necessary for facilitating cancer cell intravasation and extravasation. Multiphoton intravital imaging techniques have shown that intravasating cancer cells are invariably accompanied by a macrophage within one cell diameter.^69^ Interestingly, macrophages loss can significantly impair the extravasation rates and metastasis of cancer cells.^70^ Additionally, TAMs have also been found to be critical in supporting the survival of cancer cells in the circulation. TAMs depletion *via* genetic methods can dramatically suppress cancer cell survival in pulmonary capillaries as well as the subsequent lung metastasis formation.^71^, ^72^ The underlying molecular mechanisms of their synergistic interaction are as follows. First, TAMs may trigger PI3K/Akt survival signaling in cancer cells to circumvent proapoptotic cytokines such as TRAIL.^73^ Second, TAMs may alleviate survival stress from NK or cytotoxic T cells in the circulation.^72^ Following extravasation, it has been found that TAMs are one of the key determinants in establishing the premetastatic niche (PMN).^74^ Circulatory or residential TAMs can release chemokines that guide the localization of cancer cells into the PMN with increased expression levels of MMPs, fibronectins, S100A8, and S100A9.^75^, ^76^, ^77^ Our recent report demonstrated that TAMs‐derived CXCL1 exerted an important role in recruiting breast cancer cells into the PMN.^78^ Thus, TAMs act as an indispensable factor in fostering cancer cells traveling from the primary site to metastatic lesions. Molecular elucidation of TAMs regulation in cancer development is thus warranted and critical for further developing cancer‐targeting strategies.

## 4 PHARMACEUTICAL TARGETS OF TAMS

To date, several key pathways including chemokine–chemokine receptor signaling, tyrosine kinase receptor (RTK) signaling, metabolic signaling, and exosomal signaling have been highlighted in determining the biofunctions of TAMs. The promising pharmacological targets of each signaling were described as following and summarized in Table 1.

**TABLE 1:** The pharmaceutical targets of TAMs

Signaling	Targets	Tumors	Mechanisms	References
Chemokine–chemokine receptor signaling	CCL2/ CCR2	Breast cancer	Facilitating cancer progression via activation of ERα and PI3K/AKT/NF‐κB signaling; inducing tamoxifen resistance by activating PI3K/Akt/mTOR signaling; enhancing growth and cell‐cycle progression through SRC and PKC activation	^177^, ^178^, ^179^
	CCL2/ CCR2	Prostate cancer	Recruiting and regulating macrophages via the MAPK/ ERK signaling pathway; protecting cancer cells from autophagic death via the PI3K/Akt/survivin pathway; promoting cancer progression through the induction of MMP‐2 activity	^180^, ^181^, ^182^
	CCL2/ CCR2	Liver cancer	Inducing invasion and EMT through activation of the Hedgehog pathway; inducing migration and invasion depending on MMP‐2 and MMP‐9;	^183^, ^184^
	CCL2/ CCR2	Lung cancer	Increasing invasion via ERα; promoting TAMs infiltration via NF‐κB/CCL2 signaling; promoting invasion mediated by autocrine loop of PDGF‐BB	^185^, ^186^, ^187^
	CCL2/ CCR2	Ovarian cancer	Promoting peritoneal metastasis through P38‐MAPK pathway	^188^
	CCL2/ CCR2	Gastric cancer	Inhibiting proapoptotic autophagy by activating PI3K‐Akt‐mTOR signaling	^189^
	CCL5/CCR1	Colorectal cancer	Promoting cancer progression through CCL5/β‐catenin/Slug pathway	^190^
	CCL5/CCR1	Prostate cancer	Promoting invasion by increasing MMP‐2/9 and activating ERK/Rac signaling	^191^
	CCL5/CCR5	Lung cancer	Increasing migration via PI3K/AKT/NF‐κB signaling; Promoting metastasis and macrophages infiltration via EZH2	^192^, ^193^
	CCL5/CCR5	Breast cancer	Promoting immune cell infiltration driven by TNFα/NF‐κB signaling; enhancing Trastuzumab resistance by ERK pathway activation; promoting cancer proliferation through the mTOR pathway	^194^, ^195^, ^196^
	CCL5/CCR5	Ovarian cancer	Promoting invasion and migration via NF‐κB–mediated MMP‐9 upregulation; mediating differentiation of CSCs into ECs via the NF‐κB and STAT3 signaling	^197^, ^198^
	CCL5/CCR5	Liver cancer	Inducing EMT through activation of the Akt pathway	^199^
	CCL5/CCR5	Pancreatic cancer	Recruiting Treg cells via cancer‐FOXP3; inducing proliferation of cancer cells through F‐actin polymerization	^200^, ^201^
	CCL5/CCR5	Colorectal cancer	Enhancing TGF‐β‐mediated killing of CD8^+^ T cells; Facilitating immune escape via the p65/STAT3‐CSN5‐PD‐L1 pathway	^91^, ^202^
	CCL5/CCR5	Gastric cancer	Enhancing aberrant DNA methylation via STAT3 signaling; inducing invasion and proliferation via KLF5	^203^, ^204^
	CCL5/CCR5	Melanoma	Activating apoptotic pathway involving release of cytochrome c and activation of caspase‐9 and caspase‐3	^205^
	CCL18	Breast cancer	Inducing EMT via PI3K/Akt/GSK3β/Snail signaling through AnxA2; recruiting Treg cells and promoting metastasis and via PITPNM3; enhancing EMT via N‐Ras/ERK/PI3K/NF‐κB/Lin28b signaling	^206^, ^207^, ^208^
	CCL18	Oral squamous cell carcinoma	Promoting growth and metastasis by activating the JAK2/STAT3 signaling; promoting migration via mTOR signaling through Slug	^209^, ^210^
	CCL18	Ovarian cancer	Promoting migration through Pyk2 signaling or mTORC2 signaling	^211^, ^212^
	CCL18	Lung cancer	Promoting migration and invasion via Nir1 through Nir1‐ELMO1/DOC180 signaling	^213^
	CCL18	Bladder cancer	Promoting lymphangiogenesis by increasing the production of VEGF‐C and MMP‐2	^214^
	CCL18	Pancreatic cancer	Promoting progression and the Warburg effect via NF‐κB/VCAM‐1 pathway	^215^
	CCL18	Gastric cancer	Promoting invasion and migration via ERK1/2/NF‐κB signaling	^216^
	CCL20/CCR6	Lung cancer	Promoting migration via ERK1/2‐MAPK and PI3K pathways; promoting progression mediated by lncRNA‑u50535 through CCL20/ERK signaling	^217^, ^218^
	CCL20/CCR6	Renal cell carcinoma	Inducing EMT through Akt activation	^219^
	CCL20/CCR6	Colorectal cancer	Promoting chemoresistance via FOXO1/CEBPB/NF‐κB signaling; promoting growth and metastasis mediated by lncRNA‑u50535; promoting proliferation and migration through ERK‐1/2, SAPK/JNK, and Akt signaling	^220^, ^221^, ^222^
	CCL20/CCR6	Pancreatic cancer	Increasing TRAIL resistance via RelA‐CCL20 pathway; promoting migration, EMT, and invasion through PI3K/AKT‐ERK1/2 signaling	^223^, ^224^
	CCL20/CCR6	Breast cancer	Increasing chemoresistance via ABCB1/ NF‐κB signaling; promoting bone metastasis mediated by HuR; inducing EMT via PKC‐α/Src/Akt/NF‐kB/Snail signaling; promoting invasion by PKC‐α through EGFR and ERK1/2/MAPK pathway	^225^, ^226^, ^227^, ^228^
	CCL20/CCR6	Gastric cancer	Inducing EMT mediated by CRKL via Akt pathway	^229^
	CCL22/CCR4	Prostate cancer	Promoting migration and invasion via phosphorylation of Akt	^230^
	CCL22/CCR4	Colorectal cancer	Enhancing chemoresistance via PI3K/AKT pathway and caspase‐mediated apoptosis	^231^
	CCL22/CCR4	Liver cancer	Recruiting regulatory T cells through p65/miR‐23a/CCL22 axis	^232^
	CXCL1/CXCR2	Lung cancer	Recruiting Treg cells via miR141‐CXCL1‐CXCR2 signaling	^92^
	CXCL1/CXCR2	Breast cancer	Promoting metastasis via activating NF‐κB/SOX4 signaling; promoting cancer cell survival mediated by TNF‐αvia NF‐κB signaling; promoting metastasis and chemoresistance through myeloid cell‐derived S100A8/9; stimulating migration and invasion via activation of the ERK/MMP2/9 signaling	^105^, ^233^, ^234^, ^235^
	CXCL1/CXCR2	Pancreatic cancer	Promoting migration and invasion by CXCL1‐mediated Akt phosphorylation	^236^
	CXCL1/CXCR2	Ovarian cancer	Recruiting MDSC mediated by Snail; Enhancing metastatic potential via the TAK1/NF‐κB signaling; driving cancer progression by NF‐κB activation via EGFR‐transactivated Akt signaling	^237^, ^238^, ^239^
	CXCL1/CXCR2	Prostate cancer	Promoting migration via the Src activation; increasing migration and invasion by fibulin‐1 downregulation through NF‐κB/HDAC1	^240^, ^241^
	CXCL1/CXCR2	Colorectal cancer	Forming a premetastatic niche stimulated by VEGFA	^93^
	CXCL1/CXCR2	Gastric cancer	Promoting migration and metastasis through activation of CXCR2/STAT3 signaling; promoting lymph node metastasis through integrin β1/FAK/AKT signaling; promoting tumor growth through VEGF pathway activation	^242^, ^243^, ^244^
	CXCL12/CXCR4	Colorectal cancer	Promoting proliferation, invasion, angiogenesis via MAPK/PI3K/AP‐1 signaling; enhancing metastatic potential via PI3K/Akt/mTOR pathway; promoting progression by lncRNA XIST/ miR‐133a‐3p/ RhoA signaling; promoting growth and metastasis through activation of the Wnt/β‐catenin pathway; promoting chemoresistance via surviving	^245^, ^246^, ^247^, ^248^, ^249^
	CXCL12/CXCR4	Breast cancer	Promoting metastasis by Pit‐1‐CXCL12‐CXCR4 axis; driving the metastatic phenotype through activation of MEK/PI3K pathway	^250^, ^251^
	CXCL12/CXCR4	Ovarian cancer	Recruiting MDSCs mediated by PGE2; promoting invasion through suppressing ARHGAP10 expression; promoting growth and migration through Notch pathway	^94^, ^252^, ^253^
	CXCL12/CXCR4	Lung cancer	Suppressing cisplatin‐induced apoptosis through JAK2/STAT3 signaling	^254^
	CXCL12/CXCR4	Prostate cancer	Inducing myofibroblast phenoconversion through EGFR/MEK/ERK signaling; promoting migration and invasion through SLUG	^255^, ^256^
	CXCL12/CXCR4	Gastric cancer	Inducing migration via SRC‐mediated CXCR4‐EGFR; increasing invasiveness via integrin β1 clustering; promoting migration by F‐actin reorganization and RhoA activation through mTOR signaling	^257^, ^258^, ^259^
	CXCL12/CXCR4	Thyroid papillary cancer	Enhancing proliferation and invasion through Akt and snail signaling	^260^
	CXCL8/ CXCR1/2	Gastric cancer	Inhibiting CD8+ T cells function by inducing the expression of PD‐L1	^261^
	CXCL8/ CXCR2	Lung cancer	Stimulating cell proliferation via transactivation of the EGFR involving the MAPK pathways	^262^
	CXCL8/ CXCR1/2	Breast cancer	Increases the activity of cancer stem‐like cells by transactivation of HER2	^263^
	CXCL8/ CXCR1/2	Melanoma	Increasing tumor growth and metastasis by activating MMP‐2	^264^
	CXCL8/ CXCR1/2	Prostate cancer	Promoted tumorigenesis via STAT3/MALAT1 pathway	^265^
	CXCL8/ CXCR1/2	Colorectal cancer	Enhancing resistance of cancer cells to anoikis through AKT/TOPK/ERK signaling; enhancing migration by increasing αvβ6 integrin expression; promoting proliferation and migration through heparin binding EGF	^266^, ^267^, ^268^
	CX3CL1/CX3CR1	Breast cancer	Promoting the migration and invasion via Src/FAK signaling; triggering proliferation through transactivation of EGF pathway	^269^, ^270^
	CX3CL1/CX3CR1	Lung cancer	Promoting the migration and invasion via Src/FAK signaling; promoting lymph node metastasis via JNK and MMP2/MMP9 pathway	^271^, ^272^
	CX3CL1/CX3CR1	Ovarian cancer	Promoting EMT mediated by HIF‐1α	^273^
	CX3CL1/CX3CR1	Prostate cancer	Promoting EMT via TACE/TGF‐α/EGFR pathway and upregulation of Slug; promoting metastasis via EGFR through activation of the Src/FAK pathway	^274^, ^275^
	CX3CL1/CX3CR1	Pancreatic cancer	Enhancing growth and migration through JAK/STAT signaling; promoting motility, invasion, and growth via AKT activation; promoting tumor cell survival and TRAIL resistance via RelA/NF‐κB signaling; stimulating HIF‐1α expression through the PI3K/Akt and MAPK pathways	^276^, ^277^, ^278^, ^279^
Tyrosine kinase receptor signaling	CSF‐1/CSF‐1R	Gliomas	Promoting tumor cell survival through IGF‐1R/ PI3K signaling	^280^
	CSF‐1/CSF‐1R	Breast cancer	Driving tumor progression through Kindlin‐2/TGFβ/CSF‐1 signaling	^281^
	CSF‐1/CSF‐1R	Ovarian cancer	Promoting metastasis through the induction of MMP‐9 activity	^282^
	CSF‐1/CSF‐1R	Pancreatic cancer	Remodeling tumor immune microenvironment via PI3K‐γ and CSF‐1/CSF‐1R pathways	^283^
	Ron	Prostate cancer	Promoting prostate cancer cell growth via MST1R	^284^
	Ron	Pancreatic cancer	Promoting macrophage polarization through MST1‐MST1R signaling	^285^
Metabolic signaling	Glycolysis	Pancreatic cancer	Conferring a prometastatic phenotype by HK2;	^286^
	Glycolysis	Breast cancer	Promoting M2‐TAMs polarization through activation of GPR132 by lactate; promoting tumor angiogenesis by hypoxia‐induced REDD1/ mTOR signaling	^287^, ^288^
	Glycolysis	Melanoma	Promoting M2‐TAMs accumulation by HMGB1; inducing TME acidification involving GPCR	^141^, ^289^
	Glycolysis	Thyroid carcinoma	Inducing reprogramming of TAMs and inflammation through AKT1/mTOR pathway	^140^
	Glycolysis	Lung cancer	Increasing glucose uptake and glycolysis flux by activation of AMPK; promoting M2‐TAMs polarization mediated by HIF1α	^290^, ^291^
	Fatty acid metabolism	Bladder carcinoma	Promoting cancer growth and metastasis through COX‐2/PGE2 pathway	^292^
	Fatty acid metabolism	Liver cancer	Promoting cell migration by enhancing IL‐1β secretion; enhancing the accumulation and polarization of M2‐like TAMs by RIPK3	^293^, ^294^
	Fatty acid metabolism	Breast cancer	Promoting cancer progression by FABP by favoring IL6 / STAT3 signaling; promoting TAMs differentiation through caspase‐1/PPARγ/MCAD pathway	^295^, ^296^
	Fatty acid metabolism	Ovarian cancer	Promoting the M2‐like polarization through the PPARγ/NF‐κB pathway	^297^
	Glutamine synthesis	Lung cancer	Promoting M2‐like polarization through GS	^298^
	Tryptophan metabolism	Colon, gastrointestinal cancer	Inhibiting T‐cell functions through IDO upregulation	^299^, ^300^
	Arginine metabolism	Melanoma	Promoting tumor cell proliferation through ARG1	^301^
	Arginine metabolism	Breast cancer	Promoting cell growth by ARG induction and suppressing NO‐mediated tumor cytotoxicity	^302^
	Iron metabolism	Breast cancer	Releasing iron mediated by FPN in the TME; supporting proliferation by LCN	^303^, ^304^
	RAGE	Breast cancer	Enhancing tumor growth and metastasis through S100A7 signaling	^305^
	RAGE	Glioma	Promoting angiogenesis through MMP9 signaling	^306^
	RAGE	Melanoma	Promoting invasiveness through S100A4 signaling	^307^
Exosomal signaling	miR‐21‐5‐p, miR‐155‐5p	Colorectal cancer	Promoting migration and invasion by downregulating expression of BRG1	^164^
	miR‐25‐3p, miR‐130b‐3p, miR‐425‐5p	Colorectal cancer	Inducing M2‐like macrophage polarization by regulating PTEN/PI3K/Akt signaling	^308^
	Wnt	Colorectal cancer	Promoting chemoresistance through Wnt signaling	^309^
	miR‐125b‐5p	Melanoma	Educating TAMs by targeting LIPA	^310^
	miR‐21	Gastric cancer	Suppressing apoptosis through PI3K/AKT signaling by down‐regulation of PTEN	^57^
	miR‐301a	Pancreatic cancer	Inducing M2‐like macrophage polarization via PTEN/PI3Kγ signaling	^311^
	miRNA‐501‐3p	Pancreatic cancer	Promoting progression through the TGFBR3‐mediated TGF‐β signaling	^312^
	miR‐223	Ovarian cancer	Promoting drug resistance via the PTEN‐PI3K/AKT pathway	^313^
	miR‐95	Prostate cancer	Promote cell proliferation, invasion, and EMT via miR‐95/JunB axis	^314^

### 4.1 Chemokine–chemokine receptor signaling

**FIGURE 2:**
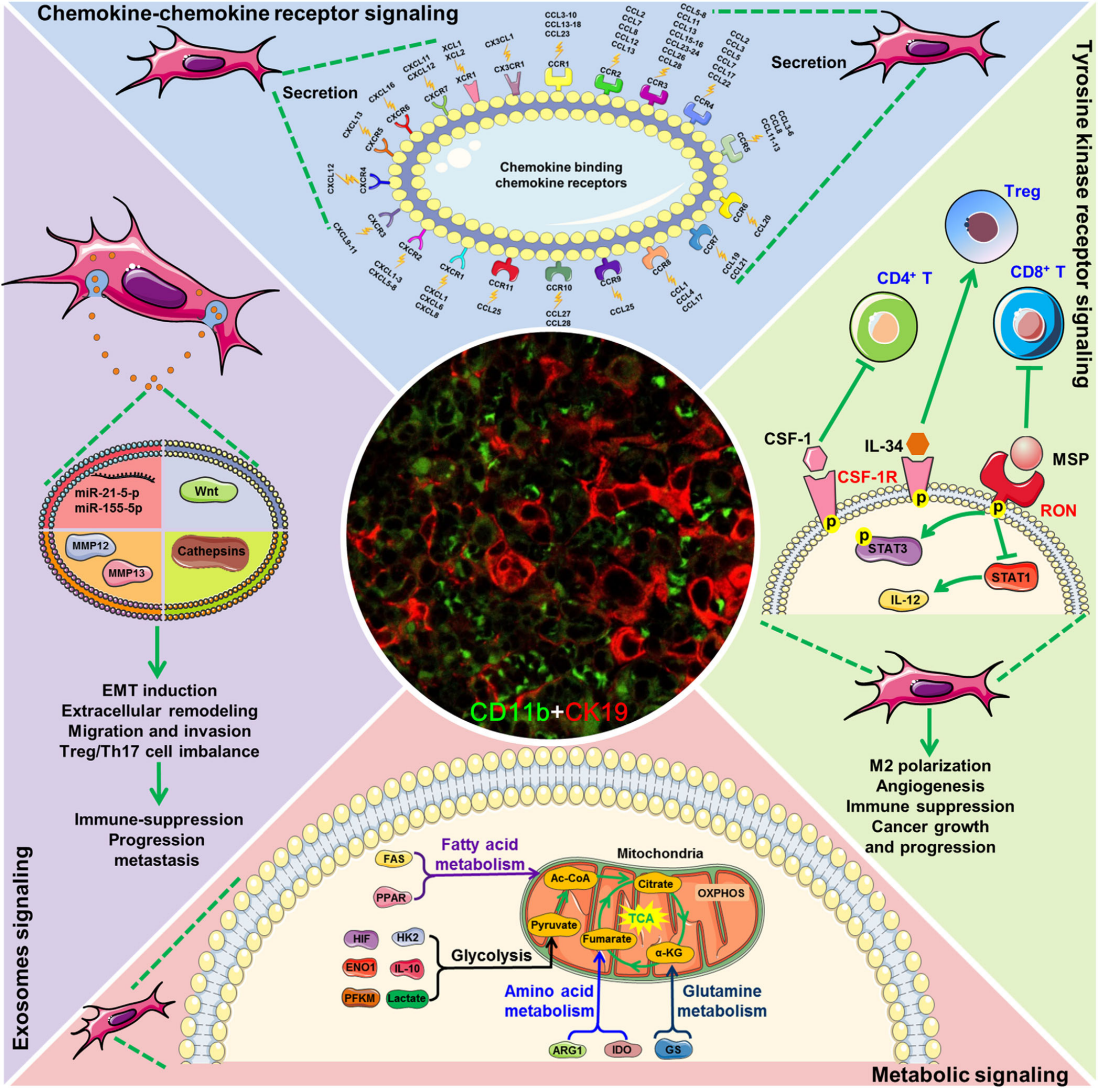
Pharmaceutical targets involved in TAMs regulation. Chemokine–chemokine receptor signaling was the most important target for TAMs‐targeting therapy, particularly for CCL2‐CCR2, CCL5‐CCR5, CXCL1/CXCR2, and CXCL12/CXCR4 signaling. Tyrosine kinase receptors including CSF‐1R and RON were also highly expressed on the cell membrane of TAMs and perfect for drug intervention. Molecular targets involved in glycolysis, fatty acid oxidation, and amino acid metabolism in TAMs were also being focused on drug development. Notably, exosomes act as the ideal drug‐loading and delivery vehicle for modulating biofunctions of TAMs

Chemokines, which are small and soluble (8–14 kDa) signaling proteins, are a family of chemotactic cytokines responsible for cellular trafficking. More than 50 kinds of chemokines and 20 kinds of chemokine receptors have been identified until now.^79^ According to the positions of conserved cysteine residues, chemokines can be classified into four groups including CXC, CC, CX3C, and C.^80^ Correspondingly, chemokine receptor nomenclature essentially follows that of chemokines. Eleven kinds of CC chemokine receptors (CCR1–CCR11) recognize the CC subfamily chemokines, while seven kinds of CXC receptors (CXCR1–CXCR7) recognize the CXC subfamily chemokines, respectively.^80^ Similarly, the CX3C subfamily chemokine (CX3CL1) only binds to CX3CR1 receptor while the C subfamily chemokines (XCL1/2) only bind to XCR1 receptor, respectively. Chemokine receptors are typical G‐protein‐coupled receptors (GPCRs) with seven transmembrane domains. Chemokine receptors can relay their signals through heterotrimeric G proteins that result in the directional migration of cells along a concentration gradient of ligands.^79^ The interactions between chemokine and chemokine receptors are summarized in Figure 2 and Table 1. In the TME, TAMs are considered as the main stromal cells that secrete chemokines. As indicated by chemokine arrays, highly expressed chemokines of TAMs have been reported to consist of CCL2, CCL3, CCL5, CCL18, CXCL1, and CXCL12. Currently, the ligand receptor for CCL2 has been identified as CCR2. CCR1 and CCR5 have been found to be the receptors for both CCL3 and CCL5. Meanwhile, CCR3 has also been identified as a receptor for CCL5. By immunoprecipitation methods, the cognate receptor for CCL18 has been identified as PITPNM3, which has been suggested to be a putative six‐transmembrane protein that is sufficient to exert GPCR‐related functions. Additionally, the receptors for CXCL1 and CXCL12 have been identified to be CXCR2 and CXCR4, respectively.

Chemokines and chemokine receptors are important for attracting infiltrating leukocytes to the TME. CCL2 and CCL5 are the primary chemokines responsible for monocytic precursors recruitment in tumors.^81^, ^82^, ^83^ Their expression levels are closely associated with TAMs infiltration, lymph‐node metastasis, as well as unfavorable prognosis. CCL2 production from macrophages is critical for recruiting myeloid‐derived suppressor cells (MDSCs) to build a local immunosuppressive microenvironment in gliomas.^84^ Moreover, in mixed‐bone marrow chimeric assays, CCR2‐deficient MDSCs failed to accumulate in gliomas.^84^ In addition, the CCL2‐CCR2 axis was also found to be critical in mobilizing dendritic cell‐like antigen‐presenting cells into fibrosarcomas, as well as tumor‐infiltrating lymphocytes.^85^ Multiple studies have demonstrated that CCL5 can inhibit the antitumor immune response and is correlated with poor outcomes in multiple malignancies.^86^, ^87^, ^88^, ^89^ CCL5/CCR3 signaling has been found to promote Th2 immune polarization and results in luminal breast cancer.^90^ The CCL5/CCR5 chemokine axis is also capable of enhancing TGF‐β‐mediated killing of cytotoxic CD8^+^ T cells in colon cancer through regulatory T cells.^91^ With regard to CXC chemokines, CXCL1 chemokines have been demonstrated to be effective in recruiting CXCR2^+^ Treg cells and lead to metastasis in nonsmall cell lung cancer.^92^ CXCL1 is also critical in facilitating PMN formation in colorectal cancer by recruiting CXCR2^+^ MDSCs.^93^ Meanwhile, CXCL12/CXCR4 signaling has been reported to induce the recruitment of MDSCs in the TME of human ovarian cancer.^94^ Besides shaping the tumor immune milieu, chemokine axis is also critical in regulating the neoangiogenic process. CXC chemokines have been reported to be directly chemotactic for endothelial cells and to stimulate angiogenesis *in vivo*.^95^ CXCR4 receptor is expressed on endothelial cells and its activation after CXCL12 binding can induce the proliferation and migration of endothelial cells. Moreover, CXCL12 recruits CXCR4^+^ circulating or bone‐marrow endothelial precursors and therefore promotes tumor angiogenesis.^96^ Similarly, CXCL1 was also reported to promote neoangiogenesis in breast, liver, and colorectal cancers.^97^, ^98^, ^99^ Finally, chemokine signaling can directly act on chemokine receptors expressed on the plasma membrane of cancer cells, thus controlling their malignancy‐related functions. For example, a variety of chemokine receptors—including CXCR2, CXCR4, CCR1, CCR2, and CCR5—have been found in breast tumor cells.^100^, ^101^, ^102^, ^103^, ^104^ It is found that CXCL1 can induce chemoresistance and metastasis of breast cancer *via* CXCR2 activation.^105^ Furthermore, CCL2 induces hepatocellular carcinoma invasion and EMT *via* CCR2 activation.^106^ CXCL12 is also capable of regulating glioblastoma‐stem‐like cells by modulating CXCR4.^107^ Moreover, CXCL12‐CXCR4 signaling has also been highlighted in guiding the homing of cancer cells to their specific metastatic organs.^108^ Altogether, these diverse chemokine functions establish crosstalk between cancer cells and the TME, which represents a promising target for the successful development of novel therapeutic strategies.

It should be pointed out that multiple chemokines play multifaceted roles within the complicated TME, usually dividing into two directions: paracrine signaling for immune activation and autocrine signaling for proliferation and metastasis of cancer cells.^109^, ^110^, ^111^ For example, the CXCL9‐10 axis could induce the migration, differentiation, and activation of the CXCR3^+^ activated T or NK cells, leading to increased antitumor immune response.^112^ Additionally, CCL5/CCR5 axis is also a double‐edged sword in cancer. On one hand, CCL5 could promote CSCs self‐renewal and recruit the immunosuppressive cells (such as CCR5^+^ TAMs and Tregs) to form a tumor‐protecting TME. On the other hand, CCL5 could also promote antitumor immunity by recruiting CCR5^+^ activated T cells and DCs to the TME and therefore enhances the immunotherapy response in different tumor types.^110^, ^111^

TAMs‐related chemokine signals have been widely investigated in skin area because of their reliable prognosis and therapeutic values for skin tumors. For example, Wu *et al*. reported that TAMs‐related chemokine signals played key roles in the development and progression of cutaneous T‐cell lymphoma (CTCL). Compared with normal controls, mycosis fungoides (the most common variant of CTCL) skin displayed abundant TAMs infiltration as well as increased expression of a subset of macrophage‐related chemokines and associated receptors.^113^ Selective depletion of M2‐phenotype TAMs using clodronate‐containing liposomes remarkably delayed mycosis fungoides development *in vivo*. Interferons (IFNs) have been widely used for mycosis fungoides and malignant melanoma treatment in the clinic. Growing evidence has suggested that modulating TAMs‐related chemokine signals may be the possible mechanism of the therapeutic effects of IFNs. For example, Furudate *et al*. reported that IFN‐α2a and IFN‐γ stimulation could decrease the release of CCL17/18, whereas elevating the production of CXCL10/11 in monocyte‐derived TAMs *in vitro*. More importantly, the subcutaneous administration of IFN‐α2a could increase the CXCL11‐secreting cell population in the lesional skin of advanced mycosis fungoides patients.^114^ Kakizaki *et al*. reported that peritumoral injection of IFN‐β remarkably suppressed the recruitment of PD‐L1^+^ TAMs in B16F10 melanoma xenograft *in vivo*.^115^ Meanwhile, IFN‐β administration dramatically attenuated the production of CCL17/22, whereas elevated the secretion of CXCL9/11 from TAMs. More importantly, these immunomodulatory effects of IFN‐β on TAMs were also observed in the lesional skin of patients with in‐transit melanoma. It should be noted that the clinical application of IFNs for the treatment of cancer is still controversial. For example, Numerous clinical trials have examined the antitumor potential of recombinant IFN‐γ as an adjuvant to surgery or conventional chemotherapy.^116^ However, these clinical trials often reported conflicting outcomes in patients with the same tumor type.^116^ Extensive efforts are still needed to better understand the complex roles of IFNs in cancer treatment.

### 4.2 RTK signaling

RTKs play a vital role in signal transduction that supports cellular communication and survival. Dysregulation of RTKs, such as receptor mutation or overexpression, contributes to the initiation, tumorigenesis, and progression of multiple malignancies. To date, it has been reported that 58 kinds of RTKs are edited by the human genome.^117^, ^118^ Notably, colony stimulation factor‐1 receptor (CSF‐1R), one of the most important members of the RTKs family, is highly expressed by TAMs.^119^ Upon binding to its ligands including CSF‐1 or IL‐34, CSF‐1R undergoes dimerization and therefore causes a signaling cascade that facilitates the proliferation, functioning, and survival of macrophages. High expression of CSF‐1 has been found in breast, prostate, pancreatic, gastric, and many other types of cancers, and its level has been closely associated with TAMs density and poor prognosis.^120^, ^121^, ^122^, ^123^ It is found that CSF‐1‐positive cancer cells colocalize with abundant CSF‐1R‐positive TAMs in invasive breast cancer, and the CSF‐1/CSF‐1R axis is significantly correlated with a higher grade, basal‐like phenotype, and lymph node metastasis.^124^ In CSF‐1‐knockout MMTV‐PyMT^+/‐^ spontaneous breast cancer mice, the perivascular macrophage density was declined with a sixfold reduction, accompanied by a 16‐fold decrease of circulating tumor cells in the blood.^125^ Moreover, CSF‐1/CSF‐1R signaling of macrophages could suppress CD4^+^ T‐cell responses and induce the generation of Treg cells, which facilitated the immune‐suppressive TME.^126^ Additionally, CSF‐1 is also involved in the pro‐angiogenic process since it promotes M2‐like polarization of macrophages and secretes a variety of angiogenic factors.^127^ Therefore, blocking CSF‐1/CSF‐1R activation is becoming an important therapeutic strategy to inhibit cancer growth and metastasis.

RON is another RTK expressed on macrophages surface that specifically recognizes macrophage‐stimulating protein (MSP). Overexpression of RON has been implicated in the progression and metastasis of diverse malignancies. In breast cancer, MSP/RON overactivation is sufficient to promote cancer growth as well as metastasis to the lungs, liver, brain, and bone. Conversely, RON inactivation is capable of inhibiting the growth, angiogenesis, and metastasis of breast cancer xenografts.^128^ The cancer‐promoting effects of MSP/RON signaling are mainly attributed to the M2‐like phenotype polarization of macrophages. RON activation can cause the phosphorylation of STAT3, which is essential for the tumor‐promoting efficacies and immunosuppressive functions of TAMs.^129^ By contrast, the MSP/RON pathway can downregulate STAT1 activity, which actively participates in antitumor immune responses *via* up‐regulating IL‐12.^130^ Eyob *et al*. has demonstrated that RON signaling in macrophages is capable of impairing antitumor functions of CD8^+^ T cells.^131^ Altogether, both STAT3 activation and STAT1 inactivation induced by MSP/RON signaling may contribute to tumor immune tolerance and lead to cancer progression. Considering the important role of RON receptor signaling in TAMs modulation, pharmacological inhibition of RON kinases has received more attention from research groups around the world.

Mer tyrosine kinase (MerTK) is also a macrophage‐specific RTK and its overexpression usually correlates with poor prognosis in cancer.^132^ MerTK signaling activation can promote tumor immune tolerance by initiating macrophages efferocytosis and promoting the clearance of immunogenic antigens. Meanwhile, MerTK signaling activation can suppress inflammatory cytokines production and polarize macrophages toward the anti‐inflammatory M2‐like phenotype.^133^ In contrast, macrophages MerTK signaling blockage can induce the repolarization of macrophages toward the M1‐like phenotype and enhance antitumor immunogenic cell death, a crucial step in T‐cell priming and activation.^134^ Thus, MerTK blockade may represent a promising antitumor strategy that could significantly increase tumor immunogenicity and potentiate antitumor immunity. It has been reported that MerTK knockdown or MerTK neutralizing antibody treatment could remodel the cellular immune profile, bringing a more inflamed TME with increased T cell infiltration and T cell‐mediated cytotoxicity.^132^ Additionally, anti‐MerTK antibody treatment could stimulate T cell activation and synergize with anti‐PD‐1 or anti‐PD‐L1 therapies in tumor‐bearing mice.^135^ Altogether, macrophages MerTK blockade could promote tumor immunogenicity and potentiate antitumor immunity, which represents a promising strategy for tumor immunotherapy.

### 4.3 Metabolic signaling

A typical feature of cancer cells is their abnormal metabolism caused by oxygen deprivation and nutrient limitation.^136^ Hypoxic TME not only induces the metabolism pattern shifting of cancer cells but also reprograms the polarization balance and metabolic mode of macrophages, which facilitate the formation of immune‐suppressive TME. In hypoxic regions of tumors, a more dominant M2‐like phenotype is observed, which might indicate the potential effects of hypoxia on macrophage polarization.^137^ Hypoxia‐inducible factors (HIFs) are major transcriptional factors that are responsive to hypoxia and control aerobic glycolysis.^138^ Enhanced glycolytic activity allows macrophages to synthesize sufficient ATP and various biosynthetic intermediates to maintain their tumor‐prone functions. A recent study demonstrated the enhanced glycolytic activity of TAMs when compared with that of bone marrow–derived macrophages (BMDMs). Glycolytic proteins, such as hexokinase‐2, phosphofructokinase, and enolase 1, were all increased in TAMs.^139^ Similar findings were also observed in a transwell co‐culture model of macrophages with cancer cells or tumor‐extract solutions.^140^ In addition to intrinsic HIF activation, cytokines and lactate released from cancer cells under hypoxic conditions also contribute to the glycolytic phenotype of TAMs. In hypoxic melanoma cells, HIF‐1α accumulation induces HMGB1 translocation that causes IL‐10 production, which consequently promotes M2‐like macrophage activation.^141^ Cancer‐cell–derived lactate has also been reported to reprogram macrophages to a tumor‐prone phenotype by inhibiting the NF‐κB pathway that in turn may impair M1‐like activation and suppress the immune surveillance functions of infiltrating T cells and NK cells.^142^, ^143^, ^144^ Interestingly, the OXPHOS chain has also been found to be elevated in a coculture model of thyroid‐carcinoma cells and macrophages, characterized by enhanced oxygen consumption rate.^140^ The concentrations of AcCoA and succinate are significantly increased in the mitochondria of M2‐like macrophages, suggesting that TAMs have an intact OXPHOS despite an impaired TCA cycle.^145^ The enhanced OXPHOS chain may not only yield ROS‐induced DNA damage but may also provide citrates for fatty acid synthesis. However, more studies are still required to reveal the precise switching mechanisms between glycolysis and OXPHOS in TAMs depending on cancer types and stages.

Fatty acid metabolism is important in cancer development. It has been suggested that increased fatty acid metabolism provides cancer cells with sufficient membranes and energy to support cancer growth.^146^ It is found that fatty acid oxidation is necessary for IL‐4‐induced macrophage activation, as CPT1 suppression could attenuate the expression of the M2‐like marker proteins, CD301, and CD206.^147^ Meanwhile, fatty acid oxidation can activate the downstream COX2/PGE2 pathway to promote cancer growth and metastasis. TAMs express high levels of fatty acid synthase and increased PPAR signaling, which promote fatty acid oxidation and tumor growth.^148^ PPARs are composed of three isoforms including PPAR‐α, PPAR‐β/δ, and PPAR‐γ. PPAR‐α manages lipid oxidation and clearance. PPAR‐γ facilitates lipogenesis while PPAR‐β/δ controls lipid uptake and storage.^149^ PPAR‐β/δ plays a crucial role in mediating M2‐like polarization.^150^ Ovarian cancer patient‐derived TAMs exhibited increased expression levels of multiple PPAR‐β/δ target genes when compared with that of monocyte‐derived macrophages.^151^ Lipidomic analysis revealed that the deregulation of PPAR‐β/δ target genes did not result from PPAR‐β/δ overexpression or its promotion effect on target genes, but was attributed to high levels of polyunsaturated fatty acids, especially arachidonic acid and linoleic acid in tumor ascites.^151^ The internalization of polyunsaturated fatty acids and the subsequent lipid droplet formation may provide a reservoir of PPAR‐β/δ ligands to TAMs, leading to a consistent activation of PPAR‐β/δ target genes, such as pyruvate dehydrogenase kinase 4 (PDK4). Interestingly, PDK4 can induce glucose catabolism shifting toward aerobic glycolysis, further rendering TAMs to adapt to hypoxic TME.^151^ PPAR‐γ is also known to regulate metabolic pathways and M2‐like polarization. Macrophage deficiencies in PPAR‐γ and δ and their coactivator, PGC1β, are ineffective for IL‐4‐induced fatty acid oxidation and M2‐like polarization.^152^ Hence, targeting of PPAR signaling is a promising approach to control macrophage activation and cancer progression.

In addition to enhanced glycolysis and fatty acid oxidation, M2‐like macrophages have also been found to increase glutamine synthesis through glutamate, which has not been demonstrated in M1‐like macrophages.^153^ Inhibition of glutamine syntheses results in a 50% reduction of M2‐like polarization, which might be ascribed to the role of glutamine in protein glycosylation.^154^ Meanwhile, glutamine is extensively fluxed into the TCA cycle to provide enough carbons to satisfy energetic needs.^155^ Besides glutamate, another amino acid, arginine, is also required in the ARG1 pathway of M2‐like macrophages.^156^ Interestingly, ARG1 is also the downstream gene of HIF‐1α. ARG1 overexpression not only enables cancer cells to rapidly adapt to the hypoxic microenvironment but also results in the production of ornithine and urea.^156^ Ornithine can be further utilized in the synthesis of proline and polyamine, which are required for cellular proliferation, tissue remodeling, and collagen biogenesis.^157^ Moreover, the arginine clearance by ARG1 suppresses T‐cell immune responses, favoring the establishment of the immunosuppression microenvironment.^157^ Besides, tryptophan metabolism has been shown to be deregulated in cancers. It was found that the enzyme accounting for tryptophan catabolism, indoleamine‐2, 3‐dioxygenase (IDO), was frequently overexpressed in M2‐like macrophages.^158^ IDO upregulation was reported to inhibit T‐cell functions, whereas tryptophan treatment or administration of IDO inhibitors could restore T‐cell proliferation.^158^

RAGE (receptor for advanced glycation end‐product) is a member of the immunoglobulin superfamily and has been reported to be highly expressed on the surface of macrophages.^159^ It has been reported that RAGE overexpression could induce the accumulation of TAMs and thus accelerate the growth of lung cancer xenografts *in vivo*.^160^ Mechanistically, RAGE expression strictly correlates to the metabolic switch in cancer and immune cells. For example, cancer cells usually exhibit a metabolism switch characterized by decreased mitochondrial oxidative phosphorylation as well as elevated glycolysis, leading to the overproduction of toxic glucose metabolites.^161^ The toxic glucose metabolites such as lactate, sorbitol, diacylglycerol (DAG), and methylglyoxal (MG) could further contribute to the development of advanced glycation end products (AGEs). On one hand, the formation of AGEs could impair the phagocytosis function of M1‐like macrophages within the TME.^162^ On the other hand, AGEs could induce RAGE expression in macrophages and their interaction could further promote cancer progression and metastasis by recruiting TAMs.^160^, ^163^ Therefore, AGEs/RAGE signaling may also represent a metabolism‐related target for the pharmacological modulation of TAMs, which still needs further investigations. Collectively, the above findings suggest that the metabolic pathways and targets can also be applied to modulate the biological functions of TAMs, which may provide valuable opportunities for treating multiple malignancies (Figure 2 and Table 1).

### 4.4 Exosomal signaling

In recent years, exosomal signaling has evoked increased interest in cancer research. Exosomes are defined as small extracellular vesicles that are 30–100 nm in diameter.^164^ Similar to cells, exosomes are composed of a lipid bilayer containing all known molecular components including DNA, RNA, and proteins.^165^ Exosomes can be released by all cell types and widely exist in human bodily fluids such as blood, urine, amniotic fluid, bronchoalveolar lavage fluid, and malignant fluid. In particular, normal human blood is expected to contain approximately 2,000 trillion exosomes, while this value is elevated to 4,000 trillion in the blood of cancer patients.^166^, ^167^, ^168^ Therefore, exosomal signaling is highly implicated in intercellular communication and carcinogenesis. Recent studies have also demonstrated that exosomal signaling plays a vital role in tumor angiogenesis,^169^ metastasis,^170^ drug resistance,^171^ and immune regulation,^172^ particularly for exosomes derived from macrophages. It has been reported that TAMs‐derived exosomes exhibit high expression levels of miR‐21‐5‐p and miR‐155‐5p, which significantly promote cellular migration and invasion of colorectal cancer cells.^164^ Besides, exosomes from macrophages also contain a high level of Wnt, which is considered to provide critical signaling in EMT induction and mesenchymal‐phenotype maintenance.^173^, ^174^ In addition to metastasis, TAMs‐derived exosomes also contribute to extracellular‐matrix remodeling. It has been shown that MMP‐12 and MMP‐13, as well as cathepsin B, D, K, L, S, and Z, are all upregulated in TAMs‐derived exosomes, suggesting their positive roles in extracellular remodeling.^175^ Notably, exosomes released from TAMs also contain miRNAs that lead to Treg/Th17 cell imbalance in ovarian cancer, resulting in the generation of an immune‐suppressive TME and thus promoting cancer progression and metastasis.^176^ Altogether, targeting TAMs‐derived exosomal signaling represents a novel and promising approach for cancer treatment (Table 1).

## 5 PHARMACOLOGICAL MODULATION OF TAMS

### 5.1 Agents targeting chemokine–chemokine receptors

Chemokine–chemokine receptor signaling constitutes an important network that regulates cancer growth, angiogenesis, immune suppression, and metastasis. Targeting of chemokine–chemokine receptor networks has been evaluated in multiple preclinical models and clinical trials, particularly in terms of the CCL2‐CCR2, CCL5‐CCR5, and CXCL12‐CXCR4 axes (Table 2). Blockade of CCL2 with a neutralizing antibody inhibited inflammatory monocyte recruitment, reduced lung metastasis, and prolonged OS in breast cancer–bearing mice.^315^ In a metastatic prostate cancer model, combined treatment with an anti‐CCL2 antibody and docetaxel caused reduced tumor burden and bone resorption, as well as an improved survival period.^316^ The development of CCR2 inhibitors has also yielded encouraging results in cancer treatments. Oral administration of the CCR2 inhibitor, PF‐04136309, in a preclinical pancreatic cancer model reduced the number of tumor‐infiltrating macrophages and inflammatory monocytes.^317^ Significantly, PF‐04136309 acted synergistically with the chemotherapeutic drug, gemcitabine, to inhibit metastasis, increase anti‐tumor T‐cell responses, and ultimately lead to an enhanced chemotherapeutic efficacy.^318^ Similarly, the synergistic effects between PF‐04136309 and paclitaxel or the standard chemotherapy, FOLFIRINOX, have also been reported in pancreatic cancer patients.^319^ Another CCR2 inhibitor, CCX872, was demonstrated to improve anti‐PD‐1 treatment in preclinical settings and positive results were also obtained when used in combination with the FOLFIRINOX strategy in pancreatic cancer patients.^320^ In murine models of hepatocellular carcinoma, combined treatment with the CCR2 antagonists, RDC018 or 747, with sorafenib was validated to inhibit tumor growth and metastasis, accompanied by a significant reduction of macrophage infiltration.^321^, ^322^ It should be pointed out that there are also some disappointing results in the clinical trials of CCL2/CCR2 blockers. For example, carlumab, a human IgG1κ anti‐CCL2 mAb, did not show antitumor activity as a single agent in metastatic castration‐resistant prostate cancer patients in a phase II study.^323^ Overall, the above results suggest that the CCL2‐CCR2 axis may be a promising target for cancer treatment, especially when used in combination with traditional cancer‐killing strategies or immunotherapies. However, the successful pharmaceutical development of CCL2/CCR2 blockers still has a long way to go.

**TABLE 2:** Chemokine–chemokine receptor targeting agents in clinical trials

Drug/description	Sponsors	Phase/status	Tumor type	Treatment	ClinicalTrials.govIdentifier	First received
CCL2‐CCR2 inhibitor						
Carlumab/CNTO 888 (anti‐CCL2 antibody)	Centocor Research & Development	2^a^	Prostate cancer	Monotherapy	NCT00992186	2009‐10‐09
MLN1202/S0916 (anti‐CCR2 antibody)	Southwest Oncology Group	2^a^	Metastatic cancer	Monotherapy	NCT01015560	2009‐11‐08
Navarixin/MK‐7123/ SCH 527123(CXCR1 and CCR2 antagonist)	Merck Sharp & Dohme	2^b^	Advanced/Metastatic solid tumors	With pembrolizumab	NCT03473925	2018‐03‐22
PF‐04136309/PF6309 (CCR2 antagonist)	Washington University School of Medicine and National Cancer Institute	1b^a^	Locally advanced or borderline resectable pancreatic cancer	With FOLFIRINOX chemotherapy	NCT01413022^319^	2011‐08‐09
CCX872‐B (CCR2 antagonist)	ChemoCentryx	1b^b^	Pancreatic cancer	With FOLFIRINOX chemotherapy	NCT02345408	2015‐01‐26
AZD5069 (CCR2 antagonist)	Institute of Cancer Research, UK	1/2^b^	Metastatic castration‐resistant prostate cancer	With enzalutamide	NCT03177187	2017‐06‐06
Reparixin (CXCR1 and CCR2 antagonist)	Dompé Farmaceutici S.p.A	1^a^	Metastatic breast cancer	With paclitaxel	NCT02001974^354^	2013‐12‐05
SX‐682(CXCR1 and CCR2 antagonist)	Syntrix Biosystems	1^b^	Metastatic melanoma	With pembrolizumab	NCT03161431	2017‐05‐19
CCL5‐CCR5 inhibitor						
Maraviroc/Celsentri (CCR5 antagonist)	Abramson Cancer Center of the University of Pennsylvania	2^a^	Hematologic malignancy	Monotherapy	NCT01785810	2013‐02‐07
	University Hospital Heidelberg	1^a^	Metastatic colorectal cancer	With Pembrolizumab	NCT03274804	2017‐09‐07
	National Center for Tumor Diseases, Heidelberg	1^a^	Colorectal cancer patients with liver metastases	Monotherapy	NCT01736813	2012‐11‐29
Leronlimab/PRO‐140 (anti‐CCR5 antibody)	CytoDyn	1b/2^b^	Triple negative breast neoplasms	With carboplatin	NCT03838367	2019‐02‐12
CXCL12‐CXCR4 inhibitor						
Mogamulizumab/ KW‐0761 (anti‐CCR4 antibody)	Kyowa Kirin	3^b^	Cutaneous T‐cell lymphoma	Monotherapy	NCT01728805^355^	2012‐11‐20
	European Organisation for Research and Treatment of Cancer	2^b^	Stage IB‐IIB cutaneous T‐cell lymphoma	With radiation (total skin electron beam therapy)	NCT04128072	2019‐10‐16
	Kyowa Kirin	2^a^	Adult T‐cell leukemia‐lymphoma	Monotherapy	NCT00920790^356^	2009‐06‐15
	Kyowa Kirin	2^a^	Peripheral T/NK‐cell lymphoma	Monotherapy	NCT01192984	2010‐09‐01
	Kyowa Kirin	1/2^a^	Advanced solid tumors	With nivolumab	NCT02705105	2016‐03‐10
	National Cancer Institute	1/2^b^	Diffuse large B cell lymphoma	With pembrolizumab	NCT03309878	2017‐10‐16
	Kyowa Kirin	1^a^	Non‐small cell lung cancer	With docetaxel	NCT02358473	2015‐02‐09
	Kyowa Kirin	1^a^	Adult T‐cell leukemia‐lymphoma and peripheral T‐cell lymphoma	Monotherapy	NCT00355472^357^	2006‐07‐24
Ulocuplumab/BMS‐936564/MDX‐1338 (Anti‐CCR4 antibody)	Bristol‐Myers Squibb	1^a^	Acute myelogenous leukemia and selected B‐cell cancers	Monotherapy	NCT01120457	2010‐05‐11
	Bristol‐Myers Squibb	1^a^	Multiple myeloma	With Revlimid/ Velcade and dexamethasone	NCT01359657	2011‐05‐25
AMD3100/Plerixafor (CXCR4 antagonist)	Massachusetts General Hospital	2^b^	Head and neck cancer	With pembrolizumab	NCT04058145	2019‐08‐15
	Washington University School of Medicine	2^a^	Hematologic neoplasms	With leukopheresis	NCT00914849^358^	2009‐06‐05
	Washington University School of Medicine	1/2^a^	Acute myeloid leukemia	With mitoxantrone, etoposide, and cytarabine	NCT00512252^359^	2007‐08‐07
LY2510924 (CXCR4 antagonist)	M.D. Anderson Cancer Center	1b^a^	Relapsed or refractory acute myeloid leukemia	With idarubicin and cytarabine	NCT02652871	2016‐01‐12
	Eli Lilly and Company	2^a^	Extensive stage small cell lung carcinoma	With carboplatin and etoposide	NCT01439568^360^	2011‐09‐23
Motixafortide/BKT140/BL‐8040 (CXCR4 antagonist)	Biokine Therapeutics	1/2a^a^	Multiple myeloma	Monotherapy	NCT01010880	2009‐11‐10
	M.D. Anderson Cancer Center	2b^b^	Metastatic and recurrent pancreatic adenocarcinoma	With pembrolizumab	NCT02907099	2016‐09‐20
PTX‐9908 (CXCR4 antagonist)	TCM Biotech International Corp.	1/2^b^	Hepatocellular carcinoma	Monotherapy	NCT03812874	2019‐01‐23
USL‐311 (CXCR4 antagonist)	Proximagen	1/2^b^	Solid tumors and relapsed/recurrent GBM	Monotherapy or with lomustine	NCT02765165	2016‐05‐06
Balixafortide/POL6326 (CXCR4 antagonist)	Polyphor	3^b^	HER2‐ locally recurrent or metastatic breast cancer	With Eribulin	NCT03786094	2018‐12‐24
	Polyphor	2^a^	Multiple myeloma	Monotherapy	NCT01105403	2010‐04‐16
	Polyphor	1^a^	Metastatic breast cancer	With Eribulin	NCT01837095^361^	2013‐04‐22
GMI‐1359 (CXCR4 antagonist)	GlycoMimetics	1b^b^	HR^+^ metastatic breast cancer	Monotherapy	NCT04197999	2019‐12‐13
TG‐0054 (CXCR4 antagonist)	TaiGen Biotechnology	2^a^	Multiple myeloma and non‐Hodgkin lymphoma and Hodgkin disease	With G‐CSF	NCT02104427	2014‐04‐04

*Source*: clinicaltrials.gov.

^a^ Completed stage.

^b^ Ongoing stage, including both recruiting and nonrecruiting stages.

With regard to CCL5‐CCR5 signaling, systemic treatment of mice with CCL5‐directed antibodies inhibit colon cancer growth, lung metastasis, and peritoneal dissemination.^324^ CD45‐immunoreactive cells in tumor tissues and adjacent healthy tissues have been found to be upregulated following CCL5 blockade. Meanwhile, CCL5 neutralization renders colon tumors more sensitive to PDGFRβ‐targeted therapy.^324^ CCL5‐targeted blockade has also been demonstrated to be effective in several malignancies including breast,^325^ gastric,^326^ and prostate cancers.^327^ Concurrently, much attention has been paid to the discovery of CCR5 antagonists. Maraviroc, an FDA‐approved CCR5 antagonist, has been applied as an antiretroviral therapy strategy for HIV infection.^328^ Recent studies have demonstrated its efficacy to suppress cancer cell invasiveness in a variety of cancers. Maraviroc administration resulted in cell‐cycle arrest and apoptosis in both colorectal and breast cancer cell lines *in vitro*.^325^, ^329^ In gastric cancer, treatment with maraviroc effectively reduced tumor burden, inhibited peritoneal dissemination, and prolonged the survival period.^326^ It was also observed that maraviroc significantly inhibited pancreatic cancer liver metastasis, accompanied by marked cell‐cycle arrest at the G1/S checkpoint.^330^ TAK779 is another synthetic CCR5 antagonist that was initially developed for HIV treatments.^331^ Menu *et al*. demonstrated that TAK779 has direct inhibitory effects on melanoma growth *in vitro* and *in vivo*.^332^ Another study suggested that TAK779 could efficiently block CCR‐5‐dependent T‐cell migration.^333^ In a pancreatic cancer mouse model, TAK779 administration resulted in significant suppression of Treg recruitment and cancer growth.^334^ Compared to synthetic CCR5 inhibitors, aibamine is the first natural CCR5 antagonist with an IC_50_ of 1 μM.^335^ Alibamine was found to suppress the invasion and metastasis of prostate cancer cells in mice.^327^ By analyzing the chemical structure differences between anibamine and other CCR5 inhibitors, the main difference has been shown to consist of the side chains of anibamine being simple, undecorated, and aliphatic chains.^336^ Therefore, further drug development based on the structure of anibamine may bring a novel candidate drug for cancer treatment.

CXCL12‐CXCR4 is the most commonly overexpressed signaling pathway in a variety of cancers and a number of small‐molecule drugs and peptide inhibitors that target CXCR4 have been developed. AMD3100 is the first CXCR4 antagonist that is used for hematopoietic stem cell mobilization during transplantation in patients with non‐Hodgkin's lymphoma or multiple myeloma.^337^ Furthermore, AMD3100 and its derivate, AMD3465, are also capable of mobilizing cancer cells in the bone marrow to ultimately enhance the efficacies of conventional therapies.^338^ AMD3100 treatment in leukemic mice induced a ninefold increase in circulating leukemic cells.^339^ The combined treatment of chemotherapy and AMD3100 led to a reduced tumor burden and improved OS compared with those in mice received chemotherapy alone. A similar phenomenon was also observed in a phase 1/2 study of refractory acute myeloid leukemia.^339^ In addition to its chemosensitizing effects, AMD3100 synergistically interacts with antibodies targeting PD‐L1 and CTLA‐4 results in enhanced T‐cell infiltration into tumor tissues, yielding a greater anticancer response.^340^ Another CXCR4 antagonist, LY2510924, is also able of suppressing cancer growth and metastasis in multiple preclinical models.^341^, ^342^ Notably, LY2510924 was demonstrated to be clinically safe and well‐tolerated in advanced solid cancers including colorectal, lung, breast, and prostate cancers.^343^ Meanwhile, several CXCR4 small‐molecule antagonists—such as BKT140, PRX177561, and POL5551—are also capable of inhibiting cancer growth and metastasis.^344^, ^345^, ^346^ Recent studies have also suggested that peptide inhibitors targeting the amino‐terminal region of CXCR4 are also effective in inhibiting cancer growth, such as T22, TN14003, and CTCE‐9908.^347^, ^348^, ^349^ In preclinical models, TN14003 significantly inhibited pulmonary metastasis of melanomas, as well as breast cancers.^350^, ^351^ Similarly, CTCE‐9908 showed considerable inhibitory effects on cancer growth and metastasis in malignancies including osteosarcomas and melanomas, as well as breast and prostate cancers.^347^, ^352^, ^353^ These results suggest that disrupting the CXCL12‐CXCR4 axis represents a viable strategy for further anticancer drug developments. However, most of the above‐mentioned studies were carried out in the early course of cancer progression, and preclinical models may not fully simulate the clinical pathological process. Therefore, further clinical trials investigating the efficacies and safeties of CXCR4 antagonists are urgently needed in the context of advanced cancers.

### 5.2 Agents targeting tyrosine kinases

Given the activated CSF‐1/CSF‐1R signaling in multiple malignancies and their significant implications in predicting poor prognoses, developing agents to block the CSF‐1/CSF‐1R axis has attracted increasing research attention. Currently, both CSF‐1/CSF‐1R antibodies and CSF‐1R kinase inhibitors have been successfully developed for therapeutic regulation of macrophages. The key advantage of antibodies over kinase inhibitors is their higher selectivity and safety. Currently, several CSF‐1/CSF‐1R targeted antibodies have been developed. H27K15 is a novel competitive monoclonal antibody for CSF‐1 blockage that has been generated for cancer immunotherapy. Enzyme‐linked immunosorbent assays (ELISAs) have shown that H27K15 produces no cross‐reaction with other tyrosine kinases and can inhibit CSF‐1R phosphorylation by blocking CSF‐1.^362^ In preclinical models, H27K15 can decrease the secretion of both MCP‐1 and IL‐6, which play important roles in recruiting M2‐like macrophages. H27K15 is also capable of preventing monocyte differentiation into CD163^+^CD64^+^ M2‐like macrophages and induces their differentiation into CD14^‐^CD1a^+^ dendritic cells.^363^ RG7155 is designed as a humanized monoclonal antibody against CSF‐1R, with an IC_50_ of 0.3 nM.^364^ RG7155 can suppress CSF‐1R dimerization and therefore interrupt the formation of the CSF1/CSF1‐R complex. Consequently, RG7155 results in a significant reduction of CD68/CD163 macrophages.^363^ It has been reported that objective responses were observed in 83% of patients with diffuse‐type tenosynovial giant‐cell tumors (dt‐GCTs) following RG7155 treatment, even during a short‐course treatment in patients who are unable to undergo surgery.^365^ Additionally, RG7155 is also effective in increasing the CD8/CD4 ratio in multiple solid malignancies.^363^, ^364^ FPA008 is also a monoclonal antibody that disrupts the binding of CSF‐1R to its ligands and thus suppressing downstream receptor activation. The modulatory effects of FPA008 on macrophages and other immune cells in a variety of cancers are currently under evaluation by phase‐Ia/Ib clinical trials.^363^ With regard to adverse effects, increases in short‐lived enzymes (e.g., AST, LDH) are detected, which might be due to spontaneous inhibitory effects of antibodies on CSF‐1R^+^ Kupffer cells.^366^ Furthermore, common adverse effects also include fatigue, asthenia, facial edema, rash, and pruritus, but most of the events are only of grade 1 or 2 severities.

In addition to antibody therapies, multiple CSF‐1/CSF‐1R inhibitors are under development at clinical stages (Table 3). JNJ‐40346527 is a selective inhibitor of CSF‐1R with an IC_50_ of 3.2 nM. In a phase‐I/II clinical study of relapsed or refractory Hodgkin lymphoma, one patient out of 21 showed complete remission after JNJ‐40346527 treatment (150 mg/day) for approximately 1 year. Furthermore, 11 patients remained with disease stabilization.^367^ Notably, *in vivo* pharmacological activity showed that CSF‐1R phosphorylation was inhibited by 95% following JNJ‐40346527 treatment (150 mg/day). Meanwhile, higher doses (150–600 mg/day) also demonstrated acceptable tolerability and safety in healthy volunteers.^363^ GW2580 is an orally bioavailable CSF‐1R inhibitor with an IC_50_ of 60 nM.^368^ However, GW2580 shows limited inhibitory effects on macrophage recruitment and tumor growth. Priceman *et al*.^369^ treated mice to test the effects of GW2580 in blocking TAMs infiltration into growing tumors. Despite the concentration reaching as high as 160 mg/kg, only a slight decrease in TAMs was observed, while no notable changes in monocytes infiltration levels were observed in peripheral tissues including bone marrow, blood, and the spleen. Nevertheless, the combination treatment of GW2580 and VEGFR2 antibody efficiently abrogated the recruitment of TAMs and monocytic MDSCs and synergistically enhanced the antiangiogenesis response.^369^ Meanwhile, GW2580 was also found to increase gemcitabine therapeutic efficacy in pancreatic cancer compared to that of GW2580 alone.^370^ GW2580 was found to inhibit gemcitabine‐induced CSF‐1 overproduction in cancer cells and thus inhibited the recruitment of immunosuppressive myeloid cells into tumors.^370^

**TABLE 3:** Tyrosine kinases targeting agents in clinical trials

Drug/description	Sponsors	Phase/status	Tumor type	Treatment	Clinical trials.govIdentifier	First received
CSF‐1R/CSF‐1 inhibitor						
Emactuzumab/RG7155/RO5509554 (anti‐ CSF1R antibody)	M.D. Anderson Cancer Center	2^b^	Ovarian, fallopian tube, or primary peritoneal cancer	With paclitaxel and bevacizumab	NCT02923739	2016‐10‐05
	Hoffmann‐La Roche	1^b^	Advanced or metastatic solid cancers	With atezolizumab	NCT02323191	2014‐12‐23
	Hoffmann‐La Roche	1^a^	Advanced solid tumors	With RO7009789	NCT02760797	2016‐05‐04
	Hoffmann‐La Roche	1^a^	Advanced solid tumors	Monotherapy	NCT01494688 ^378^	2011‐12‐19
FPA008/Cabiralizumab (anti‐CSF1R antibody)	Bristol‐Myers Squibb	2^b^	Advanced pancreatic cancer	With nivolumab	NCT03336216	2017‐12‐08
	Five Prime Therapeutics	1/2^b^	Tenosynovial giant cell tumor	Monotherapy	NCT02471716	2015‐06‐15
	Five Prime Therapeutics	1a/b^b^	Advanced lung cancer, head and neck cancer, pancreatic cancer, ovarian cancer, renal cell carcinoma, and malignant glioma	With nivolumab	NCT02526017	2015‐08‐18
	Bristol‐Myers Squibb	1^a^	Advanced malignancies	With nivolumab	NCT03158272	2017‐05‐18
LY3022855/IMC‐CS4 (anti‐CSF1R antibody)	Eli Lilly and Company	1a/b^a^	Solid tumor	With durvalumab or tremelimumab	NCT02718911	2016‐03‐24
	Dana‐Farber Cancer Institute	1/2^b^	Melanoma	With vemurafenib and cobimetinib	NCT03101254	2017‐04‐05
	Sidney Kimmel Comprehensive Cancer Center at Johns Hopkins	1^b^	Pancreatic cancer	With GVAX, cyclophosphamide, and pembrolizumab	NCT03153410	2017‐05‐15
	Eli Lilly and Company	1^a^	Advanced breast or prostate cancer	Monotherapy	NCT02265536	2014‐10‐16
	Eli Lilly and Company	1^a^	Advanced solid tumors	Monotherapy	NCT01346358	2011‐05‐03
AMG820 (anti‐CSF1R antibody)	Amgen	1b/2^a^	Pancreatic cancer, colorectal cancer and non‐small cell lung cancer	With pembrolizumab	NCT02713529	2016‐03‐18
	Amgen	1^a^	Advanced solid tumors	Monotherapy	NCT01444404	2011‐09‐30
MCS110 (anti‐ CSF1 antibody)	Yonsei University	2^b^	Squamous cell carcinoma	With PDR001	NCT03785496	2018‐12‐24
	Seoul National University Hospital	2^b^	Gastric cancer	With PDR001	NCT03694977	2018‐10‐03
	Novartis Pharmaceuticals	2^b^	Advanced triple‐negative breast cancer	With carboplatin and gemcitabine	NCT02435680	2015‐05‐06
	Dana‐Farber Cancer Institute	1/2^b^	Melanoma	With dabrafenib and trametinib	NCT03455764	2018‐03‐07
	Novartis Pharmaceuticals	1b/2^b^	Triple‐negative breast cancer, pancreatic carcinoma, melanoma, and endometrial carcinoma	With PDR001	NCT02807844	2016‐06‐21
JNJ‐40346527 (CSF‐1R kinase inhibitor)	OHSU Knight Cancer Institute	2^b^	Relapsed/Refractory acute myeloid leukemia	Monotherapy	NCT03557970	2018‐06‐15
	Janssen Research & Development	1/2^a^	Relapsed or refractory Hodgkin lymphoma	Monotherapy	NCT01572519	2012‐04‐06
	M.D. Anderson Cancer Center	1^b^	Prostate cancer	Monotherapy	NCT03177460	2017‐06‐06
Pexidartinib/PLX3397 (CSF‐1R kinase inhibitor)	Daiichi Sankyo	3^b^	Pigmented villonodular synovitis or giant cell tumor of the tendon sheath	Monotherapy	NCT02371369^379^	2015‐02‐25
	Daiichi Sankyo	2^a^	Hodgkin lymphoma	Monotherapy	NCT01217229	2010‐10‐08
	Daiichi Sankyo	1/2^b^	Melanoma	Monotherapy	NCT02975700	2016‐12‐29
	National Cancer Institute	1/2^b^	Plexiform neurofibroma, precursor cell lymphoblastic leukemia lymphoma and acute prolymphocytic leukemia	Monotherapy	NCT02390752	2015‐03‐18
	Hope Rugo, MD	1b/2^a^	Metastatic breast cancer	With Eribulin	NCT01596751	2012‐05‐11
	Daiichi Sankyo	1b/2^b^	Glioblastoma	With radiation therapy and temozolomide	NCT01790503	2013‐02‐13
	Daiichi Sankyo	1/2^a^	Acute myeloid leukemia	Monotherapy	NCT01349049^380^	2011‐05‐06
	Gulam Manji	1^b^	Sarcoma and malignant peripheral nerve sheath tumors	With sirolimus	NCT02584647	2015‐10‐22
	Plexxikon	1^a^	Advanced solid tumors	With Paclitaxel	NCT01525602	2012‐02‐03
	Daiichi Sankyo	1^b^	Solid tumor	Monotherapy	NCT01004861^373^	2009‐10‐30
	Centre Leon Berard	1^a^	Advanced and metastatic colorectal cancer and pancreatic cancer	With durvalumab	NCT02777710	2016‐05‐19
ARRY‐382 (CSF‐1R kinase inhibitor)	Array Biopharma	1^a^	Metastatic cancer	Monotherapy	NCT01316822	2011‐03‐16
	Array BioPharma	1b/2^a^	Advanced solid tumors	With pembrolizumab	NCT02880371	2016‐08‐26
BLZ945 (CSF‐1R kinase inhibitor)	Novartis Pharmaceuticals	1/2^b^	Advanced solid tumors	With PDR001	NCT02829723	2016‐07‐12
DCC‐3014 (CSF‐1R kinase inhibitor)	Deciphera Pharmaceuticals	1/2^b^	Malignant solid tumors and tenosynovial giant cell tumor	Monotherapy	NCT03069469	2017‐03‐03
ENMD‐2076 (CSF‐1R kinase inhibitor)	University Health Network, Toronto	2#^a^	Ovarian clear cell carcinoma	Monotherapy	NCT01914510	2013‐08‐02
	University Health Network, Toronto	2^a^	Advanced/metastatic soft tissue sarcoma	Monotherapy	NCT01719744	2012‐11‐01
	CASI Pharmaceuticals	2^a^	Advanced adult hepatocellular carcinoma and advanced fibrolamellar carcinoma	Monotherapy	NCT02234986	2014‐09‐09
	CASI Pharmaceuticals	2^a^	Triple‐negative breast cancer	Monotherapy	NCT01639248^381^	2012‐07‐12
RON inhibitor						
Narnatumab/ IMC‐RON8 (anti‐RON antibody)	Eli Lilly and Company	1^a^	Solid tumors	Monotherapy	NCT01119456	2010‐05‐07
ASLAN002/BMS 777607 (RON kinase inhibitor)	Bristol‐Myers Squibb	1/2^a^	Advanced or metastatic solid tumor	Monotherapy	NCT00605618	2008‐01‐31
	Aslan Pharmaceuticals	1^a^	Advanced or metastatic solid tumor	Monotherapy	NCT01721148^382^	2012‐12‐04
Crizotinib/ PF‐02341066 (RON kinase inhibitor)	UNICANCER	2^b^	Hematologic cancers and solid tumors and metastatic cancer	Monotherapy	NCT02034981^383^	2014‐01‐14
	Dana‐Farber Cancer Institute	1^b^	Castration‐resistant prostate cancer	With enzalutamide	NCT02207504	2014‐08‐04
Foretinib/GSK1363089 (RON kinase inhibitor)	NCIC Clinical Trials Group	2^a^	Recurrent breast cancer	Monotherapy	NCT01147484^384^	2010‐06‐22
	GlaxoSmithKline	2^a^	Head and neck cancer	Monotherapy	NCT00725764	2008‐07‐30
	GlaxoSmithKline	2^a^	Papillary Renal‐Cell Carcinoma	Monotherapy	NCT00726323^385^	2008‐07‐31
	GlaxoSmithKline	2^a^	Metastatic gastric carcinoma	Monotherapy	NCT00725712^386^	2008‐07‐30
	GlaxoSmithKline	1^a^	Solid Tumors	Monotherapy	NCT00742131	2008‐08‐27
	GlaxoSmithKline	1^a^	Hepatocellular carcinoma	Monotherapy	NCT00920192^387^	2009‐06‐15

*Source*: clinicaltrials.gov.

^a^ Completed stage.

^b^ Ongoing stage, including both recruiting and nonrecruiting stages.

Apart from the above highly selective CSF‐1R inhibitors, several CSF‐1R inhibitors with lower specificity have been identified, which also exhibit broader inhibitory activity on other kinases (Table 3). PLX3397 represents one such inhibitor and has an IC_50_ of 13 nM on CSF‐1R. Additionally, PLX3397 also exhibits activities against KIT, FLT3, and PDGFRβ.^364^, ^371^, ^372^ PLX3397 displays anticancer effects in multiple malignancies. In patients with tenosynovial giant‐cell tumors, 52% of cases significantly responded to PLX3397 treatment with the disease control period lasting more than 8 months.^373^ In glioblastoma, PLX3397 not only prolonged the survival time but also slowed the disease progression rate.^374^ PLX3397 can easily cross the blood‐brain barrier and reach glioblastoma tissue, resulting in a significant reduction of macrophage numbers and invasiveness of cancer cells. In a phase‐II study of recurrent glioblastoma, PLX3397 treatment at a daily dose of 1000 mg showed acceptable tolerability and led to both CSF‐1R activity inhibition and TAMs infiltration reduction in tumor tissues.^372^ At present, multiple clinical trials are being designed and conducted to test the clinical efficacies of CSF‐1R inhibitors on cancer inhibition. However, CSF‐1R blockade alone usually achieves marginal therapeutic benefit, leading to the delay of tumor growth at most. Therefore, more clinical trials are of an urgent need to evaluate the combined effects of CSF‐1/CSF‐1R blockage and other treatment approaches including immunotherapies and standard treatment modalities. Particularly, results for combinations of CSF‐1/CSF‐1R blockage with immune‐checkpoint inhibitors or other immunotherapeutic strategies are eagerly expected.

Several investigators have focused on the development of inhibitors targeting RON receptors (Table 3). The small‐molecule ASLAN002 is a selective inhibitor of RON receptor with an IC_50_ < 500 nM.^375^ ASLAN002 can block MSP‐induced phosphorylation of RON. In a PyMT‐MSP breast cancer mouse model, ASLAN002 administration (50 mg/kg) remarkably inhibited lung metastasis by two‐ to threefold.^376^ Even when ASLAN002 was administrated after the formation of metastatic colonization, a four‐fold inhibitory response was observed, indicating that RON inhibition might be a novel therapeutic option to inhibit metastatic growth as an adjuvant treatment. A mechanistic study found that ASLAN002 resulted in a proinflammatory milieu in the TME, particularly in terms of an increase in TNF‐α‐positive macrophages.^376^ Additionally, CD8^+^ T cells act as critical factors influencing the efficacy of ASLAN002. The anti‐tumor colonization ability of ASLAN002 was greatly suppressed in the absence of cytotoxic CD8^+^ T cells, indicating the immune‐regulating cells may be primarily responsible for the anti‐cancer effects of ASLAN002. However, future research is necessary to explore the synergistic effects between ASLAN002 and immune‐checkpoint inhibitors. Another study revealed that in breast tumors harboring PIK3CA mutations, the anticancer effects of ASLAN002 were greatly limited, which were overcome by treatment with the PI3K inhibitor, NVP‐BKM120.^377^ Meanwhile, concurrent inhibition of RON and PI3K provided a more durable response in PIK3CA‐wild‐type breast cancer. These results suggest that further investigation focusing on the combined applications of small‐molecule inhibitors is worthwhile for improving clinical prognoses of cancer patients.

### 5.3 Bisphosphonates

Bisphosphonates are a family of inhibitors of osteoclast‐mediated bone resorption and are widely applied in the treatment of bone diseases including postmenopausal osteoporosis, Paget disease, and bone metastasis. The first generation of nonamino bisphosphonates, such as etidronate and clodronate, are metabolized intracellularly into cytotoxic adenosine‐triphosphate analogs that induce osteoclast cell death.^388^ Conversely, the second and third generations of bisphosphonates—such as ibandronate, risedronate, pamidronate, and zoledronic acid—are much more potent than nonamino compounds by inhibiting farnesyl‐pyrophosphate synthases, which also results in osteoclast apoptosis.^389^ Moreover, preclinical studies demonstrate that bisphosphonates may also exhibit extraskeletal therapeutic effects in mouse breast cancer xenografts.^390^ For example, zoledronic acid was found to be phagocyted by TAMs and thus induced TAMs apoptosis and repolarization toward M1‐like phenotype.^9^ In breast cancer patients, radiolabeled bisphosphonate was also found in the resected tumor infiltrated with TAMs and microcalcifications. Several studies have demonstrated the proinflammatory activity of amino‐bisphosphonates *in vitro*. Ibandronate was found to enhance LPS‐stimulated IL‐6 and IL‐1β secretion from mouse Raw264.7 macrophages.^391^ A remarkable elevation in TNFα expression was also observed following pamidronate and zoledronic‐acid administrations.^392^ In mouse xenografts, ibandronate treatment led to increased infiltration of extravasated leukocytes and rolling leukocytes, accompanied by the stimulated production of TNFα and IFN‐γ in peripheral blood, indicating its proinflammatory effects *in vivo*.^393^ In cancer patients treated with zoledronic acid, it was also found that the circulating concentrations of IFN‐γ, TNFα, and IL‐6 were all significantly elevated following drug administration, further confirming that amino‐bisphosphonates might promote M2‐like to M1‐like repolarization to trigger inflammatory responses.^394^ By contrast, nonamino bisphosphonates have been demonstrated to inhibit cancer progression via suppressing the growth of macrophages. Clodronate inhibits iNOS expression in Raw264.7 macrophages, accompanied by a significant reduction of IL‐6, IL‐1β, TNFα, and NO secretions at noncytotoxic concentrations.^395^ Therefore, nonamino bisphosphonates might suppress macrophages in a cytostatic but not cytotoxic manner. Based on the above findings, bisphosphonates have been commonly applied to deplete TAMs in preclinical models. Although bisphosphonates have yielded improved survival in various kinds of preclinical animal models, bisphosphonates treatment alone is not able to induce complete regression of tumors. In fact, the application of bisphosphonates may induce side effects on residential macrophages in normal tissues, thereby influencing antitumor innate immunity. Considering that the complete depletion of macrophages in tumors could lead to worse therapeutic outcomes in some cases, TAMs‐targeted reprogramming strategies that spare the potentially beneficial macrophages may be more preferable in the future.

### 5.4 Nanoparticle or exosomal‐targeted delivery

Although pharmaceutical inhibitors and monoclonal antibodies have been developed for TAMs‐targeting treatments, their therapeutic efficacies have been greatly limited by difficulties in penetrating biological barriers. Progress in nanotechnology has provided a valuable opportunity for improving drug‐loading/drug‐releasing parameters, biocompatibilities, and drug‐circulation time. The engineering of new nanoparticles with the ability to target and kill or reeducate TAMs has stood up as a promising strategy for cancer treatment in recent years. As an important member of the mononuclear phagocytic system, macrophages play a major role in nanomaterials recognition, uptake, processing, and clearance.^396^ Therefore, the loading of macrophages with nanomedicines may be used to profit from the high infiltration and the innate tumor homing abilities of macrophages, allowing the drug release in the bulk of the tumor. For example, Rao *et al*. reported that the magnetic nanoparticles can be used to promote the repolarization of the M2‐like TAMs as well as the systemic circulation and tumor accumulation of the loaded drugs.^397^ Notably, there are still some challenges for the effective targeting of TAMs with nanoparticles and their application in the clinic. The major one can be attributed to the undesirable clearance of nanoparticles by the mononuclear phagocyte system (macrophages) in clearance organs (liver, lung, or spleen) upon their intravenous injection. *In vivo* studies have demonstrated high nanomaterials sequestration by macrophages in clearance organs such as liver, spleen, and kidney.^396^ This is because that the immune system may recognize the components of nanoparticles (e.g., shell, core, surface‐decorating moieties, and cargoes) as foreign, and initiates an immune response through a complex process.^398^ Therefore, because of the synthetic origin, nanoparticles are usually recognized by the host immune system and are preferentially sequestered by macrophages in filtering organs.

Recently, exosomes have emerged as natural nano‐sized vesicles for drug delivery. Given their nanoscale size, excellent biocompatibility, potential capacity to express targeting ligands, and natural capacity to carry macromolecules, the use of exosomes for cancer treatment has raised considerable interest.^399^, ^400^ Due to the lipid bilayered membrane, exosomes exhibit good permeability and can cross most biological membranes, including the blood‐brain barrier.^401^ After reaching recipient cells, exosomes can activate certain signaling pathways by fusion to the plasma membrane and subsequent secretion of the contents into the cytoplasm of recipient cells. Alternatively, exosomes can enter cells through endocytic processes including receptor‐mediated endocytosis, micropinocytosis, and phagocytosis. Moreover, exosomes have significant advantages in drug delivery areas without inducing proinflammatory responses or adverse immune reactions. More importantly, the composition of exosomal surfaces permits modifications for enhancing interactions between recipient cells and exosomes. Surface modification of naturally purified exosomes with GE11, an epidermal growth factor, was demonstrated to increase the targeting potential of exosomes to EGFR^+^ breast cancer cells both *in vitro* and *in vivo*.^402^ Given these priorities, exosomes represent a promising candidate tool for the delivery of chemotherapeutics, as well as nucleic acids and phytochemicals. For example, doxorubicin treatment through the exosome delivery system induced a 10‐fold increase in cancer cell death when compared with free doxorubicin.^403^ Similarly, exosome‐loaded anti‐miR‐214 led to a remarkable inhibition of gastric tumor growth.^404^ Interestingly, curcumin has also been shown to be encapsulated into exosomes and to interfere with colon carcinogenesis (NCT01294072).

Given that exosomes can be preferentially sequestered by macrophages,^405^, ^406^ it is becoming an attractive vehicle for delivering genetic drugs or cytotoxic agents to inhibit TAMs. Exosomes containing miRNA‐155 and 125b2 derived from pancreatic cancer cells induced macrophage differentiation to the M1‐like phenotype.^407^ Similarly, exosomes transfected with wt‐p53 and miRNA‐125b secreted from colon cancer cells also mediated macrophage repolarization toward the M1‐like phenotype.^408^ Interestingly, epigallocatechin‐gallate‐treated breast cancer cells‐derived exosomes were also found to impair tumor growth by suppressing TAMs infiltration and M2‐like phenotype polarization.^137^ Recently, a commercialized engineered exosome carrying antisense oligonucleotide (exoASO) was developed by Codiak Bioscience. It was designed to deliver ASO to TAMs and to specifically inhibit the expression of immune‐suppressive transcription factors, including STAT6 and C/EBPT.^137^ Preclinical studies demonstrate that exoASO effectively reprograms M2‐like macrophages to a pro‐inflammatory M1‐like phenotype, promoting targeted anti‐tumor activity *in vivo*. *In vitro*, exoASO is preferentially taken up by M2‐like macrophages to a significantly greater extent than that of free ASO, resulting in a greater decrease of STAT6 and C/EBPβ mRNA levels. Subsequent gene expression analysis and cytokine assays showed a 40‐fold increase in TNFα and a 29‐fold decrease in IL‐10 associated with exoASO treatment, consistent with repolarization from immunosuppressive M2‐like macrophages to immune‐stimulatory M1‐like macrophages. All of these findings suggest that exosomes may represent promising drug‐delivery systems for modulating the biological functions of TAMs. Notably, there are still many difficulties to overcome before the successful application of the exosomal delivery system for TAMs modulation in the future. The most prominent one may be that different exosome isolation techniques have reported poor yield and loss of functional properties.^409^ To solve this limitation, reengineering TAMs‐targeted exosomes with the synthetic liposomes as a refined biomimetic nanostructure is suggested.^409^

### 5.5 Other therapeutic strategies

In recent years, multiple novel TAMs reprogramming strategies have been reported with promising future. For example, Christopher B *et al*. reported that R848, an agonist of toll‐like receptor 7 and 8 (TRL7/8), could significantly induce the M1‐like phenotype polarization of macrophages *in vitro*, while the R848‐loaded β‐cyclodextrin nanoparticles could promote the repolarization of TAMs toward an M1‐like phenotype in multiple tumor models *in vivo*.^410^ Feng *et al*. synthesized the novel TLR agonist of acGM using glucomannan polysaccharide with acetyl modification, which could specifically activate the TLR2 signaling and thus induce TAMs repolarization into an antitumor phenotype.^411^ Macrophages can also be targeted *via* CD40 agonists to boost the antitumor response. Gregory *et al*. reported that the CD40 agonist FGK45 could reactivate TAMs, which became tumoricidal and thus rapidly facilitated the depletion of tumor stroma and the tumor regression of pancreatic ductal adenocarcinoma.^412^ Blocking checkpoints on T cells have recently shown unprecedented, durable cures in the clinic. A similar strategy targeting macrophages is also developing by blocking CD47 from binding its receptor signal regulatory protein‐α (SIRPα) on macrophage surface.^413^ It has been reported that interfering of CD47‐SIRPα interaction by blocking antibodies could promote the phagocytosis of TAMs and consequently suppresses cholangiocarcinoma growth and metastasis.^414^ Nowadays, a number of CD47‐SIRPα axis blockers, such as Hu5F9‐G4, CC‐90002, SRF231, ALX148, and IBI188, are under clinical trials.^413^

## 6 CONCLUSIONS, CHALLENGES, AND PERSPECTIVES

With a deeper understanding of cancer immunology, targeting TAMs provides a novel approach to improve anticancer therapies. However, since there exists complex intercellular crosstalk involving TAMs in the TME, TAMs elimination may elicit multifaceted stromal reactions in the host that are unable to predict and may vary among patients. Additionally, some patients may be refractory to TAMs‐targeting therapies due to gene mutations. For example, it has been reported that certain single‐nucleotide polymorphisms in CSF‐1R can decrease the therapeutic efficacy of emactuzumab.^137^ Therefore, it is important and significant to explore the synergistic effects of TAMs‐targeting approaches with other immunomodulatory strategies, especially for checkpoint‐blockade–based therapies. Checkpoint inhibition unleashes the brakes, such as CTLA4 and PD‐1, to enhance the effective recognition and killing of tumor cells by immune cells. However, only approximately 20% of cancer patients respond to checkpoint‐inhibition therapies, where mixed responses can limit treatment effectiveness and cause drug resistance and/or distant metastasis. TAMs may act as important cellular components influencing the responsiveness of checkpoint‐blockade treatments. On one hand, TAMs express PD‐L1 and PD‐L2, which could aggravate the immunosuppressive TME and impair cytotoxic T lymphocytes against cancer cells.^14^, ^15^, ^415^ On the other hand, TAMs also expressed TREM2 (triggering receptor expressed on myeloid cells 2) that remodel the tumor myeloid landscape and weaken the responsiveness of anti‐PD‐1 immunotherapy.^416^ Currently, a variety of clinical trials are ongoing to examine the synergistic effects of TAMs‐targeting approaches with PD‐L1 or CTLA‐4 blockades. Preclinical studies have also demonstrated that CSF1R antagonists can boost the efficacy of checkpoint inhibition in mouse models of pancreatic, breast, cervical, and ovarian cancers. Moreover, in immunotherapy‐resistant pancreatic cancer patients, CSF‐1R antagonists combined with PD‐1‐targeting approaches result in partial responses in some patients. All of these findings indicate the promising future and significance of TAMs‐targeting strategies in cancer immunotherapies.

With an increasing number of available drugs for modulating TAMs to induce anti‐tumor responses by targeting different molecular targets, the next challenge will be how to deliver these immunoregulatory drugs into TAMs effectively and selectively while minimizing the harmful off‐target side effects. Although nanovesicles, such as exosomes, provide an ideal platform for drug loading and delivery and can be primarily internalized by macrophages, their biodistributions *in vivo* are largely affected by residential macrophages outside of tumors, such as Kupffer cells in the liver. To improve TAMs selectivity over residential macrophages, several studies have utilized the unique physical and chemical properties of the TME to optimize their targeting systems. For example, the acid‐sensitive PEG 2000 was applied to modify nanoparticles to selectively eliminate TAMs as PEG 2000 can be cleaved in the acidic TME to release cytotoxic drugs.^417^ This therapeutic strategy can minimize the off‐target effects of PEG 2000‐modified nanoparticles on resident macrophages. Additionally, targeting ligands have also been applied to improve the preferential delivery to TAMs *via* utilizing the specific ligand–receptor interactions. CD206 represents one of the most commonly targeted receptors for macrophage delivery because of its TAMs‐specific overexpression characteristic. As the native ligand of CD206, mannose can be conjugated to nanovesicles easily and result in an enhanced uptake by TAMs.^417^ TAMs also overexpress folate receptor β, the ligand of which is folic acid. Therefore, folic‐acid‐modified nanovesicles have been developed as an important cancer‐targeting strategy.^418^ Another factor influencing the anti‐tumor efficacy of nanovesicles is the ability of drug‐loaded macrophages to infiltrate into tumors. A recent study suggested that the size of nanovesicles can significantly alter the motilities of macrophages.^419^ Although smaller nanovesicles (30–50 nm) usually have higher uptake into macrophages compared to that of larger nanocarriers (100–500 nm), smaller nanovesicles were found to significantly retard the motilities of macrophages. This study suggested that 100‐nm nanovesicles provide a good balance for effective drug loading and macrophage migration. Hence, exosomes are an ideal and promising type of nanovesicle for developing TAMs‐targeting strategies.

Taken together, the pharmaceutical targets of TAMs and their corresponding drugs are rapidly being explored and developed. With the development of single‐cell sequencing, various “omic” analyses, and nanotechnology, our understanding of the heterogeneity of TAMs in the tumor milieu will be considerably enhanced. It is expected that future studies will further elucidate the clinical benefits resulting from combined therapies of TAMs‐targeting strategies with traditional treatments or immunotherapies. Meanwhile, a more advanced drug delivery system via exosome‐like nanovesicles is anticipated to help reduce the off‐target/side effects of TAMs‐targeting approaches.

## AUTHOR CONTRIBUTIONS

NW and ZYW conceived, wrote, and revised the article. SQW drew the figures and revised the article. XW generated the tables and added references. YFZ, BWY, JPZ, BP, and JLG participated in the work of reference selection and article check.

## COMPETING INTERESTS

The authors declare that they have no competing interests.

## AVAILABILITY OF DATA AND MATERIALS

Not applicable.

## References

[1] Ahmed F , Steele JC , Herbert JMJ , Steven NM , R B . Tumor stroma as a target in cancer. Curr Cancer Drug Targets. 2008;8(6):447‐453. 18781891 10.2174/156800908785699360

[2] Wagner J , Rapsomaniki MA , Chevrier S , et al. A single‐cell atlas of the tumor and immune ecosystem of human breast cancer. Cell. 2019;177(5):1330‐1345 e1318. 30982598 10.1016/j.cell.2019.03.005 PMC6526772

[3] Komohara Y , Fujiwara Y , Ohnishi K , Takeya M. Tumor‐associated macrophages: potential therapeutic targets for anti‐cancer therapy. Adv Drug Deliv Rev. 2016;99(Pt B):180‐185. 26621196 10.1016/j.addr.2015.11.009

[4] Kolios G , Valatas V , Kouroumalis E. Role of Kupffer cells in the pathogenesis of liver disease. World J Gastroenterol. 2006;12(46):7413‐7420. 17167827 10.3748/wjg.v12.i46.7413 PMC4087584

[5] Wang X , Zhao L , Zhang J , et al. Requirement for microglia for the maintenance of synaptic function and integrity in the mature retina. J Neurosci. 2016;36(9):2827‐2842. 26937019 10.1523/JNEUROSCI.3575-15.2016 PMC4879218

[6] Kimber I , Cumberbatch M , Dearman RJ. Langerhans cells and skin irritation In: Ai‐LeanChew HM, ed. Irritant Dermatitis. Berlin, New York, Heidelberg: Springer‐Verlag; 2006:383–391.

[7] Murray PJ , Allen JE , Biswas SK , et al. Macrophage activation and polarization: nomenclature and experimental guidelines. Immunity. 2014;41(1):14‐20. 25035950 10.1016/j.immuni.2014.06.008 PMC4123412

[8] Mills CD . M1 and M2 macrophages: oracles of health and disease. Crit Rev Immunol. 2012;32(6):463‐488. 23428224 10.1615/critrevimmunol.v32.i6.10

[9] Coscia M , Quaglino E , Iezzi M , et al. Zoledronic acid repolarizes tumour‐associated macrophages and inhibits mammary carcinogenesis by targeting the mevalonate pathway. J Cell Mol Med. 2010;14(12):2803‐2815. 19818098 10.1111/j.1582-4934.2009.00926.x PMC3822730

[10] De Palma M , Lewis CE. Macrophage regulation of tumor responses to anticancer therapies. Cancer Cell. 2013;23(3):277‐286. 23518347 10.1016/j.ccr.2013.02.013

[11] Sica A , Allavena P , Mantovani A. Cancer related inflammation: the macrophage connection. Cancer Lett. 2008;267(2):204‐215. 18448242 10.1016/j.canlet.2008.03.028

[12] Trombetta AC , Soldano S , Contini P , et al. A circulating cell population showing both M1 and M2 monocyte/macrophage surface markers characterizes systemic sclerosis patients with lung involvement. Respir Res. 2018;19(1):186. 30249259 10.1186/s12931-018-0891-z PMC6154930

[13] Van Gorp H , Delputte PL , Nauwynck HJ. Scavenger receptor CD163, a Jack‐of‐all‐trades and potential target for cell‐directed therapy. Mol Immunol. 2010;47(7‐8):1650‐1660. 20299103 10.1016/j.molimm.2010.02.008

[14] Gordon SR , Maute RL , Dulken BW , et al. PD‐1 expression by tumour‐associated macrophages inhibits phagocytosis and tumour immunity. Nature. 2017;545(7655):495‐499. 28514441 10.1038/nature22396 PMC5931375

[15] Cai J , Qi Q , Qian X , et al. The role of PD‐1/PD‐L1 axis and macrophage in the progression and treatment of cancer. J Cancer Res Clin Oncol. 2019;145(6):1377‐1385. 30963235 10.1007/s00432-019-02879-2 PMC11810350

[16] Fujimura T , Sato Y , Tanita K , et al. Serum levels of soluble CD163 and CXCL5 may be predictive markers for immune‐related adverse events in patients with advanced melanoma treated with nivolumab: a pilot study. Oncotarget. 2018;9(21):15542‐15551. 29643991 10.18632/oncotarget.24509 PMC5884646

[17] Fujimura T , Sato Y , Tanita K , et al. Serum level of soluble CD163 may be a predictive marker of the effectiveness of nivolumab in patients with advanced cutaneous melanoma. Front Oncol. 2018;8:530. 30510916 10.3389/fonc.2018.00530 PMC6252386

[18] Fransisca L , Curtis LT , James WM , et al. Macrophage polarization contributes to the anti‐tumoral efficacy of mesoporous nanovectors loaded with albumin‐bound paclitaxel. J Frontiers in Immunology. 2017;8:683. 10.3389/fimmu.2017.00693 PMC5472662 28670313

[19] Rakaee M , Busund LR , Jamaly S , et al. Prognostic value of macrophage phenotypes in resectable non‐small cell lung cancer assessed by multiplex immunohistochemistry. Neoplasia. 2019;21(3):282‐293. 30743162 10.1016/j.neo.2019.01.005 PMC6369140

[20] Yagi T , Baba Y , Okadome K , et al. Tumour‐associated macrophages are associated with poor prognosis and programmed death ligand 1 expression in oesophageal cancer. Eur J Cancer. 2019;111:38‐49. 30822683 10.1016/j.ejca.2019.01.018

[21] Zhou J , Wang XH , Zhao YX , et al. Cancer‐associated fibroblasts correlate with tumor‐associated macrophages infiltration and lymphatic metastasis in triple negative breast cancer patients. J Cancer. 2018;9(24):4635‐4641. 30588247 10.7150/jca.28583 PMC6299377

[22] Thacker JD , Dedhar S , Hogge DE. The effect of GM‐CSF and G‐CSF on the growth of human osteosarcoma cells in vitro and in vivo. Int J Cancer. 1994;56(2):236‐243. 7508889 10.1002/ijc.2910560216

[23] Wang Y , Han G , Wang K , et al. Tumor‐derived GM‐CSF promotes inflammatory colon carcinogenesis via stimulating epithelial release of VEGF. Cancer Res. 2014;74(3):716‐726. 24366884 10.1158/0008-5472.CAN-13-1459

[24] Garcia‐Mendoza MG , Inman DR , Ponik SM , et al. Neutrophils drive accelerated tumor progression in the collagen‐dense mammary tumor microenvironment. Breast Cancer Res. 2016;18(1):49. 27169366 10.1186/s13058-016-0703-7 PMC4864897

[25] Fritz JM , Tennis MA , Orlicky DJ , et al. Depletion of tumor‐associated macrophages slows the growth of chemically induced mouse lung adenocarcinomas. Front Immunol. 2014;5:587. 25505466 10.3389/fimmu.2014.00587 PMC4243558

[26] Cottone L , Valtorta S , Capobianco A , et al. Evaluation of the role of tumor‐associated macrophages in an experimental model of peritoneal carcinomatosis using(18)F‐FDG PET. J Nucl Med. 2011;52(11):1770‐1777. 22045707 10.2967/jnumed.111.089177

[27] Sullivan AR , Pixley FJ. CSF‐1R signaling in health and disease: a focus on the mammary gland. J Mammary Gland Biol Neoplasia. 2014;19(2):149‐159. 24912655 10.1007/s10911-014-9320-1

[28] Franklin RA , Liao W , Sarkar A , et al. The cellular and molecular origin of tumor‐associated macrophages. Science. 2014;344(6186):921‐925. 24812208 10.1126/science.1252510 PMC4204732

[29] Li X , Eckard J , Shah R , Malluck C , Frenkel K. Interleukin‐1α up‐regulation in vivo by a potent carcinogen 7,12‐dimethylbenz(a)anthracene(DMBA) and control of DMBA‐induced inflammatory responses. Cancer Res. 2002;62(2):417‐423. 11809690

[30] Weber C , Telerman SB , Reimer AS , et al. Macrophage infiltration and alternative activation during wound healing promote MEK1‐induced skin carcinogenesis. Cancer Res. 2016;76(4):805‐817. 26754935 10.1158/0008-5472.CAN-14-3676 PMC4757739

[31] Saxon JA , Sherrill TP , Polosukhin VV , Sai J , Blackwell TS. Epithelial NF‐κB signaling promotes EGFR‐driven lung carcinogenesis via macrophage recruitment. Oncoimmunology. 2016;5(6):e1168549. 27471643 10.1080/2162402X.2016.1168549 PMC4938365

[32] Leek RD , Lewis CE , Whitehouse R , Greenall M , Clarke J , Harris AL. Association of macrophage infiltration with angiogenesis and prognosis in invasive breast carcinoma. Cancer Res. 1996;56(20):4625‐4629. 8840975

[33] Negus RPM , Stamp GWH , Hadley J , Balkwill FR . Quantitative assessment of the leukocyte infiltrate in ovarian cancer and its relationship to the expression of C‐C chemokines. Am J Pathol. 1997;150(5):1723‐1734. 9137096 PMC1858213

[34] Leek RD , Harris AL. Tumor‐associated macrophages in breast cancer. J Mammary Gland Biol Neoplasia. 2002;7(2):177‐189. 12463738 10.1023/a:1020304003704

[35] Onita T , Ping GJ , Xuan JW , et al. Hypoxia‐induced, perinecrotic expression of endothelial Per‐ARNT‐Sim domain protein‐1/hypoxia‐inducible factor‐2α correlates with tumor progression, vascularization, and focal macrophage infiltration in bladder cancer. Clin Cancer Res. 2002;8(2):471‐480. 11839666

[36] Lin EY , Nguyen AV , Russell RG , Pollard JW. Colony‐stimulating factor 1 promotes progression of mammary tumors to malignancy. J Exp Med. 2001;193(6):727‐740. 11257139 10.1084/jem.193.6.727 PMC2193412

[37] Dirkx AE , Oude Egbrink MG , Wagstaff J , Griffioen AW . Monocyte/macrophage infiltration in tumors: modulators of angiogenesis. J Leukoc Biol. 2006;80(6):1183‐1196. 16997855 10.1189/jlb.0905495

[38] Sunderkötter C , Goebeler M , Schulze‐Osthoff K , Bhardwaj R , Sorg C. Macrophage‐derived angiogenesis factors. Pharmacol Ther. 1991;51(2):195‐216. 1784630 10.1016/0163-7258(91)90077-y

[39] Lin EY , Li JF , Bricard G , et al. Vascular endothelial growth factor restores delayed tumor progression in tumors depleted of macrophages. Mol Oncol. 2007;1(3):288‐302. 18509509 10.1016/j.molonc.2007.10.003 PMC2396497

[40] Pilar Cejudo‐Martín MM‐R , Ros J , Navasa M , et al. Hypoxia is an inducer of vasodilator agents in peritoneal macrophages of cirrhotic patients. Hepatology. 2002;36(5):1172‐1179. 12395327 10.1053/jhep.2002.36371

[41] Klimp AH , Hollema H , Kempinga C , van der Zee AG , de Vries EG , Daemen T. Expression of cyclooxygenase‐2 and inducible nitric oxide synthase in human ovarian tumors and tumor‐associated macrophages. Cancer Res. 2001;61(19):7305‐7309. 11585770

[42] Lewis CE , Pollard JW. Distinct role of macrophages in different tumor microenvironments. Cancer Res. 2006;66(2):605‐612. 16423985 10.1158/0008-5472.CAN-05-4005

[43] Nozawa H , Chiu C , Hanahan D. Infiltrating neutrophils mediate the initial angiogenic switch in a mouse model of multistage carcinogenesis. Proc Natl Acad Sci U S A. 2006;103(33):12493‐12498. 16891410 10.1073/pnas.0601807103 PMC1531646

[44] Houghton AM , Grisolano JL , Baumann ML , et al. Macrophage elastase(matrix metalloproteinase‐12) suppresses growth of lung metastases. Cancer Res. 2006;66(12):6149‐6155. 16778188 10.1158/0008-5472.CAN-04-0297

[45] Mantovani A , Marchesi F , Malesci A , Laghi L , Allavena P. Tumour‐associated macrophages as treatment targets in oncology. Nat Rev Clin Oncol. 2017;14(7):399‐416. 28117416 10.1038/nrclinonc.2016.217 PMC5480600

[46] Ruffell B , Chang‐Strachan D , Chan V , et al. Macrophage IL‐10 blocks CD8+ T cell‐dependent responses to chemotherapy by suppressing IL‐12 expression in intratumoral dendritic cells. Cancer Cell. 2014;26(5):623‐637. 25446896 10.1016/j.ccell.2014.09.006 PMC4254570

[47] Shalapour S , Karin M. Pas de Deux: control of anti‐tumor immunity by cancer‐associated inflammation. Immunity. 2019;51(1):15‐26. 31315033 10.1016/j.immuni.2019.06.021 PMC6640850

[48] Obermajer N , Muthuswamy R , Lesnock J , Edwards RP , Kalinski P. Positive feedback between PGE2 and COX2 redirects the differentiation of human dendritic cells toward stable myeloid‐derived suppressor cells. Blood. 2011;118(20):5498‐5505. 21972293 10.1182/blood-2011-07-365825 PMC3217352

[49] Kawano Y , Roccaro AM , Ghobrial IM , Azzi J. Multiple myeloma and the immune microenvironment. Curr Cancer Drug Targets. 2017;17(9):806‐818. 28201978 10.2174/1568009617666170214102301

[50] El‐Khoueiry AB , Sangro B , Yau T , et al. Nivolumab in patients with advanced hepatocellular carcinoma(CheckMate 040): an open‐label, non‐comparative, phase 1/2 dose escalation and expansion trial. Lancet. 2017;389(10088):2492‐2502. 28434648 10.1016/S0140-6736(17)31046-2 PMC7539326

[51] Zhu Y , Knolhoff BL , Meyer MA , et al. CSF1/CSF1R blockade reprograms tumor‐infiltrating macrophages and improves response to T‐cell checkpoint immunotherapy in pancreatic cancer models. Cancer Res. 2014;74(18):5057‐5069. 25082815 10.1158/0008-5472.CAN-13-3723 PMC4182950

[52] HS S , H B , C B , R V , et al. FcγR interaction is not required for effective anti‐PD‐L1 immunotherapy but can add additional benefit depending on the tumor model. Int J Cancer. 2019;144(2):345. 30259976 10.1002/ijc.31899

[53] Liu H , Wang J , Zhang M , et al. Jagged1 promotes aromatase inhibitor resistance by modulating tumor‐associated macrophage differentiation in breast cancer patients. Breast Cancer Res Treat. 2017;166(1):95‐107. 28730338 10.1007/s10549-017-4394-2

[54] D'Errico G , Alonso‐Nocelo M , Vallespinos M , et al. Tumor‐associated macrophage‐secreted 14‐3‐3ζ signals via AXL to promote pancreatic cancer chemoresistance. Oncogene. 2019;38(27):5469‐5485. 30936462 10.1038/s41388-019-0803-9

[55] Forssell J , Oberg A , Henriksson ML , Stenling R , Jung A , Palmqvist R. High macrophage infiltration along the tumor front correlates with improved survival in colon cancer. Clin Cancer Res. 2007;13(5):1472‐1479. 17332291 10.1158/1078-0432.CCR-06-2073

[56] Zheng Y , Yang J , Qian J , et al. PSGL‐1/selectin and ICAM‐1/CD18 interactions are involved in macrophage‐induced drug resistance in myeloma. Leukemia. 2013;27(3):702‐710. 22996336 10.1038/leu.2012.272 PMC3652581

[57] Zheng P , Chen L , Yuan X , et al. Exosomal transfer of tumor‐associated macrophage‐derived miR‐21 confers cisplatin resistance in gastric cancer cells. J Exp Clin Cancer Res. 2017;36(1):53. 28407783 10.1186/s13046-017-0528-y PMC5390430

[58] Giurisato E , Lonardi S , Telfer B , et al. Extracellular‐regulated protein kinase 5‐mediated control of p21 expression promotes macrophage proliferation associated with tumor growth and metastasis. Cancer Res. 2020;80(16):3319‐3330. 32561530 10.1158/0008-5472.CAN-19-2416 PMC7611207

[59] Su S , Liu Q , Chen J , et al. A positive feedback loop between mesenchymal‐like cancer cells and macrophages is essential to breast cancer metastasis. Cancer Cell. 2014;25(5):605‐620. 24823638 10.1016/j.ccr.2014.03.021

[60] Fu XT , Dai Z , Song K , et al. Macrophage‐secreted IL‐8 induces epithelial‐mesenchymal transition in hepatocellular carcinoma cells by activating the JAK2/STAT3/Snail pathway. Int J Oncol. 2015;46(2):587‐596. 25405790 10.3892/ijo.2014.2761

[61] Ravi J , Elbaz M , Wani NA , Nasser MW , Ganju RK. Cannabinoid receptor‐2 agonist inhibits macrophage induced EMT in non‐small cell lung cancer by downregulation of EGFR pathway. Mol Carcinog. 2016;55(12):2063‐2076. 26741322 10.1002/mc.22451 PMC7063844

[62] Helm O , Held‐Feindt J , Grage‐Griebenow E , et al. Tumor‐associated macrophages exhibit pro‐ and anti‐inflammatory properties by which they impact on pancreatic tumorigenesis. Int J Cancer. 2014;135(4):843‐861. 24458546 10.1002/ijc.28736

[63] Wu Y , Deng J , Rychahou PG , Qiu S , Evers BM , Zhou BP. Stabilization of snail by NF‐kappaB is required for inflammation‐induced cell migration and invasion. Cancer Cell. 2009;15(5):416‐428. 19411070 10.1016/j.ccr.2009.03.016 PMC2881229

[64] Kawata M , Koinuma D , Ogami T , et al. TGF‐beta‐induced epithelial‐mesenchymal transition of A549 lung adenocarcinoma cells is enhanced by pro‐inflammatory cytokines derived from RAW 264.7 macrophage cells. J Biochem. 2012;151(2):205‐216. 22161143 10.1093/jb/mvr136

[65] Vasiljeva O , Papazoglou A , Kruger A , et al. Tumor cell‐derived and macrophage‐derived cathepsin B promotes progression and lung metastasis of mammary cancer. Cancer Res. 2006;66(10):5242‐5250. 16707449 10.1158/0008-5472.CAN-05-4463

[66] Kessenbrock K , Plaks V , Werb Z. Matrix metalloproteinases: regulators of the tumor microenvironment. Cell. 2010;141(1):52‐67. 20371345 10.1016/j.cell.2010.03.015 PMC2862057

[67] Gocheva V , Wang HW , Gadea BB , et al. IL‐4 induces cathepsin protease activity in tumor‐associated macrophages to promote cancer growth and invasion. Genes Dev. 2010;24(3):241‐255. 20080943 10.1101/gad.1874010 PMC2811826

[68] Sangaletti S , Di Carlo E , Gariboldi S , et al. Macrophage‐derived SPARC bridges tumor cell‐extracellular matrix interactions toward metastasis. Cancer Res. 2008;68(21):9050‐9059. 18974151 10.1158/0008-5472.CAN-08-1327

[69] Sidani M , Wyckoff J , Xue C , Segall JE , Condeelis J. Probing the microenvironment of mammary tumors using multiphoton microscopy. J Mammary Gland Biol Neoplasia. 2006;11(2):151‐163. 17106644 10.1007/s10911-006-9021-5

[70] Qian B , Deng Y , Im JH , et al. A distinct macrophage population mediates metastatic breast cancer cell extravasation, establishment and growth. PLoS One. 2009;4(8):e6562. 19668347 10.1371/journal.pone.0006562 PMC2721818

[71] Nierodzik ML , Karpatkin S. Thrombin induces tumor growth, metastasis, and angiogenesis: evidence for a thrombin‐regulated dormant tumor phenotype. Cancer Cell. 2006;10(5):355‐362. 17097558 10.1016/j.ccr.2006.10.002

[72] Gil‐Bernabe AM , Ferjancic S , Tlalka M , et al. Recruitment of monocytes/macrophages by tissue factor‐mediated coagulation is essential for metastatic cell survival and premetastatic niche establishment in mice. Blood. 2012;119(13):3164‐3175. 22327225 10.1182/blood-2011-08-376426

[73] Chen Q , Zhang XH , Massague J. Macrophage binding to receptor VCAM‐1 transmits survival signals in breast cancer cells that invade the lungs. Cancer Cell. 2011;20(4):538‐549. 22014578 10.1016/j.ccr.2011.08.025 PMC3293160

[74] Kaplan RN , Riba RD , Zacharoulis S , et al. VEGFR1‐positive haematopoietic bone marrow progenitors initiate the pre‐metastatic niche. Nature. 2005;438(7069):820‐827. 16341007 10.1038/nature04186 PMC2945882

[75] Sceneay J , Smyth MJ , Moller A. The pre‐metastatic niche: finding common ground. Cancer Metastasis Rev. 2013;32(3‐4):449‐464. 23636348 10.1007/s10555-013-9420-1

[76] Kaplan RN , Psaila B , Lyden D. Bone marrow cells in the “pre‐metastatic niche”: within bone and beyond. Cancer Metastasis Rev. 2006;25(4):521‐529. 17186383 10.1007/s10555-006-9036-9

[77] Eisenblaetter M , Flores‐Borja F , Lee JJ , et al. Visualization of tumor‐immune interaction—target‐specific imaging of S100A8/A9 reveals pre‐metastatic niche establishment. Theranostics. 2017;7(9):2392‐2401. 28744322 10.7150/thno.17138 PMC5525744

[78] Zheng Y , Wang N , Wang S , et al. XIAOPI formula inhibits the pre‐metastatic niche formation in breast cancer via suppressing TAMs/CXCL1 signaling. Cell Commun Signal. 2020;18(1):48. 32213179 10.1186/s12964-020-0520-6 PMC7098160

[79] Balkwill FR. The chemokine system and cancer. J Pathol. 2012;226(2):148‐157. 21989643 10.1002/path.3029

[80] Zlotnik A , Yoshie O . Chemokines: a new classification system and their role in immunity. Immunity. 2000;12(2):121‐127. 10714678 10.1016/s1074-7613(00)80165-x

[81] Mollaoglu G , Jones A , Wait SJ , et al. The lineage‐defining transcription factors SOX2 and NKX2‐1 determine lung cancer cell fate and shape the tumor immune microenvironment. Immunity. 2018;49(4):764‐779 e769. 30332632 10.1016/j.immuni.2018.09.020 PMC6197489

[82] Conti I , Rollins BJ. CCL2(monocyte chemoattractant protein‐1) and cancer. Semin Cancer Biol. 2004;14(3):149‐154. 15246049 10.1016/j.semcancer.2003.10.009

[83] Sawanobori Y , Ueha S , Kurachi M , et al. Chemokine‐mediated rapid turnover of myeloid‐derived suppressor cells in tumor‐bearing mice. Blood. 2008;111(12):5457‐5466. 18375791 10.1182/blood-2008-01-136895

[84] Fujita M , Kohanash G , Fellows W , et al. COX‐2 blockade suppresses gliomagenesis by inhibiting CCL2‐mediated accumulation of myeloid‐derived suppressor cells. Cancer Res. 2011;71:1788‐1788. 10.1158/0008-5472.CAN-10-3055 PMC3075086 21324923

[85] Ma Y , Mattarollo SR , Adjemian S , et al. CCL2/CCR2‐dependent recruitment of functional antigen‐presenting cells into tumors upon chemotherapy. Cancer Res. 2014;74(2):436‐445. 24302580 10.1158/0008-5472.CAN-13-1265

[86] Luboshits G , Shina S , Kaplan O , et al. Elevated expression of the CC chemokine regulated on activation, normal T cell expressed and secreted(RANTES) in advanced breast carcinoma. Cancer Res. 1999;59(18):4681‐4687. 10493525

[87] Duell EJ , Casella DP , Burk RD , Kelsey KT , Holly EA. Inflammation, genetic polymorphisms in proinflammatory genes TNF‐A, RANTES, and CCR5, and risk of pancreatic adenocarcinoma. Cancer Epidemiol Biomarkers Prev. 2006;15(4):726‐731. 16614115 10.1158/1055-9965.EPI-05-0797

[88] Tsukishiro S , Suzumori N , Nishikawa H , Arakawa A , Suzumori K. Elevated serum RANTES levels in patients with ovarian cancer correlate with the extent of the disorder. Gynecol Oncol. 2006;102(3):542‐545. 16510173 10.1016/j.ygyno.2006.01.029

[89] Vaday GG , Peehl DM , Kadam PA , Lawrence DM. Expression of CCL5(RANTES) and CCR5 in prostate cancer. Prostate. 2006;66(2):124‐134. 16161154 10.1002/pros.20306

[90] Zhang Q , Qin J , Zhong L , et al. CCL5‐Mediated Th2 immune polarization promotes metastasis in luminal breast cancer. Cancer Res. 2015;75(20):4312‐4321. 26249173 10.1158/0008-5472.CAN-14-3590

[91] Chang LY , Lin YC , Mahalingam J , et al. Tumor‐derived chemokine CCL5 enhances TGF‐beta‐mediated killing of CD8(+) T cells in colon cancer by T‐regulatory cells. Cancer Res. 2012;72(5):1092‐1102. 22282655 10.1158/0008-5472.CAN-11-2493

[92] Lv M , Xu Y , Tang R , et al. miR141‐CXCL1‐CXCR2 signaling‐induced Treg recruitment regulates metastases and survival of non‐small cell lung cancer. Mol Cancer Ther. 2014;13(12):3152‐3162. 25349304 10.1158/1535-7163.MCT-14-0448

[93] Wang D , Sun H , Wei J , Cen B , DuBois RN. CXCL1 is critical for premetastatic niche formation and metastasis in colorectal cancer. Cancer Res. 2017;77(13):3655‐3665. 28455419 10.1158/0008-5472.CAN-16-3199 PMC5877403

[94] Obermajer N , Muthuswamy R , Odunsi K , Edwards RP , Kalinski P. PGE(2)‐induced CXCL12 production and CXCR4 expression controls the accumulation of human MDSCs in ovarian cancer environment. Cancer Res. 2011;71(24):7463‐7470. 22025564 10.1158/0008-5472.CAN-11-2449 PMC4993027

[95] Martins‐Green M , Feugate JE . The 9E3/CEF4 gene product is a chemotactic and angiogenic factor that can initiate the wound‐healing cascade in vivo. Cytokine. 1998;10(7):522‐535. 9702416 10.1006/cyto.1997.0311

[96] Teicher BA , Fricker SP. CXCL12(SDF‐1)/CXCR4 pathway in cancer. Clin Cancer Res. 2010;16(11):2927‐2931. 20484021 10.1158/1078-0432.CCR-09-2329

[97] Wang Y , Liu J , Jiang Q , et al. Human adipose‐derived mesenchymal stem cell‐secreted CXCL1 and CXCL8 facilitate breast tumor growth by promoting angiogenesis. Stem Cells. 2017;35(9):2060‐2070. 28514506 10.1002/stem.2643

[98] Tang KH , Ma S , Lee TK , et al. CD133(+) liver tumor‐initiating cells promote tumor angiogenesis, growth, and self‐renewal through neurotensin/interleukin‐8/CXCL1 signaling. Hepatology. 2012;55(3):807‐820. 21994122 10.1002/hep.24739

[99] Wang D , Wang H , Brown J , et al. CXCL1 induced by prostaglandin E2 promotes angiogenesis in colorectal cancer. J Exp Med. 2006;203(4):941‐951. 16567391 10.1084/jem.20052124 PMC2118273

[100] Azenshtein E , Luboshits G , Shina S , et al. The CC chemokine RANTES in breast carcinoma progression: regulation of expression and potential mechanisms of promalignant activity. Cancer Res. 2002;62(4):1093‐1102. 11861388

[101] Youngs SJ , Ali SA , Taub DD , Rees RC. Chemokines induce migrational responses in human breast carcinoma cell lines. Int J Cancer. 1997;71(2):257‐266. 9139852 10.1002/(sici)1097-0215(19970410)71:2<257::aid-ijc22>3.0.co;2-d

[102] Prest SJ , Rees RC , Murdoch C , et al. Chemokines induce the cellular migration of MCF‐7 human breast carcinoma cells: subpopulations of tumour cells display positive and negative chemotaxis and differential in vivo growth potentials. Clin Exp Metastasis. 1999;17(5):389‐396. 10651305 10.1023/a:1006657109866

[103] Miller LJ , Kurtzman SH , Wang Y , Anderson KH , Lindquist RR , Kreutzer DL. Expression of interleukin‐8 receptors on tumor cells and vascular endothelial cells in human breast cancer tissue. Anticancer Res. 1998;18(1A):77‐81. 9568059

[104] Kato M , Kitayama J , Kazama S , Nagawa H. Expression pattern of CXC chemokine receptor‐4 is correlated with lymph node metastasis in human invasive ductal carcinoma. Breast Cancer Res. 2003;5(5):R144‐150. 12927045 10.1186/bcr627 PMC314431

[105] Acharyya S , Oskarsson T , Vanharanta S , et al. A CXCL1 paracrine network links cancer chemoresistance and metastasis. Cell. 2012;150(1):165‐178. 22770218 10.1016/j.cell.2012.04.042 PMC3528019

[106] Zhuang H , Cao G , Kou C , Liu T. CCL2/CCR2 axis induces hepatocellular carcinoma invasion and epithelial‐mesenchymal transition in vitro through activation of the Hedgehog pathway. Oncol Rep. 2018;39(1):21‐30. 29115520 10.3892/or.2017.6069 PMC5783597

[107] Lee CC , Lai JH , Hueng DY , et al. Disrupting the CXCL12/CXCR4 axis disturbs the characteristics of glioblastoma stem‐like cells of rat RG2 glioblastoma. Cancer Cell Int. 2013;13(1):85. 23961808 10.1186/1475-2867-13-85 PMC3765790

[108] Ben‐Baruch A. The multifaceted roles of chemokines in malignancy. Cancer Metastasis Rev. 2006;25(3):357‐371. 17016763 10.1007/s10555-006-9003-5

[109] Tokunaga R , Zhang W , Naseem M , et al. CXCL9, CXCL10, CXCL11/CXCR3 axis for immune activation—a target for novel cancer therapy. Cancer Treat Rev. 2018;63:40‐47. 29207310 10.1016/j.ctrv.2017.11.007 PMC5801162

[110] Aldinucci D , Borghese C , Casagrande N. The CCL5/CCR5 axis in cancer progression. Cancers(Basel). 2020;12(7):1765. 10.3390/cancers12071765 PMC7407580 32630699

[111] Huang R , Wang S , Wang N , et al. CCL5 derived from tumor‐associated macrophages promotes prostate cancer stem cells and metastasis via activating beta‐catenin/STAT3 signaling. Cell Death Dis. 2020;11(4):234. 32300100 10.1038/s41419-020-2435-y PMC7162982

[112] Neo SY , Lundqvist A. The multifaceted roles of CXCL9 within the tumor microenvironment. Adv Exp Med Biol. 2020;1231:45‐51. 32060845 10.1007/978-3-030-36667-4_5

[113] Wu X , Schulte BC , Zhou Y , et al. Depletion of M2‐like tumor‐associated macrophages delays cutaneous T‐cell lymphoma development in vivo. J Invest Dermatol. 2014;134(11):2814‐2822. 24780929 10.1038/jid.2014.206

[114] Furudate S , Fujimura T , Kakizaki A , Hidaka T , Asano M , Aiba S. Tumor‐associated M2 macrophages in mycosis fungoides acquire immunomodulatory function by interferon alpha and interferon gamma. J Dermatol Sci. 2016;83(3):182‐189. 27342040 10.1016/j.jdermsci.2016.05.004

[115] Kakizaki A , Fujimura T , Furudate S , et al. Immunomodulatory effect of peritumorally administered interferon‐beta on melanoma through tumor‐associated macrophages. Oncoimmunology. 2015;4(11):e1047584. 26451326 10.1080/2162402X.2015.1047584 PMC4589056

[116] Miller CH , Maher SG , Young HA. Clinical use of interferon‐gamma. Ann N Y Acad Sci. 2009;1182:69‐79. 20074276 10.1111/j.1749-6632.2009.05069.x PMC6574079

[117] Schmidt T , Ben‐Batalla I , Schultze A , Loges S. Macrophage‐tumor crosstalk: role of TAMR tyrosine kinase receptors and of their ligands. Cell Mol Life Sci. 2012;69(9):1391‐1414. 22076650 10.1007/s00018-011-0863-7 PMC11115155

[118] Lemieux SM , Hadden MK. Targeting the fibroblast growth factor receptors for the treatment of cancer. Anticancer Agents Med Chem. 2013;13(5):748‐761. 23272905 10.2174/18715206113139990080

[119] Im D , Jung K , Yang S , Aman W , Hah JM. Discovery of 4‐arylamido 3‐methyl isoxazole derivatives as novel FMS kinase inhibitors. Eur J Med Chem. 2015;102:600‐610. 26318067 10.1016/j.ejmech.2015.08.031

[120] Okugawa Y , Toiyama Y , Ichikawa T , et al. Colony‐stimulating factor‐1 and colony‐stimulating factor‐1 receptor co‐expression is associated with disease progression in gastric cancer. Int J Oncol. 2018;53(2):737‐749. 29767252 10.3892/ijo.2018.4406

[121] Steins A , van Mackelenbergh MG , van der Zalm AP , et al. High‐grade mesenchymal pancreatic ductal adenocarcinoma drives stromal deactivation through CSF‐1. Embo Rep. 2020;21(5):e48780. 32173982 10.15252/embr.201948780 PMC7202203

[122] Jang JY , Lee JK , Jeon YK , Kim CW. Exosome derived from epigallocatechin gallate treated breast cancer cells suppresses tumor growth by inhibiting tumor‐associated macrophage infiltration and M2 polarization. BMC Cancer. 2013;13:421. 24044575 10.1186/1471-2407-13-421 PMC3848851

[123] Achkova D , Maher J. Role of the colony‐stimulating factor(CSF)/CSF‐1 receptor axis in cancer. Biochem Soc Trans. 2016;44(2):333‐341. 27068937 10.1042/BST20150245

[124] Scholl SM , Pallud C , Beuvon F , et al. Anti‐colony‐stimulating factor‐1 antibody staining in primary breast adenocarcinomas correlates with marked inflammatory cell infiltrates and prognosis. J Natl Cancer Inst. 1994;86(2):120‐126. 8271294 10.1093/jnci/86.2.120

[125] Wyckoff JB , Wang Y , Lin EY , et al. Direct visualization of macrophage‐assisted tumor cell intravasation in mammary tumors. Cancer Res. 2007;67(6):2649‐2656. 17363585 10.1158/0008-5472.CAN-06-1823

[126] Wiehagen KR , Girgis NM , Yamada DH , et al. Combination of CD40 agonism and CSF‐1R blockade reconditions tumor‐associated macrophages and drives potent antitumor immunity. Cancer Immunol Res. 2017;5(12):1109‐1121. 29097420 10.1158/2326-6066.CIR-17-0258

[127] Zhong WQ , Chen G , Zhang W , et al. M2‐polarized macrophages in keratocystic odontogenic tumor: relation to tumor angiogenesis. Sci Rep. 2015;5:15586. 26508096 10.1038/srep15586 PMC4623606

[128] Welm AL , Sneddon JB , Taylor C , et al. The macrophage‐stimulating protein pathway promotes metastasis in a mouse model for breast cancer and predicts poor prognosis in humans. Proc Natl Acad Sci U S A. 2007;104(18):7570‐7575. 17456594 10.1073/pnas.0702095104 PMC1855278

[129] Yuan ZL , Guan YJ , Wang L , Wei W , Kane AB , Chin YE. Central role of the threonine residue within the p+1 loop of receptor tyrosine kinase in STAT3 constitutive phosphorylation in metastatic cancer cells. Mol Cell Biol. 2004;24(21):9390‐9400. 15485908 10.1128/MCB.24.21.9390-9400.2004 PMC522220

[130] Liu QP , Fruit K , Ward J , Correll PH. Negative regulation of macrophage activation in response to IFN‐gamma and lipopolysaccharide by the STK/RON receptor tyrosine kinase. J Immunol. 1999;163(12):6606‐6613. 10586055

[131] Eyob H , Ekiz HA , Welm AL. RON promotes the metastatic spread of breast carcinomas by subverting antitumor immune responses. Oncoimmunology. 2013;2(9):e25670. 24327933 10.4161/onci.25670 PMC3850023

[132] Davra V , Kumar S , Geng K , et al. Axl and Mertk receptors cooperate to promote breast cancer progression by combined oncogenic signaling and evasion of host anti‐tumor immunity. Cancer Res. 2020 10.1158/0008-5472.can-20-2066 PMC9999365 33239426

[133] Zizzo G , Hilliard BA , Monestier M , Cohen PL. Efficient clearance of early apoptotic cells by human macrophages requires M2c polarization and MerTK induction. J Immunol. 2012;189(7):3508‐3520. 22942426 10.4049/jimmunol.1200662 PMC3465703

[134] Caetano MS , Younes AI , Barsoumian HB , et al. Triple therapy with MerTK and pd1 inhibition plus radiotherapy promotes abscopal antitumor immune responses. Clin Cancer Res. 2019;25(24):7576‐7584. 31540976 10.1158/1078-0432.CCR-19-0795 PMC6911635

[135] Zhou Y , Fei M , Zhang G , et al. Blockade of the phagocytic receptor MerTK on tumor‐associated macrophages enhances P2×7R‐dependent STING activation by tumor‐derived cGAMP. Immunity. 2020;52(2):357‐373 e359. 32049051 10.1016/j.immuni.2020.01.014

[136] Dehne N , Mora J , Namgaladze D , Weigert A , Brune B. Cancer cell and macrophage cross‐talk in the tumor microenvironment. Curr Opin Pharmacol. 2017;35:12‐19. 28538141 10.1016/j.coph.2017.04.007

[137] Jang JY , Lee JK , Jeon YK , Kim CW. Exosome derived from epigallocatechin gallate treated breast cancer cells suppresses tumor growth by inhibiting tumor‐associated macrophage infiltration and M2 polarization. BMC Cancer. 2013;13(1):421. 24044575 10.1186/1471-2407-13-421 PMC3848851

[138] Wang T , Liu H , Lian G , Zhang SY , Wang X , Jiang C. HIF1alpha‐induced glycolysis metabolism is essential to the activation of inflammatory macrophages. Mediators Inflamm. 2017;2017:9029327. 29386753 10.1155/2017/9029327 PMC5745720

[139] Liu D , Chang C , Lu N , et al. Comprehensive proteomics analysis reveals metabolic reprogramming of tumor‐associated macrophages stimulated by the tumor microenvironment. J Proteome Res. 2017;16(1):288‐297. 27809537 10.1021/acs.jproteome.6b00604

[140] Arts RJ , Plantinga TS , Tuit S , et al. Transcriptional and metabolic reprogramming induce an inflammatory phenotype in non‐medullary thyroid carcinoma‐induced macrophages. Oncoimmunology. 2016;5(12):e1229725. 28123869 10.1080/2162402X.2016.1229725 PMC5213309

[141] Huber R , Meier B , Otsuka A , et al. Tumour hypoxia promotes melanoma growth and metastasis via high mobility group Box‐1 and M2‐like macrophages. Sci Rep. 2016;6:29914. 27426915 10.1038/srep29914 PMC4947927

[142] Brand A , Singer K , Koehl GE , et al. LDHA‐associated lactic acid production blunts tumor immunosurveillance by T and NK cells. Cell Metab. 2016;24(5):657‐671. 27641098 10.1016/j.cmet.2016.08.011

[143] Lim SO , Li CW , Xia W , et al. EGFR Signaling enhances aerobic glycolysis in triple‐negative breast cancer cells to promote tumor growth and immune escape. Cancer Res. 2016;76(5):1284‐1296. 26759242 10.1158/0008-5472.CAN-15-2478 PMC4775355

[144] Kellum JA , Song M , Li J. Lactic and hydrochloric acids induce different patterns of inflammatory response in LPS‐stimulated RAW 264.7 cells. Am J Physiol Regul Integr Comp Physiol. 2004;286(4):R686‐692. 14695114 10.1152/ajpregu.00564.2003

[145] Artyomov MN , Sergushichev A , Schilling JD. Integrating immunometabolism and macrophage diversity. Semin Immunol. 2016;28(5):417‐424. 27771140 10.1016/j.smim.2016.10.004 PMC5333784

[146] Currie E , Schulze A , Zechner R , Walther TC , Farese RV, Jr . Cellular fatty acid metabolism and cancer. Cell Metab. 2013;18(2):153‐161. 23791484 10.1016/j.cmet.2013.05.017 PMC3742569

[147] Huang SC , Everts B , Ivanova Y , et al. Cell‐intrinsic lysosomal lipolysis is essential for alternative activation of macrophages. Nat Immunol. 2014;15(9):846‐855. 25086775 10.1038/ni.2956 PMC4139419

[148] Castegna A , Gissi R , Menga A , et al. Pharmacological targets of metabolism in disease: opportunities from macrophages. Pharmacol Ther. 2020;210:107521. 32151665 10.1016/j.pharmthera.2020.107521

[149] Li AC , Binder CJ , Gutierrez A , et al. Differential inhibition of macrophage foam‐cell formation and atherosclerosis in mice by PPARalpha, beta/delta, and gamma. J Clin Invest. 2004;114(11):1564‐1576. 15578089 10.1172/JCI18730 PMC529277

[150] Kang K , Reilly SM , Karabacak V , et al. Adipocyte‐derived Th2 cytokines and myeloid PPARdelta regulate macrophage polarization and insulin sensitivity. Cell Metab. 2008;7(6):485‐495. 18522830 10.1016/j.cmet.2008.04.002 PMC2586840

[151] Schumann T , Adhikary T , Wortmann A , et al. Deregulation of PPARbeta/delta target genes in tumor‐associated macrophages by fatty acid ligands in the ovarian cancer microenvironment. Oncotarget. 2015;6(15):13416‐13433. 25968567 10.18632/oncotarget.3826 PMC4537024

[152] Yakeu G , Butcher L , Isa S , et al. Low‐intensity exercise enhances expression of markers of alternative activation in circulating leukocytes: roles of PPARgamma and Th2 cytokines. Atherosclerosis. 2010;212(2):668‐673. 20723894 10.1016/j.atherosclerosis.2010.07.002

[153] Palmieri EM , Menga A , Martin‐Perez R , et al. Pharmacologic or genetic targeting of glutamine synthetase skews macrophages toward an M1‐like phenotype and inhibits tumor metastasis. Cell Rep. 2017;20(7):1654‐1666. 28813676 10.1016/j.celrep.2017.07.054 PMC5575233

[154] Kornyeyev DY. Inhibition of glutamine synthetase activity by phosphinothricin results in disappearance of the peak M2 of the chlorophyll fluorescence induction curve. Photosynthetica. 2000;36(4):601‐604.

[155] Jha AK , Huang SC , Sergushichev A , et al. Network integration of parallel metabolic and transcriptional data reveals metabolic modules that regulate macrophage polarization. Immunity. 2015;42(3):419‐430. 25786174 10.1016/j.immuni.2015.02.005

[156] Rath M , Muller I , Kropf P , Closs EI , Munder M. Metabolism via arginase or nitric oxide synthase: two competing arginine pathways in macrophages. Front Immunol. 2014;5:532. 25386178 10.3389/fimmu.2014.00532 PMC4209874

[157] Curran JN , Winter DC , Bouchier‐Hayes D. Biological fate and clinical implications of arginine metabolism in tissue healing. Wound Repair Regen. 2006;14(4):376‐386. 16939563 10.1111/j.1743-6109.2006.00151.x

[158] Zhao Q , Kuang DM , Wu Y , et al. Activated CD69+ T cells foster immune privilege by regulating IDO expression in tumor‐associated macrophages. J Immunol. 2012;188(3):1117‐1124. 22184722 10.4049/jimmunol.1100164

[159] Song F , Hurtado del Pozo C , Rosario R , et al. RAGE regulates the metabolic and inflammatory response to high‐fat feeding in mice. Diabetes. 2014;63(6):1948‐1965. 24520121 10.2337/db13-1636 PMC4030112

[160] Chen MC , Chen KC , Chang GC , et al. RAGE acts as an oncogenic role and promotes the metastasis of human lung cancer. Cell Death Dis. 2020;11(4):265. 32327633 10.1038/s41419-020-2432-1 PMC7181650

[161] Wang S , Wang N , Zheng Y , et al. Caveolin‐1 inhibits breast cancer stem cells via c‐Myc‐mediated metabolic reprogramming. Cell Death Dis. 2020;11(6):450. 32528105 10.1038/s41419-020-2667-x PMC7290025

[162] Wang Q , Zhu G , Cao X , Dong J , Song F , Niu Y. Blocking AGE‐RAGE signaling improved functional disorders of macrophages in diabetic wound. J Diabetes Res. 2017;2017:1428537. 29119117 10.1155/2017/1428537 PMC5651124

[163] Jin X , Yao T , Zhou Z , et al. Advanced glycation end products enhance macrophages polarization into M1 phenotype through activating RAGE/NF‐kappaB pathway. Biomed Res Int. 2015;2015:732450. 26114112 10.1155/2015/732450 PMC4465680

[164] Lan J , Sun L , Xu F , et al. M2 macrophage‐derived exosomes promote cell migration and invasion in colon cancer. Cancer Res. 2019;79(1):146‐158. 30401711 10.1158/0008-5472.CAN-18-0014

[165] Li X , Wang X. The emerging roles and therapeutic potential of exosomes in epithelial ovarian cancer. Mol Cancer. 2017;16(1):92. 28506269 10.1186/s12943-017-0659-y PMC5433006

[166] Melo SA , Luecke LB , Kahlert C , et al. Glypican‐1 identifies cancer exosomes and detects early pancreatic cancer. Nature. 2015;523(7559):177‐182. 26106858 10.1038/nature14581 PMC4825698

[167] Melo SA , Sugimoto H , O'Connell JT , et al. Cancer exosomes perform cell‐independent microRNA biogenesis and promote tumorigenesis. Cancer Cell. 2014;26(5):707‐721. 25446899 10.1016/j.ccell.2014.09.005 PMC4254633

[168] Caradec J , Kharmate G , Hosseini‐Beheshti E , Adomat H , Gleave M , Guns E. Reproducibility and efficiency of serum‐derived exosome extraction methods. Clin Biochem. 2014;47(13‐14):1286‐1292. 24956264 10.1016/j.clinbiochem.2014.06.011

[169] Aslan C , Maralbashi S , Salari F , et al. Tumor‐derived exosomes: Implication in angiogenesis and antiangiogenesis cancer therapy. J Cell Physiol. 2019;234(10):16885‐16903. 30793767 10.1002/jcp.28374

[170] Kosaka N , Iguchi H , Hagiwara K , Yoshioka Y , Takeshita F , Ochiya T. Neutral sphingomyelinase 2(nSMase2)‐dependent exosomal transfer of angiogenic microRNAs regulate cancer cell metastasis. J Biol Chem. 2013;288(15):10849‐10859. 23439645 10.1074/jbc.M112.446831 PMC3624465

[171] Challagundla KB , Wise PM , Paolo N , et al. Exosome‐mediated transfer of microRNAs within the tumor microenvironment and neuroblastoma resistance to chemotherapy. J Natl Cancer Inst. 2015;107(7):djv135. 25972604 10.1093/jnci/djv135 PMC4651042

[172] Fabbr M. MicroRNAs bind to toll‐like receptors to induce prometastatic inflammatory response. Proc Natl Acad Sci U S A. 2012;109(31):E2110‐E2116. 22753494 10.1073/pnas.1209414109 PMC3412003

[173] Saha S , Aranda E , Hayakawa Y , et al. Macrophage‐derived extracellular vesicle‐packaged WNTs rescue intestinal stem cells and enhance survival after radiation injury. Nat Commun. 2016;7:13096. 27734833 10.1038/ncomms13096 PMC5065628

[174] Monica F , Laura C , Gennaro C. The epithelial‐to‐mesenchymal transition in breast cancer: focus on basal‐like carcinomas. Cancers. 2017;9(10):134. 10.3390/cancers9100134 PMC5664073 28974015

[175] Jinjie W , Haiyan L , Hongyu X , Xianrui W , Ping L. The malignant role of exosomes in the communication among colorectal cancer cell, macrophage and microbiome. Carcinogenesis. 2019;40(5):601‐610. 30864655 10.1093/carcin/bgy138

[176] Jieru, Zhou , Xiaoduan, Li , Xiaoli, Wu , et al. Exosomes released from tumor‐associated macrophages transfer miRNAs that induce a Treg/Th17 cell imbalance in epithelial ovarian cancer. Cancer Immunol Res. 2018;6(12):1578‐1592. 30396909 10.1158/2326-6066.CIR-17-0479

[177] Han R , Gu S , Zhang Y , et al. Estrogen promotes progression of hormone‐dependent breast cancer through CCL2‐CCR2 axis by upregulation of Twist via PI3K/AKT/NF‐κB signaling. Sci Rep. 2018;8(1):9575. 29934505 10.1038/s41598-018-27810-6 PMC6015029

[178] Li D , Ji H , Niu X , et al. Tumor‐associated macrophages secrete CC‐chemokine ligand 2 and induce tamoxifen resistance by activating PI3K/Akt/mTOR in breast cancer. Cancer Sci. 2020;111(1):47‐58. 31710162 10.1111/cas.14230 PMC6942430

[179] Yao M , Fang W , Smart C , et al. CCR2 chemokine receptors enhance growth and cell‐cycle progression of breast cancer cells through SRC and PKC activation. Mol Cancer Res. 2019;17(2):604‐617. 30446625 10.1158/1541-7786.MCR-18-0750 PMC6359961

[180] Lee GT , Kwon SJ , Kim J , et al. WNT5A induces castration‐resistant prostate cancer via CCL2 and tumour‐infiltrating macrophages. Br J Cancer. 2018;118(5):670‐678. 29381686 10.1038/bjc.2017.451 PMC5846063

[181] Roca H , Varsos Z , Pienta KJ. CCL2 protects prostate cancer PC3 cells from autophagic death via phosphatidylinositol 3‐kinase/AKT‐dependent survivin up‐regulation. J Biol Chem. 2008;283(36):25057‐25073. 18611860 10.1074/jbc.M801073200 PMC2529129

[182] Ito Y , Ishiguro H , Kobayashi N , et al. Adipocyte‐derived monocyte chemotactic protein‐1(MCP‐1) promotes prostate cancer progression through the induction of MMP‐2 activity. Prostate. 2015;75(10):1009‐1019. 25917126 10.1002/pros.22972

[183] Zhuang H , Cao G , Kou C , Liu T. CCL2/CCR2 axis induces hepatocellular carcinoma invasion and epithelial‐mesenchymal transition in vitro through activation of the Hedgehog pathway. Oncol Rep. 2018;39(1):21‐30. 29115520 10.3892/or.2017.6069 PMC5783597

[184] Dagouassat M , Suffee N , Hlawaty H , et al. Monocyte chemoattractant protein‐1(MCP‐1)/CCL2 secreted by hepatic myofibroblasts promotes migration and invasion of human hepatoma cells. Int J Cancer. 2010;126(5):1095‐1108. 19642141 10.1002/ijc.24800

[185] He M , Yu W , Chang C , et al. Estrogen receptor α promotes lung cancer cell invasion via increase of and cross‐talk with infiltrated macrophages through the CCL2/CCR2/MMP9 and CXCL12/CXCR4 signaling pathways. Mol Oncol. 2020;14(8):1779‐1799. 32356397 10.1002/1878-0261.12701 PMC7400793

[186] Zhou L , Jiang Y , Liu X , et al. Promotion of tumor‐associated macrophages infiltration by elevated neddylation pathway via NF‐κB‐CCL2 signaling in lung cancer. Oncogene. 2019;38(29):5792‐5804. 31243299 10.1038/s41388-019-0840-4

[187] Ding M , He SJ , Yang J. MCP‐1/CCL2 mediated by autocrine loop of PDGF‐BB promotes invasion of lung cancer cell by recruitment of macrophages via CCL2‐CCR2 axis. J Interferon Cytokine Res. 2019;39(4):224‐232. 30730243 10.1089/jir.2018.0113

[188] Mizutani K , Sud S , McGregor NA , et al. The chemokine CCL2 increases prostate tumor growth and bone metastasis through macrophage and osteoclast recruitment. Neoplasia. 2009;11(11):1235‐1242. 19881959 10.1593/neo.09988 PMC2767225

[189] Xu W , Wei Q , Han M , et al. CCL2‐SQSTM1 positive feedback loop suppresses autophagy to promote chemoresistance in gastric cancer. Int J Biol Sci. 2018;14(9):1054‐1066. 29989092 10.7150/ijbs.25349 PMC6036739

[190] Chen K , Liu Q , Tsang LL , et al. Human MSCs promotes colorectal cancer epithelial‐mesenchymal transition and progression via CCL5/β‐catenin/Slug pathway. Cell Death Dis. 2017;8(5):e2819. 28542126 10.1038/cddis.2017.138 PMC5520690

[191] Kato T , Fujita Y , Nakane K , et al. CCR1/CCL5 interaction promotes invasion of taxane‐resistant PC3 prostate cancer cells by increasing secretion of MMPs 2/9 and by activating ERK and Rac signaling. Cytokine. 2013;64(1):251‐257. 23876400 10.1016/j.cyto.2013.06.313

[192] Huang CY , Fong YC , Lee CY , et al. CCL5 increases lung cancer migration via PI3K, Akt and NF‐kappaB pathways. Biochem Pharmacol. 2009;77(5):794‐803. 19073147 10.1016/j.bcp.2008.11.014

[193] Xia L , Zhu X , Zhang L , Xu Y , Chen G , Luo J . EZH2 enhances expression of CCL5 to promote recruitment of macrophages and invasion in lung cancer. Biotechnol Appl Biochem. 2020; 10.1002/bab.1875. PMC7818479 31855281

[194] Walens A , DiMarco AV , Lupo R , Kroger BR , Damrauer JS , Alvarez JV. CCL5 promotes breast cancer recurrence through macrophage recruitment in residual tumors. Elife. 2019;8:e43653 30990165 10.7554/eLife.43653 PMC6478432

[195] Zazo S , González‐Alonso P , Martín‐Aparicio E , et al. Autocrine CCL5 effect mediates trastuzumab resistance by ERK pathway activation in HER2‐positive breast cancer. Mol Cancer Ther. 2020;19(8):1696‐1707. 32404410 10.1158/1535-7163.MCT-19-1172

[196] Murooka TT , Rahbar R , Fish EN. CCL5 promotes proliferation of MCF‐7 cells through mTOR‐dependent mRNA translation. Biochem Biophys Res Commun. 2009;387(2):381‐386. 19607806 10.1016/j.bbrc.2009.07.035

[197] Long H , Xie R , Xiang T , et al. Autocrine CCL5 signaling promotes invasion and migration of CD133+ ovarian cancer stem‐like cells via NF‐κB‐mediated MMP‐9 upregulation. Stem Cells. 2012;30(10):2309‐2319. 22887854 10.1002/stem.1194

[198] Tang S , Xiang T , Huang S , et al. Ovarian cancer stem‐like cells differentiate into endothelial cells and participate in tumor angiogenesis through autocrine CCL5 signaling. Cancer Lett. 2016;376(1):137‐147. 27033454 10.1016/j.canlet.2016.03.034

[199] Singh SK , Mishra MK , Rivers BM , Gordetsky JB , Bae S , Singh R. Biological and clinical significance of the CCR5/CCL5 axis in hepatocellular carcinoma. Cancers(Basel). 2020;12(4):883. 10.3390/cancers12040883 PMC7226629 32260550

[200] Wang X , Lang M , Zhao T , et al. Cancer‐FOXP3 directly activated CCL5 to recruit FOXP3(+)Treg cells in pancreatic ductal adenocarcinoma. Oncogene. 2017;36(21):3048‐3058. 27991933 10.1038/onc.2016.458 PMC5454319

[201] Singh SK , Mishra MK , Eltoum IA , Bae S , Lillard JW, Jr. , Singh R . CCR5/CCL5 axis interaction promotes migratory and invasiveness of pancreatic cancer cells. Sci Rep. 2018;8(1):1323. 29358632 10.1038/s41598-018-19643-0 PMC5778036

[202] Liu C , Yao Z , Wang J , et al. Macrophage‐derived CCL5 facilitates immune escape of colorectal cancer cells via the p65/STAT3‐CSN5‐PD‐L1 pathway. Cell Death Differ. 2020;27(6):1765‐1781. 31802034 10.1038/s41418-019-0460-0 PMC7244707

[203] Wang HC , Chen CW , Yang CL , et al. Tumor‐associated macrophages promote epigenetic silencing of gelsolin through DNA methyltransferase 1 in gastric cancer cells. Cancer Immunol Res. 2017;5(10):885‐897. 28835422 10.1158/2326-6066.CIR-16-0295

[204] Yang T , Chen M , Yang X , et al. Down‐regulation of KLF5 in cancer‐associated fibroblasts inhibit gastric cancer cells progression by CCL5/CCR5 axis. Cancer Biol Ther. 2017;18(10):806‐815. 28934010 10.1080/15384047.2017.1373219 PMC5678703

[205] Mellado M , de Ana AM , Moreno MC , Martínez C , Rodríguez‐Frade JM. A potential immune escape mechanism by melanoma cells through the activation of chemokine‐induced T cell death. Curr Biol. 2001;11(9):691‐696. 11369232 10.1016/s0960-9822(01)00199-3

[206] Zhao C , Zheng S , Yan Z , Deng Z , Wang R , Zhang B. CCL18 promotes the invasion and metastasis of breast cancer through Annexin A2. Oncol Rep. 2020;43(2):571‐580. 31894281 10.3892/or.2019.7426

[207] Chen J , Yao Y , Gong C , et al. CCL18 from tumor‐associated macrophages promotes breast cancer metastasis via PITPNM3. Cancer Cell. 2011;19(4):541‐555. 21481794 10.1016/j.ccr.2011.02.006 PMC3107500

[208] Lin X , Chen L , Yao Y , et al. CCL18‐mediated down‐regulation of miR98 and miR27b promotes breast cancer metastasis. Oncotarget. 2015;6(24):20485‐20499. 26244871 10.18632/oncotarget.4107 PMC4653020

[209] Jiang X , Huang Z , Sun X , et al. CCL18‐NIR1 promotes oral cancer cell growth and metastasis by activating the JAK2/STAT3 signaling pathway. BMC Cancer. 2020;20(1):632. 32641093 10.1186/s12885-020-07073-z PMC7346480

[210] Wang H , Liang X , Li M , et al. Chemokine(CC motif) ligand 18 upregulates Slug expression to promote stem‐cell like features by activating the mammalian target of rapamycin pathway in oral squamous cell carcinoma. Cancer Sci. 2017;108(8):1584‐1593. 28574664 10.1111/cas.13289 PMC5543498

[211] Wang Q , Tang Y , Yu H , et al. CCL18 from tumor‐cells promotes epithelial ovarian cancer metastasis via mTOR signaling pathway. Mol Carcinog. 2016;55(11):1688‐1699. 26457987 10.1002/mc.22419 PMC5057350

[212] Lane D , Matte I , Laplante C , et al. CCL18 from ascites promotes ovarian cancer cell migration through proline‐rich tyrosine kinase 2 signaling. Mol Cancer. 2016;15(1):58. 27613122 10.1186/s12943-016-0542-2 PMC5017134

[213] Shi L , Zhang B , Sun X , et al. CC chemokine ligand 18(CCL18) promotes migration and invasion of lung cancer cells by binding to Nir1 through Nir1‐ELMO1/DOC180 signaling pathway. Mol Carcinog. 2016;55(12):2051‐2062. 26756176 10.1002/mc.22450

[214] Liu X , Xu X , Deng W , et al. CCL18 enhances migration, invasion and EMT by binding CCR8 in bladder cancer cells. Mol Med Rep. 2019;19(3):1678‐1686. 30592282 10.3892/mmr.2018.9791 PMC6390063

[215] Ye H , Zhou Q , Zheng S , et al. Tumor‐associated macrophages promote progression and the Warburg effect via CCL18/NF‐kB/VCAM‐1 pathway in pancreatic ductal adenocarcinoma. Cell Death Dis. 2018;9(5):453. 29670110 10.1038/s41419-018-0486-0 PMC5906621

[216] Hou X , Zhang Y , Qiao H. CCL18 promotes the invasion and migration of gastric cancer cells via ERK1/2/NF‐κB signaling pathway. Tumour Biol. 2016;37(1):641‐651. 26242263 10.1007/s13277-015-3825-0

[217] Wang B , Shi L , Sun X , Wang L , Wang X , Chen C. Production of CCL20 from lung cancer cells induces the cell migration and proliferation through PI3K pathway. J Cell Mol Med. 2016;20(5):920‐929. 26968871 10.1111/jcmm.12781 PMC4831357

[218] Wei W , Zhao X , Zhu J , et al. lncRNAu50535 promotes the progression of lung cancer by activating CCL20/ERK signaling. Oncol Rep. 2019;42(5):1946‐1956. 31545478 10.3892/or.2019.7302 PMC6775802

[219] Kadomoto S , Izumi K , Hiratsuka K , et al. Tumor‐associated macrophages induce migration of renal cell carcinoma cells via activation of the CCL20‐CCR6 axis. Cancers(Basel). 2019;12(1):89. 10.3390/cancers12010089 PMC7017081 31905918

[220] Yu X , Yuan Z , Yang Z , et al. The novel long noncoding RNA u50535 promotes colorectal cancer growth and metastasis by regulating CCL20. Cell Death Dis. 2018;9(7):751. 29970882 10.1038/s41419-018-0771-y PMC6030363

[221] Brand S , Olszak T , Beigel F , et al. Cell differentiation dependent expressed CCR6 mediates ERK‐1/2, SAPK/JNK, and Akt signaling resulting in proliferation and migration of colorectal cancer cells. J Cell Biochem. 2006;97(4):709‐723. 16215992 10.1002/jcb.20672

[222] Wang D , Yang L , Yu W , et al. Colorectal cancer cell‐derived CCL20 recruits regulatory T cells to promote chemoresistance via FOXO1/CEBPB/NF‐κB signaling. J Immunother Cancer. 2019;7(1):215. 31395078 10.1186/s40425-019-0701-2 PMC6688336

[223] Geismann C , Grohmann F , Dreher A , et al. Role of CCL20 mediated immune cell recruitment in NF‐κB mediated TRAIL resistance of pancreatic cancer. Biochim Biophys Acta Mol Cell Res. 2017;1864(5):782‐796. 28188806 10.1016/j.bbamcr.2017.02.005

[224] Liu B , Jia Y , Ma J , et al. Tumor‐associated macrophage‐derived CCL20 enhances the growth and metastasis of pancreatic cancer. Acta Biochim Biophys Sin(Shanghai). 2016;48(12):1067‐1074. 27797715 10.1093/abbs/gmw101

[225] Chen W , Qin Y , Wang D , et al. CCL20 triggered by chemotherapy hinders the therapeutic efficacy of breast cancer. PLoS Biol. 2018;16(7):e2005869. 30052635 10.1371/journal.pbio.2005869 PMC6082578

[226] Lee SK , Park KK , Kim HJ , et al. Human antigen R‐regulated CCL20 contributes to osteolytic breast cancer bone metastasis. Sci Rep. 2017;7(1):9610. 28851919 10.1038/s41598-017-09040-4 PMC5575024

[227] Marsigliante S , Vetrugno C , Muscella A. Paracrine CCL20 loop induces epithelial‐mesenchymal transition in breast epithelial cells. Mol Carcinog. 2016;55(7):1175‐1186. 26154142 10.1002/mc.22360

[228] Marsigliante S , Vetrugno C , Muscella A. CCL20 induces migration and proliferation on breast epithelial cells. J Cell Physiol. 2013;228(9):1873‐1883. 23460117 10.1002/jcp.24349

[229] Han G , Wu D , Yang Y , Li Z , Zhang J , Li C. CrkL meditates CCL20/CCR6‐induced EMT in gastric cancer. Cytokine. 2015;76(2):163‐169. 26044596 10.1016/j.cyto.2015.05.009

[230] Maolake A , Izumi K , Shigehara K , et al. Tumor‐associated macrophages promote prostate cancer migration through activation of the CCL22‐CCR4 axis. Oncotarget. 2017;8(6):9739‐9751. 28039457 10.18632/oncotarget.14185 PMC5354767

[231] Wei C , Yang C , Wang S , et al. M2 macrophages confer resistance to 5‐fluorouracil in colorectal cancer through the activation of CCL22/PI3K/AKT signaling. Onco Targets Ther. 2019;12:3051‐3063. 31114248 10.2147/OTT.S198126 PMC6489624

[232] Li ZQ , Wang HY , Zeng QL , et al. p65/miR‐23a/CCL22 axis regulated regulatory T cells recruitment in hepatitis B virus positive hepatocellular carcinoma. Cancer Med. 2020;9(2):711‐723. 31769216 10.1002/cam4.2611 PMC6970059

[233] Wang N , Liu W , Zheng Y , et al. CXCL1 derived from tumor‐associated macrophages promotes breast cancer metastasis via activating NF‐κB/SOX4 signaling. Cell Death Dis. 2018;9(9):880. 30158589 10.1038/s41419-018-0876-3 PMC6115425

[234] Cabrero‐de Las Heras S , Martínez‐Balibrea E. CXC family of chemokines as prognostic or predictive biomarkers and possible drug targets in colorectal cancer. World J Gastroenterol. 2018;24(42):4738‐4749. 30479461 10.3748/wjg.v24.i42.4738 PMC6235799

[235] Yang C , Yu H , Chen R , et al. CXCL1 stimulates migration and invasion in ERnegative breast cancer cells via activation of the ERK/MMP2/9 signaling axis. Int J Oncol. 2019;55(3):684‐696. 31322183 10.3892/ijo.2019.4840 PMC6685590

[236] Wen Z , Liu Q , Wu J , et al. Fibroblast activation protein α‐positive pancreatic stellate cells promote the migration and invasion of pancreatic cancer by CXCL1‐mediated Akt phosphorylation. Ann Transl Med. 2019;7(20):532. 31807514 10.21037/atm.2019.09.164 PMC6861799

[237] Taki M , Abiko K , Baba T , et al. Snail promotes ovarian cancer progression by recruiting myeloid‐derived suppressor cells via CXCR2 ligand upregulation. Nat Commun. 2018;9(1):1685. 29703902 10.1038/s41467-018-03966-7 PMC5923228

[238] Yung MM , Tang HW , Cai PC , et al. GRO‐α and IL‐8 enhance ovarian cancer metastatic potential via the CXCR2‐mediated TAK1/NFκB signaling cascade. Theranostics. 2018;8(5):1270‐1285. 29507619 10.7150/thno.22536 PMC5835935

[239] Dong YL , Kabir SM , Lee ES , Son DS. CXCR2‐driven ovarian cancer progression involves upregulation of proinflammatory chemokines by potentiating NF‐κB activation via EGFR‐transactivated Akt signaling. PLoS One. 2013;8(12):e83789. 24376747 10.1371/journal.pone.0083789 PMC3869803

[240] Lu Y , Dong B , Xu F , et al. CXCL1‐LCN2 paracrine axis promotes progression of prostate cancer via the Src activation and epithelial‐mesenchymal transition. Cell Commun Signal. 2019;17(1):118. 31500632 10.1186/s12964-019-0434-3 PMC6734451

[241] Kuo PL , Shen KH , Hung SH , Hsu YL. CXCL1/GROα increases cell migration and invasion of prostate cancer by decreasing fibulin‐1 expression through NF‐κB/HDAC1 epigenetic regulation. Carcinogenesis. 2012;33(12):2477‐2487. 23027620 10.1093/carcin/bgs299

[242] Zhou Z , Xia G , Xiang Z , et al. A C‐X‐C Chemokine receptor type 2‐dominated cross‐talk between tumor cells and macrophages drives gastric cancer metastasis. Clin Cancer Res. 2019;25(11):3317‐3328. 30796034 10.1158/1078-0432.CCR-18-3567 PMC8955044

[243] Wang Z , Wang Z , Li G , et al. CXCL1 from tumor‐associated lymphatic endothelial cells drives gastric cancer cell into lymphatic system via activating integrin β1/FAK/AKT signaling. Cancer Lett. 2017;385:28‐38. 27832972 10.1016/j.canlet.2016.10.043

[244] Wei ZW , Xia GK , Wu Y , et al. CXCL1 promotes tumor growth through VEGF pathway activation and is associated with inferior survival in gastric cancer. Cancer Lett. 2015;359(2):335‐343. 25641338 10.1016/j.canlet.2015.01.033

[245] Ma J , Su H , Yu B , et al. CXCL12 gene silencing down‐regulates metastatic potential via blockage of MAPK/PI3K/AP‐1 signaling pathway in colon cancer. Clin Transl Oncol. 2018;20(8):1035‐1045. 29305742 10.1007/s12094-017-1821-0 PMC6061162

[246] Ma JC , Sun XW , Su H , et al. Fibroblast‐derived CXCL12/SDF‐1α promotes CXCL6 secretion and co‐operatively enhances metastatic potential through the PI3K/Akt/mTOR pathway in colon cancer. World J Gastroenterol. 2017;23(28):5167‐5178. 28811711 10.3748/wjg.v23.i28.5167 PMC5537183

[247] Yu X , Wang D , Wang X , et al. CXCL12/CXCR4 promotes inflammation‐driven colorectal cancer progression through activation of RhoA signaling by sponging miR‐133a‐3p. J Exp Clin Cancer Res. 2019;38(1):32. 30678736 10.1186/s13046-018-1014-x PMC6346552

[248] Song ZY , Gao ZH , Chu JH , Han XZ , Qu XJ. Downregulation of the CXCR4/CXCL12 axis blocks the activation of the Wnt/β‐catenin pathway in human colon cancer cells. Biomed Pharmacother. 2015;71:46‐52. 25960214 10.1016/j.biopha.2015.01.020

[249] Wang D , Jiao C , Zhu Y , et al. Activation of CXCL12/CXCR4 renders colorectal cancer cells less sensitive to radiotherapy via up‐regulating the expression of survivin. Exp Biol Med(Maywood). 2017;242(4):429‐435. 27798120 10.1177/1535370216675068 PMC5298539

[250] Martinez‐Ordoñez A , Seoane S , Cabezas P , et al. Breast cancer metastasis to liver and lung is facilitated by Pit‐1‐CXCL12‐CXCR4 axis. Oncogene. 2018;37(11):1430‐1444. 29321662 10.1038/s41388-017-0036-8

[251] Sobolik T , Su YJ , Wells S , Ayers GD , Cook RS , Richmond A. CXCR4 drives the metastatic phenotype in breast cancer through induction of CXCR2 and activation of MEK and PI3K pathways. Mol Biol Cell. 2014;25(5):566‐582. 24403602 10.1091/mbc.E13-07-0360 PMC3937084

[252] Luo N , Chen DD , Liu L , Li L , Cheng ZP. CXCL12 promotes human ovarian cancer cell invasion through suppressing ARHGAP10 expression. Biochem Biophys Res Commun. 2019;518(3):416‐422. 31445707 10.1016/j.bbrc.2019.07.098

[253] Chiaramonte R , Colombo M , Bulfamante G , et al. Notch pathway promotes ovarian cancer growth and migration via CXCR4/SDF1α chemokine system. Int J Biochem Cell Biol. 2015;66:134‐140. 26235278 10.1016/j.biocel.2015.07.015

[254] Wang M , Lin T , Wang Y , et al. CXCL12 suppresses cisplatin‐induced apoptosis through activation of JAK2/STAT3 signaling in human non‐small‐cell lung cancer cells. Onco Targets Ther. 2017;10:3215‐3224. 28721072 10.2147/OTT.S133055 PMC5499863

[255] Rodríguez‐Nieves JA , Patalano SC , Almanza D , Gharaee‐Kermani M , Macoska JA. CXCL12/CXCR4 axis activation mediates prostate myofibroblast phenoconversion through non‐canonical EGFR/MEK/ERK signaling. PLoS One. 2016;11(7):e0159490. 27434301 10.1371/journal.pone.0159490 PMC4951124

[256] Uygur B , Wu WS. SLUG promotes prostate cancer cell migration and invasion via CXCR4/CXCL12 axis. Mol Cancer. 2011;10:139. 22074556 10.1186/1476-4598-10-139 PMC3226635

[257] Cheng Y , Qu J , Che X , et al. CXCL12/SDF‐1α induces migration via SRC‐mediated CXCR4‐EGFR cross‐talk in gastric cancer cells. Oncol Lett. 2017;14(2):2103‐2110. 28781651 10.3892/ol.2017.6389 PMC5530148

[258] Izumi D , Ishimoto T , Miyake K , et al. CXCL12/CXCR4 activation by cancer‐associated fibroblasts promotes integrin β1 clustering and invasiveness in gastric cancer. Int J Cancer. 2016;138(5):1207‐1219. 26414794 10.1002/ijc.29864

[259] Chen G , Chen SM , Wang X , Ding XF , Ding J , Meng LH. Inhibition of chemokine(CXC motif) ligand 12/chemokine(CXC motif) receptor 4 axis(CXCL12/CXCR4)‐mediated cell migration by targeting mammalian target of rapamycin(mTOR) pathway in human gastric carcinoma cells. J Biol Chem. 2012;287(15):12132‐12141. 22337890 10.1074/jbc.M111.302299 PMC3320958

[260] Chen F , Yang D , Ru Y , Cao S , Gao A. MicroRNA‐101 Targets CXCL12‐mediated akt and snail signaling pathways to inhibit cellular proliferation and invasion in papillary thyroid carcinoma. Oncol Res. 2019;27(6):691‐701. 30832753 10.3727/096504018X15426763753594 PMC7848424

[261] Lin C , He H , Liu H , et al. Tumour‐associated macrophages‐derived CXCL8 determines immune evasion through autonomous PD‐L1 expression in gastric cancer. Gut. 2019;68(10):1764‐1773. 30661053 10.1136/gutjnl-2018-316324

[262] Luppi F , Longo AM , de Boer WI , Rabe KF , Hiemstra PS. Interleukin‐8 stimulates cell proliferation in non‐small cell lung cancer through epidermal growth factor receptor transactivation. Lung Cancer. 2007;56(1):25‐33. 17175059 10.1016/j.lungcan.2006.11.014

[263] Singh JK , Simões BM , Clarke RB , Bundred NJ. Targeting IL‐8 signalling to inhibit breast cancer stem cell activity. Expert Opin Ther Targets. 2013;17(11):1235‐1241. 24032691 10.1517/14728222.2013.835398

[264] Luca M , Huang S , Gershenwald JE , Singh RK , Reich R , Bar‐Eli M. Expression of interleukin‐8 by human melanoma cells up‐regulates MMP‐2 activity and increases tumor growth and metastasis. Am J Pathol. 1997;151(4):1105‐1113. 9327744 PMC1858026

[265] Zheng T , Ma G , Tang M , Li Z , Xu R. IL‐8 Secreted from M2 macrophages promoted prostate tumorigenesis via STAT3/MALAT1 pathway. Int J Mol Sci. 2018;20(1):98. 10.3390/ijms20010098 PMC6337597 30591689

[266] Xiao YC , Yang ZB , Cheng XS , et al. CXCL8, overexpressed in colorectal cancer, enhances the resistance of colorectal cancer cells to anoikis. Cancer Lett. 2015;361(1):22‐32. 25687885 10.1016/j.canlet.2015.02.021

[267] Sun Q , Sun F , Wang B , et al. Interleukin‐8 promotes cell migration through integrin αvβ6 upregulation in colorectal cancer. Cancer Lett. 2014;354(2):245‐253. 25150782 10.1016/j.canlet.2014.08.021

[268] Itoh Y , Joh T , Tanida S , et al. IL‐8 promotes cell proliferation and migration through metalloproteinase‐cleavage proHB‐EGF in human colon carcinoma cells. Cytokine. 2005;29(6):275‐282. 15749028 10.1016/j.cyto.2004.11.005

[269] Tardáguila M , Mira E , García‐Cabezas MA , et al. CX3CL1 promotes breast cancer via transactivation of the EGF pathway. Cancer Res. 2013;73(14):4461‐4473. 23720051 10.1158/0008-5472.CAN-12-3828 PMC4533861

[270] Liang Y , Yi L , Liu P , et al. CX3CL1 involves in breast cancer metastasizing to the spine via the Src/FAK signaling pathway. J Cancer. 2018;9(19):3603‐3612. 30310518 10.7150/jca.26497 PMC6171022

[271] Liu W , Liang Y , Chan Q , Jiang L , Dong J. CX3CL1 promotes lung cancer cell migration and invasion via the Src/focal adhesion kinase signaling pathway. Oncol Rep. 2019;41(3):1911‐1917. 30628679 10.3892/or.2019.6957

[272] Su YC , Chang H , Sun SJ , et al. Differential impact of CX3CL1 on lung cancer prognosis in smokers and non‐smokers. Mol Carcinog. 2018;57(5):629‐639. 29380447 10.1002/mc.22787

[273] Singh SK , Mishra MK , Singh R. Hypoxia‐inducible factor‐1α induces CX3CR1 expression and promotes the epithelial to mesenchymal transition(EMT) in ovarian cancer cells. J Ovarian Res. 2019;12(1):42. 31077234 10.1186/s13048-019-0517-1 PMC6511167

[274] Tang J , Xiao L , Cui R , et al. CX3CL1 increases invasiveness and metastasis by promoting epithelial‐to‐mesenchymal transition through the TACE/TGF‐α/EGFR pathway in hypoxic androgen‐independent prostate cancer cells. Oncol Rep. 2016;35(2):1153‐1162. 26718770 10.3892/or.2015.4470

[275] Liu P , Liang Y , Jiang L , Wang H , Wang S , Dong J. CX3CL1/fractalkine enhances prostate cancer spinal metastasis by activating the Src/FAK pathway. Int J Oncol. 2018;53(4):1544‐1556. 30066854 10.3892/ijo.2018.4487 PMC6086625

[276] Ren H , Zhao T , Sun J , et al. The CX3CL1/CX3CR1 reprograms glucose metabolism through HIF‐1 pathway in pancreatic adenocarcinoma. J Cell Biochem. 2013;114(11):2603‐2611. 23857671 10.1002/jcb.24608

[277] Geismann C , Erhart W , Grohmann F , et al. TRAIL/NF‐κB/CX3CL1 mediated onco‐immuno crosstalk leading to TRAIL resistance of pancreatic cancer cell lines. Int J Mol Sci. 2018;19(6):1661. 10.3390/ijms19061661 PMC6032098 29867042

[278] Stout MC , Narayan S , Pillet ES , Salvino JM , Campbell PM. Inhibition of CX(3)CR1 reduces cell motility and viability in pancreatic adenocarcinoma epithelial cells. Biochem Biophys Res Commun. 2018;495(3):2264‐2269. 29274778 10.1016/j.bbrc.2017.12.116

[279] Huang LY , Chen P , Xu LX , Zhou YF , Zhang YP , Yuan YZ. Fractalkine upregulates inflammation through CX3CR1 and the Jak‐Stat pathway in severe acute pancreatitis rat model. Inflammation. 2012;35(3):1023‐1030. 22213034 10.1007/s10753-011-9406-5

[280] Quail DF , Bowman RL , Akkari L , et al. The tumor microenvironment underlies acquired resistance to CSF‐1R inhibition in gliomas. Science. 2016;352(6288):aad3018. 27199435 10.1126/science.aad3018 PMC5450629

[281] Sossey‐Alaoui K , Pluskota E , Bialkowska K , et al. Kindlin‐2 regulates the growth of breast cancer tumors by activating CSF‐1‐mediated macrophage infiltration. Cancer Res. 2017;77(18):5129‐5141. 28687620 10.1158/0008-5472.CAN-16-2337 PMC5600848

[282] Lu X , Meng T. Depletion of tumor‐associated macrophages enhances the anti‐tumor effect of docetaxel in a murine epithelial ovarian cancer. Immunobiology. 2019;224(3):355‐361. 30926154 10.1016/j.imbio.2019.03.002

[283] Li M , Li M , Yang Y , et al. Remodeling tumor immune microenvironment via targeted blockade of PI3K‐γ and CSF‐1/CSF‐1R pathways in tumor associated macrophages for pancreatic cancer therapy. J Control Release. 2020;321:23‐35. 32035193 10.1016/j.jconrel.2020.02.011

[284] Gurusamy D , Gray JK , Pathrose P , Kulkarni RM , Finkleman FD , Waltz SE. Myeloid‐specific expression of Ron receptor kinase promotes prostate tumor growth. Cancer Res. 2013;73(6):1752‐1763. 23328584 10.1158/0008-5472.CAN-12-2474 PMC3602275

[285] Babicky ML , Harper MM , Chakedis J , et al. MST1R kinase accelerates pancreatic cancer progression via effects on both epithelial cells and macrophages. Oncogene. 2019;38(28):5599‐5611. 30967626 10.1038/s41388-019-0811-9 PMC6625868

[286] Penny HL , Sieow JL , Adriani G , et al. Warburg metabolism in tumor‐conditioned macrophages promotes metastasis in human pancreatic ductal adenocarcinoma. Oncoimmunology. 2016;5(8):e1191731. 27622062 10.1080/2162402X.2016.1191731 PMC5007961

[287] Chen P , Zuo H , Xiong H , et al. Gpr132 sensing of lactate mediates tumor‐macrophage interplay to promote breast cancer metastasis. Proc Natl Acad Sci U S A. 2017;114(3):580‐585. 28049847 10.1073/pnas.1614035114 PMC5255630

[288] Mantovani A , Locati M. Macrophage metabolism shapes angiogenesis in tumors. Cell Metab. 2016;24(6):887‐888. 27974181 10.1016/j.cmet.2016.11.007

[289] Bohn T , Rapp S , Luther N , et al. Tumor immunoevasion via acidosis‐dependent induction of regulatory tumor‐associated macrophages. Nat Immunol. 2018;19(12):1319‐1329. 30397348 10.1038/s41590-018-0226-8

[290] Zheng X , Mansouri S , Krager A , et al. Metabolism in tumour‐associated macrophages: a quid pro quo with the tumour microenvironment. Eur Respir Rev. 2020;29(157):200134. 10.1183/16000617.0134-2020 PMC9488699 33004525

[291] Colegio OR , Chu NQ , Szabo AL , et al. Functional polarization of tumour‐associated macrophages by tumour‐derived lactic acid. Nature. 2014;513(7519):559‐563. 25043024 10.1038/nature13490 PMC4301845

[292] Prima V , Kaliberova LN , Kaliberov S , Curiel DT , Kusmartsev S. COX2/mPGES1/PGE2 pathway regulates PD‐L1 expression in tumor‐associated macrophages and myeloid‐derived suppressor cells. Proc Natl Acad Sci U S A. 2017;114(5):1117‐1122. 28096371 10.1073/pnas.1612920114 PMC5293015

[293] Zhang Q , Wang H , Mao C , et al. Fatty acid oxidation contributes to IL‐1β secretion in M2 macrophages and promotes macrophage‐mediated tumor cell migration. Mol Immunol. 2018;94:27‐35. 29248877 10.1016/j.molimm.2017.12.011 PMC5801116

[294] Wu L , Zhang X , Zheng L , et al. RIPK3 Orchestrates fatty acid metabolism in tumor‐associated macrophages and hepatocarcinogenesis. Cancer Immunol Res. 2020;8(5):710‐721. 32122992 10.1158/2326-6066.CIR-19-0261

[295] Hao J , Yan F , Zhang Y , et al. Expression of adipocyte/macrophage fatty acid‐binding protein in tumor‐associated macrophages promotes breast cancer progression. Cancer Res. 2018;78(9):2343‐2355. 29437708 10.1158/0008-5472.CAN-17-2465 PMC5932212

[296] Niu Z , Shi Q , Zhang W , et al. Caspase‐1 cleaves PPARγ for potentiating the pro‐tumor action of TAMs. Nat Commun. 2017;8(1):766. 28974683 10.1038/s41467-017-00523-6 PMC5626701

[297] Deng X , Zhang P , Liang T , Deng S , Chen X , Zhu L. Ovarian cancer stem cells induce the M2 polarization of macrophages through the PPARγ and NF‐κB pathways. Int J Mol Med. 2015;36(2):449‐454. 26035689 10.3892/ijmm.2015.2230

[298] Palmieri EM , Menga A , Martín‐Pérez R , et al. Pharmacologic or genetic targeting of glutamine synthetase skews macrophages toward an M1‐like phenotype and inhibits tumor metastasis. Cell Rep. 2017;20(7):1654‐1666. 28813676 10.1016/j.celrep.2017.07.054 PMC5575233

[299] Santhanam S , Alvarado DM , Ciorba MA. Therapeutic targeting of inflammation and tryptophan metabolism in colon and gastrointestinal cancer. Transl Res. 2016;167(1):67‐79. 26297050 10.1016/j.trsl.2015.07.003 PMC4684437

[300] Mazzone M , Menga A , Castegna A. Metabolism and TAM functions‐it takes two to tango. FEBS J. 2018;285(4):700‐716. 29055087 10.1111/febs.14295

[301] Massi D , Marconi C , Franchi A , et al. Arginine metabolism in tumor‐associated macrophages in cutaneous malignant melanoma: evidence from human and experimental tumors. Hum Pathol. 2007;38(10):1516‐1525. 17640716 10.1016/j.humpath.2007.02.018

[302] Chang CI , Liao JC , Kuo L. Macrophage arginase promotes tumor cell growth and suppresses nitric oxide‐mediated tumor cytotoxicity. Cancer Res. 2001;61(3):1100‐1106. 11221839

[303] Dong D , Zhang G , Yang J , et al. The role of iron metabolism in cancer therapy focusing on tumor‐associated macrophages. J Cell Physiol. 2019;234(6):8028‐8039. 30362549 10.1002/jcp.27569

[304] Jung M , Mertens C , Brüne B. Macrophage iron homeostasis and polarization in the context of cancer. Immunobiology. 2015;220(2):295‐304. 25260218 10.1016/j.imbio.2014.09.011

[305] Nasser MW , Wani NA , Ahirwar DK , et al. RAGE mediates S100A7‐induced breast cancer growth and metastasis by modulating the tumor microenvironment. Cancer Res. 2015;75(6):974‐985. 25572331 10.1158/0008-5472.CAN-14-2161 PMC4359968

[306] Chen X , Zhang L , Zhang IY , et al. RAGE expression in tumor‐associated macrophages promotes angiogenesis in glioma. Cancer Res. 2014;74(24):7285‐7297. 25326491 10.1158/0008-5472.CAN-14-1240 PMC4268204

[307] Haase‐Kohn C , Wolf S , Herwig N , Mosch B , Pietzsch J. Metastatic potential of B16‐F10 melanoma cells is enhanced by extracellular S100A4 derived from RAW264.7 macrophages. Biochem Biophys Res Commun. 2014;446(1):143‐148. 24613382 10.1016/j.bbrc.2014.02.126

[308] Wang D , Wang X , Si M , et al. Exosome‐encapsulated miRNAs contribute to CXCL12/CXCR4‐induced liver metastasis of colorectal cancer by enhancing M2 polarization of macrophages. Cancer Lett. 2020;474:36‐52. 31931030 10.1016/j.canlet.2020.01.005

[309] Hu YB , Yan C , Mu L , et al. Exosomal Wnt‐induced dedifferentiation of colorectal cancer cells contributes to chemotherapy resistance. Oncogene. 2019;38(11):1951‐1965. 30390075 10.1038/s41388-018-0557-9 PMC6756234

[310] Gerloff D , Lützkendorf J , Moritz RKC , et al. Melanoma‐derived exosomal miR‐125b‐5p educates tumor associated macrophages (TAMs) by targeting lysosomal acid Lipase A(LIPA). Cancers(Basel). 2020;12(2):464. 10.3390/cancers12020464 PMC7072270 32079286

[311] Wang X , Luo G , Zhang K , et al. Hypoxic tumor‐derived exosomal miR‐301a mediates M2 macrophage polarization via PTEN/PI3Kγ to promote pancreatic cancer metastasis. Cancer Res. 2018;78(16):4586‐4598. 29880482 10.1158/0008-5472.CAN-17-3841

[312] Yin Z , Ma T , Huang B , et al. Macrophage‐derived exosomal microRNA‐501‐3p promotes progression of pancreatic ductal adenocarcinoma through the TGFBR3‐mediated TGF‐β signaling pathway. J Exp Clin Cancer Res. 2019;38(1):310. 31307515 10.1186/s13046-019-1313-x PMC6631643

[313] Zhu X , Shen H , Yin X , et al. Macrophages derived exosomes deliver miR‐223 to epithelial ovarian cancer cells to elicit a chemoresistant phenotype. J Exp Clin Cancer Res. 2019;38(1): 81. 30770776 10.1186/s13046-019-1095-1 PMC6377760

[314] Guan H , Peng R , Fang F , et al. Tumor‐associated macrophages promote prostate cancer progression via exosome‐mediated miR‐95 transfer. J Cell Physiol. 2020;235(12):9729‐9742. 32406953 10.1002/jcp.29784

[315] Lu X , Kang Y. Chemokine(C‐C motif) ligand 2 engages CCR2+ stromal cells of monocytic origin to promote breast cancer metastasis to lung and bone. J Biol Chem. 2009;284(42):29087‐29096. 19720836 10.1074/jbc.M109.035899 PMC2781454

[316] Loberg RD , Ying C , Craig M , et al. CCL2, Prostate cancer, docetaxel, CNTO888. Cancer Res. 2007;67(19):9417‐9424. 17909051 10.1158/0008-5472.CAN-07-1286

[317] Sanford DE , Belt BA , Panni RZ , et al. Inflammatory monocyte mobilization decreases patient survival in pancreatic cancer: a role for targeting the CCL2/CCR2 axis. Clin Cancer Res. 2013;19(13):3404‐3415. 23653148 10.1158/1078-0432.CCR-13-0525 PMC3700620

[318] Farina S , Yang H , Tu G , et al. Targeting tumor associated myeloid cells with CCR2 inhibitor PF‐04136309 enhances gemcitabine/paclitaxel and doxorubicin anti‐tumor activity. Cancer Res. 2017;77:LB‐194.

[319] Nywening TM , Wang‐Gillam A , Sanford DE , et al. Targeting tumour‐associated macrophages with CCR2 inhibition in combination with FOLFIRINOX in patients with borderline resectable and locally advanced pancreatic cancer: a single‐centre, open‐label, dose‐finding, non‐randomised, phase 1b trial. Lancet Oncol. 2016;17(5):651‐662. 27055731 10.1016/S1470-2045(16)00078-4 PMC5407285

[320] Duliege A‐M , Sleijfer S , Bischof A , et al. CCX872: Pharmacodynamic study of a potent and selective CCR2 antagonist in human volunteers and plans for phase Ib trial in patients with pancreatic cancer. Cancer Res. 2015;75:CT223‐CT223.

[321] Yao W , Ba Q , Li X , et al. A Natural CCR2 Antagonist relieves tumor‐associated macrophage‐mediated immunosuppression to produce a therapeutic effect for liver cancer. EBioMedicine. 2017;22:58‐67. 28754304 10.1016/j.ebiom.2017.07.014 PMC5552238

[322] Li X , Yao W , Yuan Y , et al. Targeting of tumour‐infiltrating macrophages via CCL2/CCR2 signalling as a therapeutic strategy against hepatocellular carcinoma. Gut. 2017;66(1):157‐167. 26452628 10.1136/gutjnl-2015-310514

[323] Pienta KJ , Machiels JP , Schrijvers D , et al. Phase 2 study of carlumab(CNTO 888), a human monoclonal antibody against CC‐chemokine ligand 2(CCL2), in metastatic castration‐resistant prostate cancer. Invest New Drugs. 2013;31(3):760‐768. 22907596 10.1007/s10637-012-9869-8

[324] Cambien B , Richard‐Fiardo P , Karimdjee BF , et al. CCL5 neutralization restricts cancer growth and potentiates the targeting of PDGFRbeta in colorectal carcinoma. PLoS One. 2011;6(12):e28842. 22205974 10.1371/journal.pone.0028842 PMC3243667

[325] Pervaiz A , Zepp M , Mahmood S , Ali DM , Berger MR , Adwan H. CCR5 blockage by maraviroc: a potential therapeutic option for metastatic breast cancer. Cell Oncol(Dordr). 2019;42(1):93‐106. 30456574 10.1007/s13402-018-0415-3 PMC12994360

[326] Mencarelli A , Graziosi L , Renga B , et al. CCR5 antagonism by maraviroc reduces the potential for gastric cancer cell dissemination. Transl Oncol. 2013;6(6):784‐793. 24466382 10.1593/tlo.13499 PMC3890714

[327] Zhang X , Haney KM , Richardson AC , et al. Anibamine, a natural product CCR5 antagonist, as a novel lead for the development of anti‐prostate cancer agents. Bioorg Med Chem Lett. 2010;20(15):4627‐4630. 20579875 10.1016/j.bmcl.2010.06.003 PMC2914538

[328] Yost R , Pasquale TR , Sahloff EG. Maraviroc: a coreceptor CCR5 antagonist for management of HIV infection. Am J Health Syst Pharm. 2009;66(8):715‐726. 19336831 10.2146/ajhp080206

[329] Pervaiz A , Ansari S , Berger MR , Adwan H. CCR5 blockage by maraviroc induces cytotoxic and apoptotic effects in colorectal cancer cells. Med Oncol. 2015;32(5):158. 25840792 10.1007/s12032-015-0607-x

[330] Huang H , Zepp M , Georges RB , et al. The CCR5 antagonist maraviroc causes remission of pancreatic cancer liver metastasis in nude rats based on cell cycle inhibition and apoptosis induction. Cancer Lett. 2020;474:82‐93. 31954769 10.1016/j.canlet.2020.01.009

[331] Aquaro S , Hatse S , Princen K , et al. Potent anti‐HIV‐1 activity of TAK‐779 in human primary macrophages. Antivir Res. 2002;53:A45‐A45.

[332] Menu E , De Leenheer E , De Raeve H , et al. Role of CCR1 and CCR5 in homing and growth of multiple myeloma and in the development of osteolytic lesions: a study in the 5TMM model. Clin Exp Metastasis. 2006;23(5‐6):291‐300. 17086356 10.1007/s10585-006-9038-6

[333] Ni J , Zhu YN , Zhong XG , et al. The chemokine receptor antagonist, TAK‐779, decreased experimental autoimmune encephalomyelitis by reducing inflammatory cell migration into the central nervous system, without affecting T cell function. Br J Pharmacol. 2009;158(8):2046‐2056. 20050195 10.1111/j.1476-5381.2009.00528.x PMC2807666

[334] Tan MC , Goedegebuure PS , Belt BA , et al. Disruption of CCR5‐dependent homing of regulatory T cells inhibits tumor growth in a murine model of pancreatic cancer. J Immunol. 2009;182(3):1746‐1755. 19155524 10.4049/jimmunol.182.3.1746 PMC3738070

[335] Zhang F , Arnatt CK , Haney KM , et al. Structure activity relationship studies of natural product chemokine receptor CCR5 antagonist anibamine toward the development of novel anti prostate cancer agents. Eur J Med Chem. 2012;55:395‐408. 22901310 10.1016/j.ejmech.2012.07.049

[336] Li G , Haney KM , Kellogg GE , Zhang Y. Comparative docking study of anibamine as the first natural product CCR5 antagonist in CCR5 homology models. J Chem Inf Model. 2009;49(1):120‐132. 19166361 10.1021/ci800356a PMC2656111

[337] Moreb JS. Plerixafor in non‐Hodgkin's lymphoma and multiple myeloma patients undergoing autologous stem cell transplantation. Oncology Rev. 2011;5(1):67‐73.

[338] Szczucinski A , Losy J. Chemokines and chemokine receptors in multiple sclerosis. Potential targets for new therapies. Acta Neurol Scand. 2007;115(3):137‐146. 17295707 10.1111/j.1600-0404.2006.00749.x

[339] Nervi B , Ramirez P , Rettig MP , et al. Chemosensitization of acute myeloid leukemia(AML) following mobilization by the CXCR4 antagonist AMD3100. Blood. 2009;113(24):6206‐6214. 19050309 10.1182/blood-2008-06-162123 PMC2699239

[340] Feig C , Jones JO , Kraman M , et al. Targeting CXCL12 from FAP‐expressing carcinoma‐associated fibroblasts synergizes with anti‐PD‐L1 immunotherapy in pancreatic cancer. Proc Natl Acad Sci U S A. 2013;110(50):20212‐20217. 24277834 10.1073/pnas.1320318110 PMC3864274

[341] Ling X , Spaeth E , Chen Y , et al. The CXCR4 antagonist AMD3465 regulates oncogenic signaling and invasiveness in vitro and prevents breast cancer growth and metastasis in vivo. PLoS One. 2013;8(3):e58426. 23484027 10.1371/journal.pone.0058426 PMC3590173

[342] Peng SB , Zhang X , Paul D , et al. Identification of LY2510924, a novel cyclic peptide CXCR4 antagonist that exhibits antitumor activities in solid tumor and breast cancer metastatic models. Mol Cancer Ther. 2015;14(2):480‐490. 25504752 10.1158/1535-7163.MCT-14-0850

[343] Galsky MD , Vogelzang NJ , Conkling P , et al. A phase I trial of LY2510924, a CXCR4 peptide antagonist, in patients with advanced cancer. Clin Cancer Res. 2014;20(13):3581‐3588. 24727324 10.1158/1078-0432.CCR-13-2686

[344] Beider K , Ribakovsky E , Abraham M , et al. Targeting the CD20 and CXCR4 pathways in non‐hodgkin lymphoma with rituximab and high‐affinity CXCR4 antagonist BKT140. Clin Cancer Res. 2013;19(13):3495‐3507. 23637121 10.1158/1078-0432.CCR-12-3015

[345] Gravina GL , Mancini A , Marampon F , et al. The brain‐penetrating CXCR4 antagonist, PRX177561, increases the antitumor effects of bevacizumab and sunitinib in preclinical models of human glioblastoma. J Hematol Oncol. 2017;10(1):5. 28057017 10.1186/s13045-016-0377-8 PMC5217647

[346] Gagner JP , Sarfraz Y , Ortenzi V , et al. Multifaceted C‐X‐C chemokine receptor 4(CXCR4) inhibition interferes with anti‐vascular endothelial growth factor therapy‐induced glioma dissemination. Am J Pathol. 2017;187(9):2080‐2094. 28734730 10.1016/j.ajpath.2017.04.020 PMC5809520

[347] Kim SY , Lee CH , Midura BV , et al. Inhibition of the CXCR4/CXCL12 chemokine pathway reduces the development of murine pulmonary metastases. Clin Exp Metastasis. 2008;25(3):201‐211. 18071913 10.1007/s10585-007-9133-3 PMC2730112

[348] Porvasnik S , Sakamoto N , Kusmartsev S , et al. Effects of CXCR4 antagonist CTCE‐9908 on prostate tumor growth. Prostate. 2009;69(13):1460‐1469. 19588526 10.1002/pros.21008

[349] Murakami T , Maki W , Cardones AR , et al. Expression of CXC chemokine receptor‐4 enhances the pulmonary metastatic potential of murine B16 melanoma cells. Cancer Res. 2002;62(24):7328‐7334. 12499276

[350] Di Cesare S , Marshall JC , Fernandes BF , et al. In vitro characterization and inhibition of the CXCR4/CXCL12 chemokine axis in human uveal melanoma cell lines. Cancer Cell Int. 2007;7:17. 18001467 10.1186/1475-2867-7-17 PMC2194662

[351] Liang Z , Wu T , Lou H , et al. Inhibition of breast cancer metastasis by selective synthetic polypeptide against CXCR4. Cancer Res. 2004;64(12):4302‐4308. 15205345 10.1158/0008-5472.CAN-03-3958

[352] Richert MM , Vaidya KS , Mills CN , et al. Inhibition of CXCR4 by CTCE‐9908 inhibits breast cancer metastasis to lung and bone. Oncol Rep. 2009;21(3):761‐767. 19212637 PMC13435714

[353] Wong D , Kandagatla P , Korz W , Chinni SR. Targeting CXCR4 with CTCE‐9908 inhibits prostate tumor metastasis. BMC Urol. 2014;14:12. 24472670 10.1186/1471-2490-14-12 PMC3912255

[354] Schott AF , Goldstein LJ , Cristofanilli M , et al. Phase Ib pilot study to evaluate Reparixin in combination with weekly Paclitaxel in patients with HER‐2‐negative metastatic breast cancer. Clin Cancer Res. 2017;23(18):5358‐5365. 28539464 10.1158/1078-0432.CCR-16-2748 PMC5600824

[355] Kim YH , Bagot M , Pinter‐Brown L , et al. Mogamulizumab versus vorinostat in previously treated cutaneous T‐cell lymphoma(MAVORIC): an international, open‐label, randomised, controlled phase 3 trial. Lancet Oncol. 2018;19(9):1192‐1204. 30100375 10.1016/S1470-2045(18)30379-6

[356] Ishida T , Utsunomiya A , Jo T , et al. Mogamulizumab for relapsed adult T‐cell leukemia‐lymphoma: updated follow‐up analysis of phase I and II studies. Cancer Sci. 2017;108(10):2022‐2029. 28776876 10.1111/cas.13343 PMC5623751

[357] Yano H , Ishida T , Inagaki A , et al. Defucosylated anti CC chemokine receptor 4 monoclonal antibody combined with immunomodulatory cytokines: a novel immunotherapy for aggressive/refractory Mycosis fungoides and Sezary syndrome. Clin Cancer Res. 2007;13(21):6494‐6500. 17975162 10.1158/1078-0432.CCR-07-1324

[358] Schroeder MA , Rettig MP , Lopez S , et al. Mobilization of allogeneic peripheral blood stem cell donors with intravenous plerixafor mobilizes a unique graft. Blood. 2017;129(19):2680‐2692. 28292947 10.1182/blood-2016-09-739722 PMC5428459

[359] Uy GL , Rettig MP , Motabi IH , et al. A phase 1/2 study of chemosensitization with the CXCR4 antagonist plerixafor in relapsed or refractory acute myeloid leukemia. Blood. 2012;119(17):3917‐3924. 22308295 10.1182/blood-2011-10-383406 PMC3350358

[360] Salgia R , Stille JR , Weaver RW , et al. A randomized phase II study of LY2510924 and carboplatin/etoposide versus carboplatin/etoposide in extensive‐disease small cell lung cancer. Lung Cancer. 2017;105:7‐13. 28236984 10.1016/j.lungcan.2016.12.020

[361] Pernas S , Martin M , Kaufman PA , et al. Balixafortide plus eribulin in HER2‐negative metastatic breast cancer: a phase 1, single‐arm, dose‐escalation trial. Lancet Oncol. 2018;19(6):812‐824. 29706375 10.1016/S1470-2045(18)30147-5

[362] Hélène Haegel CT , Hallet R , Geist M , et al. A unique anti‐CD115 monoclonal antibody which inhibits osteolysis and skews human monocyte differentiation from M2‐polarized macrophages toward dendritic cells. MAbs. 2013;5(5):736‐747. 23924795 10.4161/mabs.25743 PMC3851226

[363] Mohammed E‐G , K A‐AS , M A‐KD , G HM . Recent advances of colony‐stimulating factor‐1 receptor(CSF‐1R) kinase and its inhibitors. J Med Chem. 2018;61(13):5450‐5466. 29293000 10.1021/acs.jmedchem.7b00873

[364] Ries CH , Cannarile MA , Hoves S , et al. Targeting tumor‐associated macrophages with anti‐CSF‐1R antibody reveals a strategy for cancer therapy. Cancer Cell. 2014;25(6):846‐859. 24898549 10.1016/j.ccr.2014.05.016

[365] Cassier PA , Italiano A , Gomez‐Roca CA , et al. CSF1R inhibition with emactuzumab in locally advanced diffuse‐type tenosynovial giant cell tumours of the soft tissue: a dose‐escalation and dose‐expansion phase 1 study. Lancet Oncol. 2015;16(8):949‐956. 26179200 10.1016/S1470-2045(15)00132-1

[366] Genovese MC , Hsia E , Belkowski SM , et al. Results from a phase IIA parallel group study of JNJ‐40346527, an oral CSF‐1R inhibitor, in patients with active rheumatoid arthritis despite disease‐modifying antirheumatic drug therapy. J Rheumatol. 2015;42(10):1752‐1760. 26233509 10.3899/jrheum.141580

[367] von Tresckow B , Morschhauser F , Ribrag V , et al. An open‐label, multicenter, phase I/II study of JNJ‐40346527, a CSF‐1R inhibitor, in patients with relapsed or refractory Hodgkin lymphoma. Clin Cancer Res. 2015;21(8):1843‐1850. 25628399 10.1158/1078-0432.CCR-14-1845

[368] Conway JG , McDonald B , Parham J , et al. Inhibition of colony‐stimulating‐factor‐1 signaling in vivo with the orally bioavailable cFMS kinase inhibitor GW2580. Proc Natl Acad Sci U S A. 2005;102(44):16078‐16083. 16249345 10.1073/pnas.0502000102 PMC1276040

[369] Priceman SJ , Sung JL , Shaposhnik Z , et al. Targeting distinct tumor‐infiltrating myeloid cells by inhibiting CSF‐1 receptor: combating tumor evasion of antiangiogenic therapy. Blood. 2010;115(7):1461‐1471. 20008303 10.1182/blood-2009-08-237412 PMC2826767

[370] Mitchem JB , Brennan DJ , Knolhoff BL , et al. Targeting tumor‐infiltrating macrophages decreases tumor‐initiating cells, relieves immunosuppression, and improves chemotherapeutic responses. Cancer Res. 2013;73(3):1128‐1141. 23221383 10.1158/0008-5472.CAN-12-2731 PMC3563931

[371] Hatzimichael E , Georgiou G , Benetatos L , Briasoulis E. Gene mutations and molecularly targeted therapies in acute myeloid leukemia. Am J Blood Res. 2013;3(1):29‐51. 23358589 PMC3555190

[372] Butowski N , Colman H , De Groot JF , et al. Orally administered colony stimulating factor 1 receptor inhibitor PLX3397 in recurrent glioblastoma: an Ivy Foundation Early Phase Clinical Trials Consortium phase II study. Neuro Oncol. 2016;18(4):557‐564. 26449250 10.1093/neuonc/nov245 PMC4799682

[373] Tap WD , Wainberg ZA , Anthony SP , et al. Structure‐guided blockade of CSF1R kinase in tenosynovial giant‐cell tumor. N Engl J Med. 2015;373(5):428‐437. 26222558 10.1056/NEJMoa1411366

[374] Butowski N , Colman H , Groot J , et al. A phase 2 study of orally administered PLX3397 in patients with recurrent glioblastoma. ASCO Meeting Abstracts 2014.

[375] Schroeder GM , An Y , Cai ZW , et al. Discovery of N‐(4‐(2‐amino‐3‐chloropyridin‐4‐yloxy)‐3‐fluorophenyl)‐4‐ethoxy‐1‐(4‐fluorophenyl)‐2‐oxo‐1,2‐dihydropyridine‐3‐carboxamide(BMS‐777607), a selective and orally efficacious inhibitor of the Met kinase superfamily. J Med Chem. 2009;52(5):1251‐1254. 19260711 10.1021/jm801586s

[376] Eyob H , Ekiz HA , Derose YS , Waltz SE , Williams MA , Welm AL. Inhibition of ron kinase blocks conversion of micrometastases to overt metastases by boosting antitumor immunity. Cancer Discov. 2013;3(7):751‐760. 23612011 10.1158/2159-8290.CD-12-0480 PMC3710539

[377] Bieniasz M , Radhakrishnan P , Faham N , De La O J‐P , Welm AL. Pre‐clinical efficacy of Ron kinase inhibitors alone and in combination with PI3K inhibitors for treatment of sfRon‐expressing breast cancer patient‐derived xenografts. Clin Cancer Res. 2015;21(24):5588‐5600. 26289070 10.1158/1078-0432.CCR-14-3283 PMC4681594

[378] Gomez‐Roca CA , Italiano A , Le Tourneau C , et al. Phase I study of emactuzumab single agent or in combination with paclitaxel in patients with advanced/metastatic solid tumors reveals depletion of immunosuppressive M2‐like macrophages. Ann Oncol. 2019;30(8):1381‐1392. 31114846 10.1093/annonc/mdz163 PMC8887589

[379] Tap WD , Gelderblom H , Palmerini E , et al. Pexidartinib versus placebo for advanced tenosynovial giant cell tumour(ENLIVEN): a randomised phase 3 trial. Lancet. 2019;394(10197):478‐487. 31229240 10.1016/S0140-6736(19)30764-0 PMC6860022

[380] Smith CC , Levis MJ , Frankfurt O , et al. A phase 1/2 study of the oral FLT3 inhibitor pexidartinib in relapsed/refractory FLT3‐ITD‐mutant acute myeloid leukemia. Blood Adv. 2020;4(8):1711‐1721. 32330242 10.1182/bloodadvances.2020001449 PMC7189289

[381] Diamond JR , Eckhardt SG , Pitts TM , et al. A phase II clinical trial of the Aurora and angiogenic kinase inhibitor ENMD‐2076 for previously treated, advanced, or metastatic triple‐negative breast cancer. Breast Cancer Res. 2018;20(1):82. 30071865 10.1186/s13058-018-1014-y PMC6090978

[382] Roohullah A , Cooper A , Lomax AJ , et al. A phase I trial to determine safety and pharmacokinetics of ASLAN002, an oral MET superfamily kinase inhibitor, in patients with advanced or metastatic solid cancers. Invest New Drugs. 2018;36(5):886‐894. 29766337 10.1007/s10637-018-0588-7

[383] Moro‐Sibilot D , Cozic N , Perol M , et al. Crizotinib in c‐MET‐ or ROS1‐positive NSCLC: results of the AcSe phase II trial. Ann Oncol. 2019;30(12):1985‐1991. 31584608 10.1093/annonc/mdz407

[384] Rayson D , Lupichuk S , Potvin K , et al. Canadian Cancer Trials Group IND197: a phase II study of foretinib in patients with estrogen receptor, progesterone receptor, and human epidermal growth factor receptor 2‐negative recurrent or metastatic breast cancer. Breast Cancer Res Treat. 2016;157(1):109‐116. 27116183 10.1007/s10549-016-3812-1

[385] Choueiri TK , Vaishampayan U , Rosenberg JE , et al. Phase II and biomarker study of the dual MET/VEGFR2 inhibitor foretinib in patients with papillary renal cell carcinoma. J Clin Oncol. 2013;31(2):181‐186. 23213094 10.1200/JCO.2012.43.3383 PMC3532390

[386] Shah MA , Wainberg ZA , Catenacci DV , et al. Phase II study evaluating 2 dosing schedules of oral foretinib(GSK1363089), cMET/VEGFR2 inhibitor, in patients with metastatic gastric cancer. PLoS One. 2013;8(3):e54014. 23516391 10.1371/journal.pone.0054014 PMC3597709

[387] Singh RP , Patel B , Kallender H , Ottesen LH , Adams LM , Cox DS. Population pharmacokinetics modeling and analysis of foretinib in adult patients with advanced solid tumors. J Clin Pharmacol. 2015;55(10):1184‐1192. 25998042 10.1002/jcph.546

[388] Roelofs AJ , Thompson K , Gordon S , Rogers MJ. Molecular mechanisms of action of bisphosphonates: current status. Clin Cancer Res. 2006;12(20 Pt 2):6222s‐6230s. 17062705 10.1158/1078-0432.CCR-06-0843

[389] Stresing V , Fournier PG , Bellahcène A , et al. Nitrogen‐containing bisphosphonates can inhibit angiogenesis in vivo without the involvement of farnesyl pyrophosphate synthase. Bone. 2011;48(2):259‐266. 20920623 10.1016/j.bone.2010.09.035

[390] Junankar S , Shay G , Jurczyluk J , et al. Real‐time intravital imaging establishes tumor‐associated macrophages as the extraskeletal target of bisphosphonate action in cancer. Cancer Discov. 2015;5(1):35‐42. 25312016 10.1158/2159-8290.CD-14-0621 PMC4293349

[391] Pietschmann P , Stohlawetz P , Brosch S , Steiner G , Smolen JS , Peterlik M. The effect of alendronate on cytokine production, adhesion molecule expression, and transendothelial migration of human peripheral blood mononuclear cells. Calcif Tissue Int. 1998;63(4):325‐330. 9744992 10.1007/s002239900535

[392] Thiébaud D , Sauty A , Burckhardt P , et al. AnIn vitroandin vivostudy of cytokines in the acute‐phase response associated with bisphosphonates. Calcified Tissue Int. 1997;61(5):386‐392. 10.1007/s002239900353 9351880

[393] Zysk SP , Durr HR , Gebhard HH , et al. Effects of ibandronate on inflammation in mouse antigen‐induced arthritis. Inflamm Res. 2003;52(5):221‐226. 12813627 10.1007/s000110300075

[394] Dicuonzo G , Vincenzi B , Santini D , Avvisati G , Tonini G. Fever after zoledronic acid administration is due to increase in TNF‐α and IL‐6. J Interferon Cytokine Res. 2003;23(11):649‐654. 14651779 10.1089/107999003322558782

[395] Pennanen N , Lapinjoki S , Urtti A , Mönkkönen J. Effect of liposomal and free bisphosphonates on the IL‐1β, IL‐6 and TNFα secretion from RAW 264 cells in vitro. Pharm Res. 1995;12(6):916‐922. 7667201 10.1023/a:1016281608773

[396] Pallardy MJ , Turbica I , Biola‐Vidamment A. Why the immune system should be concerned by nanomaterials? Front Immunol. 2017;8:544. 28555135 10.3389/fimmu.2017.00544 PMC5431153

[397] Rao L , Zhao SK , Wen C , et al. Activating macrophage‐mediated cancer immunotherapy by genetically edited nanoparticles. Adv Mater. 2020;32(47):e2004853. 33089578 10.1002/adma.202004853 PMC7686299

[398] Elsabahy M , Wooley KL. Cytokines as biomarkers of nanoparticle immunotoxicity. Chem Soc Rev. 2013;42(12):5552‐5576. 23549679 10.1039/c3cs60064e PMC3665642

[399] Sander AA Kooijmans P , Vader SM , van Dommelen WW , van Solinge , Schiffelers RM. Exosome mimetics: a novel class of drug delivery systems. Int J Nanomedicine. 2012;7:1525‐1541. 22619510 10.2147/IJN.S29661 PMC3356169

[400] Hood JL , Wickline SA. A systematic approach to exosome‐based translational nanomedicine. Wiley Interdiscip Rev Nanomed Nanobiotechnol. 2012;4(4):458‐467. 22648975 10.1002/wnan.1174

[401] Kooijmans SAA , Fliervoet LAL , van der Meel R. et al. PEGylated and targeted extracellular vesicles display enhanced cell specificity and circulation time. J Control Release. 2016;224:77‐85. 26773767 10.1016/j.jconrel.2016.01.009

[402] Shin‐ichiro O , Masakatsu T , Katsuko S , et al. Systemically injected exosomes targeted to EGFR deliver antitumor MicroRNA to breast cancer cells. Mol Ther. 2013;21(1):185‐191. 23032975 10.1038/mt.2012.180 PMC3538304

[403] Gomari H , Forouzandeh Moghadam M , Soleimani M . Targeted cancer therapy using engineered exosome as a natural drug delivery vehicle. Onco Targets Ther. 2018;11:5753‐5762. 30254468 10.2147/OTT.S173110 PMC6140699

[404] Wang X , Zhang H , Bai M , et al. Exosomes serve as nanoparticles to deliver anti‐miR‐214 to reverse chemoresistance to cisplatin in gastric cancer. Mol Ther. 2018;26(3):774‐783. 29456019 10.1016/j.ymthe.2018.01.001 PMC5910674

[405] McKelvey KJ , Powell KL , Ashton AW , Morris JM , McCracken SA. Exosomes: mechanisms of uptake. J Circ Biomark. 2015;4:7. 28936243 10.5772/61186 PMC5572985

[406] Zhuang X , Xiang X , Grizzle W , et al. Treatment of brain inflammatory diseases by delivering exosome encapsulated anti‐inflammatory drugs from the nasal region to the brain. Mol Ther. 2011;19(10):1769‐1779. 21915101 10.1038/mt.2011.164 PMC3188748

[407] Su MJ , Aldawsari H , Amiji M. Pancreatic cancer cell exosome‐mediated macrophage reprogramming and the role of microRNAs 155 and 125b2 transfection using nanoparticle delivery systems. Sci Rep. 2016;22(6):30110. 10.1038/srep30110 PMC4957091 27443190

[408] Trivedi M , Talekar M , Shah P , Ouyang Q , Amiji M. Modification of tumor cell exosome content by transfection with wt‐p53 and microRNA‐125b expressing plasmid DNA and its effect on macrophage polarization. Oncogenesis. 2016;5(8):e250. 27500388 10.1038/oncsis.2016.52 PMC5007827

[409] Rayamajhi S , Nguyen TDT , Marasini R , Aryal S. Macrophage‐derived exosome‐mimetic hybrid vesicles for tumor targeted drug delivery. Acta Biomater. 2019;94:482‐494. 31129363 10.1016/j.actbio.2019.05.054

[410] Rodell CB , Arlauckas SP , Cuccarese MF , et al. TLR7/8‐agonist‐loaded nanoparticles promote the polarization of tumour‐associated macrophages to enhance cancer immunotherapy. Nat Biomed Eng. 2018;2:578‐588. 10.1038/s41551-018-0236-8 PMC6192054 31015631

[411] Feng Y , Mu R , Wang Z , et al. A toll‐like receptor agonist mimicking microbial signal to generate tumor‐suppressive macrophages. Nat Commun. 2019;10(1):2272. 31118418 10.1038/s41467-019-10354-2 PMC6531447

[412] Beatty GL , Chiorean EG , Fishman MP , et al. CD40 agonists alter tumor stroma and show efficacy against pancreatic carcinoma in mice and humans. Science. 2011;331(6024):1612‐1616. 21436454 10.1126/science.1198443 PMC3406187

[413] Andrechak JC , Dooling LJ , Discher DE. The macrophage checkpoint CD47 : SIRPalpha for recognition of “self” cells: from clinical trials of blocking antibodies to mechanobiological fundamentals. Philos Trans R Soc Lond B Biol Sci. 2019;374(1779):20180217. 31431181 10.1098/rstb.2018.0217 PMC6627025

[414] Vaeteewoottacharn K , Kariya R , Pothipan P , et al. Attenuation of CD47‐SIRPalpha signal in cholangiocarcinoma potentiates tumor‐associated macrophage‐mediated phagocytosis and suppresses intrahepatic metastasis. Transl Oncol. 2019;12(2):217‐225. 30415063 10.1016/j.tranon.2018.10.007 PMC6231245

[415] Hartley GP , Chow L , Ammons DT , Wheat WH , Dow SW. Programmed cell death ligand 1(PD‐L1) signaling regulates macrophage proliferation and activation. Cancer Immunol Res. 2018;6(10):1260‐1273. 30012633 10.1158/2326-6066.CIR-17-0537

[416] Molgora M , Esaulova E , Vermi W , et al. TREM2 modulation remodels the tumor myeloid landscape enhancing anti‐PD‐1 immunotherapy. Cell. 2020;182(4):886‐900 e817. 32783918 10.1016/j.cell.2020.07.013 PMC7485282

[417] Zhu S , Niu M , O'Mary H , Cui Z. Targeting of tumor‐associated macrophages made possible by PEG‐sheddable, mannose‐modified nanoparticles. Mol Pharm. 2013;10(9):3525–3530. 23901887 10.1021/mp400216r PMC3946577

[418] Xia Y , Xu T , Zhao M , et al. Delivery of doxorubicin for human cervical carcinoma targeting therapy by folic acid‐modified selenium nanoparticles. Int J Mol Sci. 2018;19(11):3582‐3595. 10.3390/ijms19113582 PMC6274826 30428576

[419] Van DB, G J , Schlee M , Coch C , Hartmann G. SiRNA delivery with exosome nanoparticles. Nat Biotechnol. 2011;29(4):325‐326. 21478846 10.1038/nbt.1830

